# Variability in the Effect of 5-HTTLPR on Depression in a Large European Population: The Role of Age, Symptom Profile, Type and Intensity of Life Stressors

**DOI:** 10.1371/journal.pone.0116316

**Published:** 2015-03-06

**Authors:** Gabriella Juhasz, Xenia Gonda, Gabor Hullam, Nora Eszlari, David Kovacs, Judit Lazary, Dorottya Pap, Peter Petschner, Rebecca Elliott, John Francis William Deakin, Ian Muir Anderson, Peter Antal, Klaus-Peter Lesch, Gyorgy Bagdy

**Affiliations:** 1 Department of Pharmacodynamics, Faculty of Pharmacy, Semmelweis University, Budapest, Hungary; 2 MTA-SE Neuropsychopharmacology and Neurochemistry Research Group, Hungarian Academy of Sciences, Semmelweis University, Budapest, Hungary; 3 Neuroscience and Psychiatry Unit, School of Community Based Medicine, Faculty of Medical and Human Sciences, The University of Manchester, Manchester, United Kingdom, and Manchester Academic Health Sciences Centre, Manchester, United Kingdom; 4 Department of Clinical and Theoretical Mental Health, Kutvolgyi Clinical Center, Semmelweis University, Budapest, Hungary; 5 Department of Measurement and Information Systems, Budapest University of Technology and Economics, Budapest, Hungary; 6 Division of Molecular Psychiatry, Department of Psychiatry, Psychosomatics and Psychotherapy, University of Wuerzburg, Wuerzburg, Germany, and Department of Translational Neuroscience, School of Mental Health and Neuroscience (MHENS), Maastricht University, Maastricht, The Netherlands; National Cheng Kung University Medical College, TAIWAN

## Abstract

**Background:**

Although *5-HTTLPR* has been shown to influence the risk of life stress-induced depression in the majority of studies, others have produced contradictory results, possibly due to weak effects and/or sample heterogeneity.

**Methods:**

In the present study we investigated how age, type and intensity of life-stressors modulate the effect of *5-HTTLPR* on depression and anxiety in a European population cohort of over 2300 subjects. Recent negative life events (RLE), childhood adversity (CHA), lifetime depression, Brief Symptoms Inventory (BSI) depression and anxiety scores were determined in each subject. Besides traditional statistical analysis we calculated Bayesian effect strength and relevance of *5-HTTLPR* genotypes in specified models.

**Results:**

The short (s) low expressing allele showed association with increased risk of depression related phenotypes, but all nominally significant effects would turn to non-significant after correction for multiple testing in the traditional analysis. Bayesian effect strength and relevance analysis, however, confirmed the role of *5-HTTLPR*. Regarding current (BSI) and lifetime depression *5-HTTLPR-*by-RLE interactions were confirmed. Main effect, with other words direct association, was supported with BSI anxiety. With more frequent RLE the prevalence or symptoms of depression increased in ss carriers. Although CHA failed to show an interaction with *5-HTTLPR*, in young subjects CHA sensitized towards the depression promoting effect of even mild RLE. Furthermore, the direct association of anxiety with the s allele was driven by young (≤30) individuals.

**Limitations:**

Our study is cross-sectional and applies self-report questionnaires.

**Conclusions:**

Albeit *5-HTTLPR* has only weak/moderate effects, the s allele is directly associated with anxiety and modulates development of depression in homogeneous subgroups.

## Introduction

Depression is well-known to be a polygenic and multifactorial condition, and although it shows a moderate degree of inheritance and genetic factors account for a moderate portion of its variability, the contribution of each individual gene seems to be small and influenced by other genetic and environmental factors [1,2]. One of the most investigated genetic polymorphisms regarding gene-by-environment interaction (GxE) and depression is *5-HTTLPR*, a functional polymorphism in the upstream regulatory region of the serotonin transporter gene (*5-HTT*, *SLC6A4*).

*5-HTTLPR* is a repeat length polymorphism that has been shown to affect the efficiency of serotonin uptake and thus synaptic and extracellular serotonin concentrations in the brain. The short (s) allele of *5-HTTLPR* which shows a reduced transcriptional efficiency compared to the long (l) variant predisposes to cognitive vulnerability to stress-sensitivity including anxiety-related personality traits such as neuroticism [3], or amygdala reactivity to aversive stimuli [4] which are risk factors for major depressive disorder (MDD) [5,6]. Collier et al. [7] suggested that there is a significant, although weak, association between depression and *5-HTTLPR* s allele. Subsequently, primate studies demonstrated that the effect of the *5-HTTLPR* s allele on serotonin function in the central nervous system and on behavior is modulated by early rearing conditions providing the first evidence for GxE [8,9].

In the case of human depression, two main environmental psychosocial factors were identified, childhood adversity (CHA) and recent negative life events (RLE), which usually precede the development of episodes [1]. Caspi et al [10] reported that *5-HTTLPR* s allele modulates the effects of stressful life events in the development of depression. Although numerous genetic epidemiological studies replicated the initial findings, there are also non-replications and part replications, and even meta-analyses draw differing conclusions (see, e.g. [11,12]). The failure of genome-wide association studies (GWAS) to detect risk genes for MDD [13] further emphasize that depression as a diagnosis is genetically and phenotypically heterogeneous and delineation of more homogeneous specific sub-categories and inclusion of depression-related phenotypes are necessary to identify genetic risk factors [2,14,15]. To explore this concept, in our sufficiently large population we investigated depression-related phenotypes, such as lifetime depression, Brief Symptom Inventory current depression and anxiety scores to determine whether *5-HTTLPR* has similar effects on these measures and whether *5-HTTLPR* modulates the effects of life events in the development of these phenotypes. We specifically tested whether age and presence of CHA affects the modifying role of *5-HTTLPR* on the effect of RLE. To overcome the limitation of traditional (frequentist) statistical GxE analysis methods which have limited power to detect biological interactions [16], we calculated the Bayesian relevance of *5-HTTLPR* genotypes in specified models and the ratio of risk of these phenotypes conveyed by the ss genotype after low, medium and high exposure to life stressors.

## Methods

The reported studies were part of the EU funded NewMood study (New Molecules in Mood Disorders, Sixth Framework Program of the EU, LSHM-CT-2004–503474) approved by the local Ethics Committees (North Manchester Local Research Ethics Committee, Manchester, UK; Scientific and Research Ethics Committee of the Medical Research Council, Budapest, Hungary) and carried out in accordance with the Declaration of Helsinki. All participants provided written informed consent before participating in the study. All the relevant data are included in the paper and Supporting Information file (S2 File).

Subjects aged 18–60 years and of European white origin were recruited through general practices, advertisements and a web-site from Greater Manchester, UK (n = 1355) and through general practices and advertisements from Budapest, Hungary (n = 1003). All willing subjects were included who filled out the NewMood questionnaire pack, English or Hungarian version as appropriate, and provided DNA by using a genetic saliva sampling kit. Details of responses have been published previously [17,18].

The NewMood questionnaire included items covering background information (age, ethnicity, and family circumstances), personal and family psychiatric history and brief standard and validated questionnaires covering current mood and anxiety, and life events. A description of the questionnaires has been published previously [18,19].

In the present study, we analyzed reported lifetime depression (DEP) that was derived from the background questionnaire and was validated in a subpopulation using the Structured Clinical Interview for DSM-IV (SCID) [20]. Validation data were published recently [17]. Depressive symptoms were measured using the depression items plus the additional items (BSI-DEP), and anxiety using the anxiety items (BSI-ANX) of the Brief Symptom Inventory (BSI) [21]. A continuous weighted score (sum of item scores divided by the number of items completed) was calculated for each BSI variable mentioned above and used in the analysis. For Bayesian analysis the BSI depression and anxiety scores were divided into categorical variables (low, 0–<1; moderate, 1–<2; severe, 2–4).

We used the List of Life Threatening Experiences questionnaire [22] to identify recent negative life events (RLE) related to intimate relationships, financial difficulties, illnesses/injuries and network problems occurring in the last year. The number of life event items was calculated and used for the initial analysis. Next the scores were grouped into three categories (low = 0–1, medium = 2, high = 3 or more) based on our previous studies [18,19] and groups were used in the subsequent analysis and the Bayesian calculations. Childhood Adversity (CHA) questions related to emotional and physical abuse and emotional and physical neglect were derived from the Childhood Trauma Questionnaire [23] plus an additional question asked about parental loss during childhood. The CHA was validated with the full version of the Childhood Trauma Questionnaire in a subpopulation [17]. The sum of item scores was first calculated and used and next divided into three categories (low = 0–3, medium = 4–6, high = 7 or more) CHA based on our previous studies [17] for further statistical analysis.

For genotyping we used buccal mucosa cells collected using a cytology brush (Cytobrush plus C0012, Durbin PLC) and 2.0 ml of collection buffer in 15-mL plastic tubes. Genomic DNA was extracted according to a published protocol [24]. Determination of *5-HTTLPR* genotype was performed as previously described [18]. All laboratory work was performed under the ISO 9001:2000 quality management requirements and was blinded with regard to phenotype.

Statistical analyses were performed with PLINK v1.07 (http://pngu.mgh.harvard.edu/purcell/plink/) to calculate Hardy-Weinberg equilibrium, and build logistic regression models for binary outcome variable (DEP) and linear regression models for continuous outcome variables (BSI-DEP, BSI-ANX). Additive genetic model was applied, sex and age were covariates in all analyses. The two population data were analyzed together to increase the power, especially for GxE interaction tests. For power calculation, see Table A in S1 File. Because the *5-HTTLPR*xlife events (RLE or CHA) interaction results were very similar in the first analysis (see Table B in S1 File) all subsequent statistical calculation used life events as grouped variables to make it comparable to the Bayesian analysis. Main effects of *5-HTTLPR* and *5-HTTLPR*xRLE interaction were separately tested on each outcome variable in the total combined population and separately for subjects who were 30 years of age or below (≤30) and for those who were above 30 (>30). Other statistical analyses were performed with IBM SPSS 21.0 for Windows. We also used R Project [25] to support some of the PLINK analyses. All statistical testing used two-tailed p<0.05 threshold. Because all the calculations attempted to replicate previously published significant findings, results with nominal p<0.05 and concordant direction of effect was further investigated and reported, even if they would turn to non-significant after Bonferroni-correction for multiple testing.

To further characterize the nominally significant findings (either main genotype effects or the combined effects of GxE interactions) we applied Bayesian relevance analysis [26,27] based on Bayesian networks [28]. This method applies Bayesian statistics [29] to quantify the strong relevance of predictors with respect to a selected target as probability scores (posterior probability of relevance) and allows the detailed investigation of possible effect size of predictors (i.e. odds ratios). The method performs Bayesian model averaging [30,31], both at structural and parametric levels, thus handling the multiple hypothesis problem. This approach provides full Bayesian Odds Ratio measures for the effect size of a predictor, which is a more realistic measure than e.g. a single model-based confidence interval. To cope with heterogeneity of effects in various subpopulations, suggested by the scientific literature and our PLINK analysis, we performed separate analyses in subpopulations defined by the recent life event categories, childhood adversity categories and/or age (equal or <30 and >30 years of age), respectively. All odds ratios were estimated using the ll genotype of *5-HTTLPR* as a basis. (For details see Supporting Information in S1 File).

## Results

The total population and all sub-populations (Manchester and Budapest samples, and subjects with or without lifetime depression, respectively) were in Hardy-Weinberg equilibrium (p>0.05). The genotype frequency was not significantly different in the Budapest and Manchester samples (p_ADD_ = 0.154) which allowed us to carry out mega-analyses in the combined sample. Description of the population can be seen in Table 1. Increase of either RLE or CHA exposure positively correlated with BSI depression and anxiety scores, and caused sharp increase in risk of lifetime depression (Supporting Information and Fig. A in S1 File).

**Table 1:** Population description.

**Demographics**		
gender	male (%)	723 (31%)
	female (%)	1635 (69%)
lifetime depression (DEP)	no (%)	1380 (59%)
	yes (%)	978 (41%)
recent negative life events (RLE)	mean +/- SEM	1.21 (0.03)
	low (%)	1574 (67%)
	medium (%)	442 (19%)
	high (%)	338 (14%)
childhood adversity (CHA)	mean +/- SEM	3.29 (0.07)
	low (%)	1540 (65%)
	medium (%)	417 (18%)
	high (%)	388 (17%)
age	mean +/- SEM	32.79 +/- 0.22
	≤30 (%)	1083 (47%)
	>30 (%)	1203 (53%)
current depression score (BSI-DEP)	mean +/- SEM	0.85 (0.02)
	low (%)	1599 (68%)
	moderate (%)	414 (18%)
	severe (%)	341 (14%)
current anxiety score (BSI-ANX)	mean +/- SEM	0.88 (0.02)
	low (%)	1538 (65%)
	moderate (%)	472 (20%)
	severe (%)	344 (15%)
**Genetic variable**		
*5-HTTLPR*	ss (%)	438 (19%)
	sl (%)	1138 (48%)
	ll (%)	782 (33%)

SEM: standard error of mean; BSI: Brief Symptom Inventory.

### 5-HTTLPR effects on lifetime depression and BSI depression

**Fig 1:**
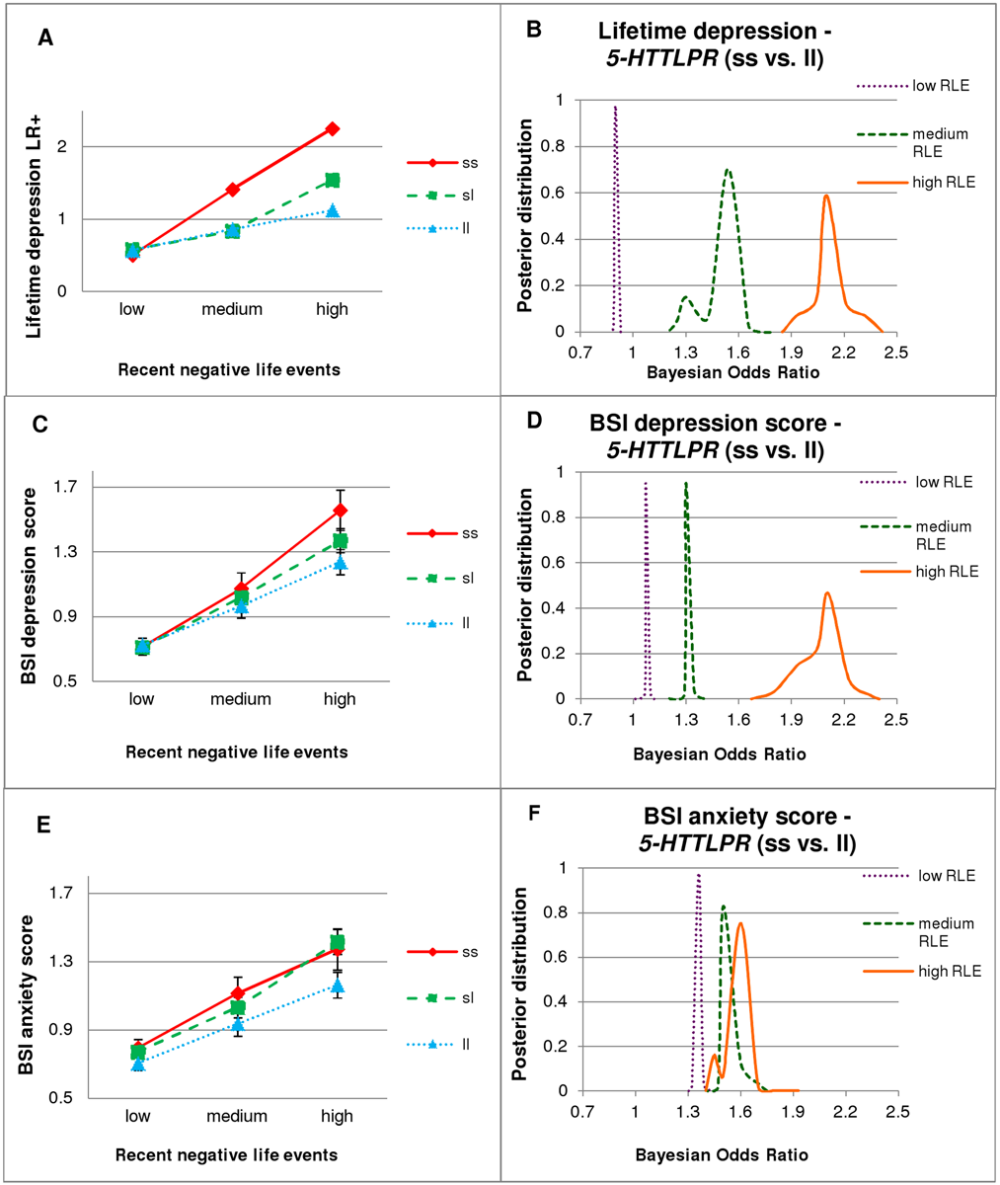
*5-HTTLPR*xRLE interaction, with PLINK (left column) and Bayesian (right column) analyses. LR+: likelihood ratio of emergence of the disease; BSI: Brief Symptom Inventory; BSI-DEP: BSI depression score; BSI-ANX: BSI anxiety score; DEP: lifetime depression; RLE: recent negative life events (in the last year). The three categories of RLE are: low = 0–1, medium = 2, high = 3 or more. Numbers in groups: low RLE: ss = 292, sl = 746, ll = 480; medium RLE: ss = 82, sl = 207, ll = 144; high RLE: ss = 51, sl = 146, ll = 134. Standard errors of means are displayed in case of continuous variables (left column). Right column figures display outlines of posterior distributions of Bayesian Odds Ratios of *5-HTTLPR* ss versus ll genotype with respect to DEP, BSI-DEP (severe vs. low), and BSI-ANX (severe vs. low). Subsets according to RLE categories (low, medium and high) were analyzed individually. Curve flatness refers to the number of possible models, each with a different odds ratio. An odds ratio greater than one represents a risk for the given phenotype. **1A.** Logistic regression analysis showed that having the more s alleles increased the risk of DEP with increasing number of RLE. **1B.** Regarding DEP there is a clear difference between subjects with low RLE (with a Bayesian Odds Ratio close to 1) and subjects with medium or high RLE (where the effect of ss genotype is stronger). **1C.** As in case of DEP: having the more s alleles also increased BSI-DEP with increasing number of RLE, using linear regression analysis. **1D.** As in case of DEP: effect of ss genotype on BSI-DEP is negligible in the low RLE group, but higher in the medium, and especially high in the high RLE group. **1E.** In contrast to depression phenotypes: linear regression analysis showed that carrying the more s alleles increased BSI-ANX without interaction with RLE. **1F.** In contrast to depression phenotypes, ss genotype represents a risk for BSI-ANX irrespective of RLE group.

*5-HTTLPR* effects on lifetime depression and BSI depression showed several similarities. Using regression analysis, there were no significant main effects of *5-HTTLPR* genotypes on reported lifetime depression or on BSI depression (Table 2). However, with the increasing number of s alleles, subjects proved to be more vulnerable to the depressogenic effect of the increasing number of RLEs (Table 2 and Fig. 1A and 1C, and Table E in S1 File). These interaction effects became non-significant in the two subpopulations split by age, possibly because of decreased power, although in all cases the s allele remained the risk one (Table 2 and Figs. B-A—B-D in S1 File). The significant interaction of *5-HTTLPR*xRLE was not due to the increased number of RLE in the s allele carriers. On the contrary, number of RLEs tended to decrease with the increasing number of s alleles (Table C in S1 File).

**Table 2:** Summary of the genetic association and interaction results using PLINK.

	Main effects of *5-HTTLPR*					*5-HTTLPR*xRLE					*5-HTTLPR*xRLE cov. BSI-ANX					*5-HTTLPR*xCHA				
**DEP**	OR	L95	U95	STAT	P (Pperm)	OR	L95	U95	STAT	P (Pperm)	OR	L95	U95	STAT	P	OR	L95	U95	STAT	P
**all**	1.037	0.920	1.168	0.589	0.556	**1.198**	**1.014**	**1.415**	**2.129**	**0.033 (0.037)**	*1*.*186*	*0*.*992*	*1*.*419*	*1*.*871*	*0*.*061*	1.017	0.864	1.197	0.203	0.839
**≤30**	1.019	0.852	1.219	0.208	0.835	*1*.*214*	*0*.*964*	*1*.*527*	*1*.*649*	*0*.*099*	1.194	0.934	1.526	1.413	0.158	1.075	0.835	1.385	0.561	0.575
**>30**	1.048	0.892	1.232	0.569	0.570	1.166	0.907	1.499	1.196	0.232	1.207	0.914	1.594	1.326	0.185	0.978	0.789	1.212	-0.205	0.838
**BSI-DEP**	BETA	SE		STAT	P	BETA	SE		STAT	P	BETA	SE		STAT	P	BETA	SE		STAT	P
**all**	0.011	0.027		0.413	0.680	**0.075**	**0.036**		**2.065**	**0.039 (0.036)**	*0*.*040*	*0*.*023*		*1*.*775*	*0*.*076*	0.013	0.033		0.377	0.706
**≤30**	0.025	0.039		0.639	0.523	0.078	0.049		1.587	0.113	0.035	0.032		1.081	0.280	0.016	0.052		0.315	0.753
**>30**	0.006	0.038		0.164	0.870	0.063	0.054		1.171	0.242	0.054	0.033		1.642	0.101	0.014	0.044		0.313	0.754
**BSI-ANX**	BETA	SE		STAT	P	BETA	SE		STAT	P						BETA	SE		STAT	P
**all**	**0.054**	**0.027**		**2.022**	**0.043 (0.042)**	0.043	0.035		1.220	0.223						0.005	0.033		0.156	0.876
**≤30**	**0.090**	**0.038**		**2.384**	**0.017 (0.019)**	0.055	0.047		1.162	0.246						0.032	0.050		0.631	0.528
**>30**	0.030	0.037		0.810	0.418	0.011	0.053		0.214	0.831						-0.005	0.044		-0.114	0.909

BSI: Brief Symptom Inventory; BSI-DEP: BSI depression score; BSI-ANX: BSI anxiety score; CHA: childhood adversity; DEP: lifetime depression; Pperm: permutated p values; RLE: recent negative life events (in the last year). Additive genetic models were calculated, where *5-HTTLPR* s allele represents the minor allele. Results are displayed in different groups: total population (all); those up to 30 years (≤30); and those above 30 years (>30). Regression equations (linear regression and beta for BSI-DEP and BSI-ANX scores, and logistic regression and odds ratio for DEP) always involve gender and age as covariates. In case of interaction models, main effect of the respective life event (RLE or CHA) was also covariate in the equation, besides its interaction with *5-HTTLPR*. And in case of the third model BSA-ANX was also a covariate. Permutated p values were calculated for the nominal p<0.05 results using PLINK—mperm 1000 for main 5HTTLPR effect on anxiety and using the “glmperm” R-package (http://cran.r-project.org/web/packages/glmperm/index.html, with 1000 permutations) for the interaction effects on DEP and BSI-DEP.

The three categories of RLE were: low = 0–1, medium = 2, high = 3 or more number of recent negative life events. The three categories of CHA were: low = 0–3, medium = 4–6, high = 7 or more scores.

Italics represent trends, and bold represents significant findings.

Bayesian effect strength estimation confirmed that the effect of *5-HTTLPR* on depression was relevant in the presence of RLEs. Namely, ss compared to ll genotype carriers have increased risk for lifetime depression and have higher BSI depression scores in subjects with medium (DEP: OR_Bayesian_ = 1.24–1.77; BSI-DEP: OR_Bayesian_ = 1.29–1.39) or high (DEP: OR_Bayesian_ = 1.85–2.4; BSI-DEP: OR_Bayesian_ = 1.67–2.4) RLE (Fig. 1B, 1D) but it has non-relevant effect (OR_Bayesian_≈1) in subjects with low RLE. The relatively flat posterior distribution curves in case of high RLE indicate several possible Bayesian Odds Ratio values with moderate or low certainty. Bimodal distribution for medium RLE subjects suggests that one group of models supports one, and another group of models supports another odds ratio.

There were no significant interaction effects of childhood adversity and *5-HTTLPR* genotypes on lifetime depression or on BSI depression scores (Table 2). However, *5-HTTLPR*xRLE interaction on BSI depression scores was significant in those who experienced medium or high childhood adversity. This remained significant at trend level in the younger age group but did not approach significance in the older group. In contrast, there was no *5-HTTLPR*xRLE interaction among subjects with either no childhood adversity or age above 30 (Table D in S1 File).

### 5-HTTLPR effects on BSI anxiety

Using linear regression analysis, BSI anxiety increased with the increasing number of s alleles (Table 2 and Fig. 1E, Table E in S1 File), irrespective of recent negative life events. This effect was significant in the younger (≤30) subpopulation but was not present in the elder (>30) subpopulation (Table 2, Figs. B-E and B-F in S1 File). However, there was no significant *5-HTTLPR*xRLE interaction on BSI-ANX (Table 2).

Bayesian Odds Ratio estimation implemented in the three (low, medium, high) RLE groups also support the *5-HTTLPR* main effect seen in PLINK results showing that the odds ratios are greater than one, irrespective of RLE group (Fig. 1F). The RLE credible intervals are not as separated as they are for depression phenotypes, where PLINK regression analyses detected interactional effects.

**Fig 2:**
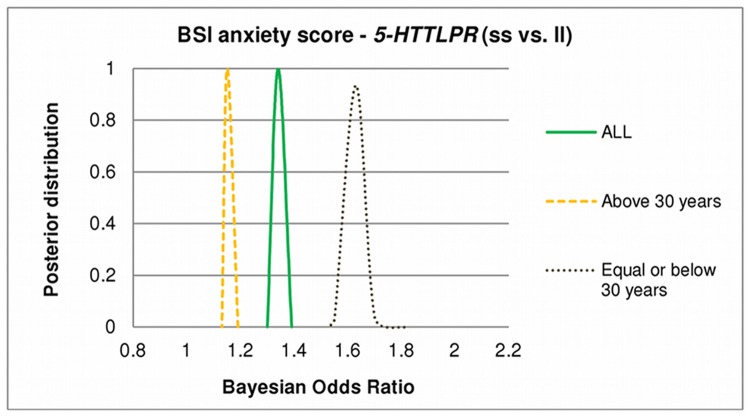
Effect of age on posterior distribution of Bayesian Odds Ratios of *5-HTTLPR* on BSI-ANX. BSI: Brief Symptom Inventory; BSI-ANX: BSI anxiety score. Curves display outlines of posterior distributions of Bayesian Odds Ratios of *5-HTTLPR* ss versus ll genotype with respect to BSI-ANX (severe vs. low). An odds ratio greater than 1 represents risk for BSI-ANX. Odds ratios are given in different age groups (all population; equal and below 30; and above 30). All curves are highly peaked that indicates that all the possible models entail highly similar odds ratios, and the Bayesian Odds Ratio values show a moderate *5-HTTLPR* ss genotype effect in the total population, strong effect in the younger subjects and negligible effects in the elder subpopulation.

In the severe vs. low anxiety comparison, Bayesian odds ratio estimation showed firm credible intervals for the ss vs. ll (OR_Bayesian_ = 1.31–1.39) effects, in the total population (Fig. 2). Results indicate a remarkable difference between the younger (≤30 years) and the elder (>30) subpopulations (supporting traditional PLINK results). In the elder subpopulation the effect of the ss compared to ll genotype of *5-HTTLPR* is modest (OR_Bayesian_ = 1.13–1.19), in the younger subpopulation, however, the effect is strong with a firm credible interval OR_Bayesian_ = 1.55–1.82 (Fig. 2).

There were no significant interaction effects of childhood adversity and *5-HTTLPR* genotypes on BSI anxiety scores and remained the non-significant *5-HTTLPR*xRLE interaction on BSI-ANX even in those who experienced medium or high childhood adversity (Table 2, and Table D in S1 File).

### The mediating role of BSI anxiety in the 5-HTTLPRxRLE interaction on depression

Next we tested whether the depressive effect of *5-HTTLPR* is mediated by the increased BSI anxiety scores related to the increasing number of s alleles. When we used BSI anxiety as a covariate in the regression models the significant *5-HTTLPR*xRLE interaction on lifetime depression and BSI depression scores became a trend in the total population (Table 2). Involving BSI anxiety, *5-HTTLPR*xRLE trend on lifetime depression also disappeared in the young subpopulation (Table 2). These results suggest that BSI anxiety partially mediates the depressive effect of *5-HTTLPR*xRLE interaction, especially in young people.

### 5-HTTLPR effects for the combined, multivariate phenotype in groups differentially exposed to RLE

**Fig 3:**
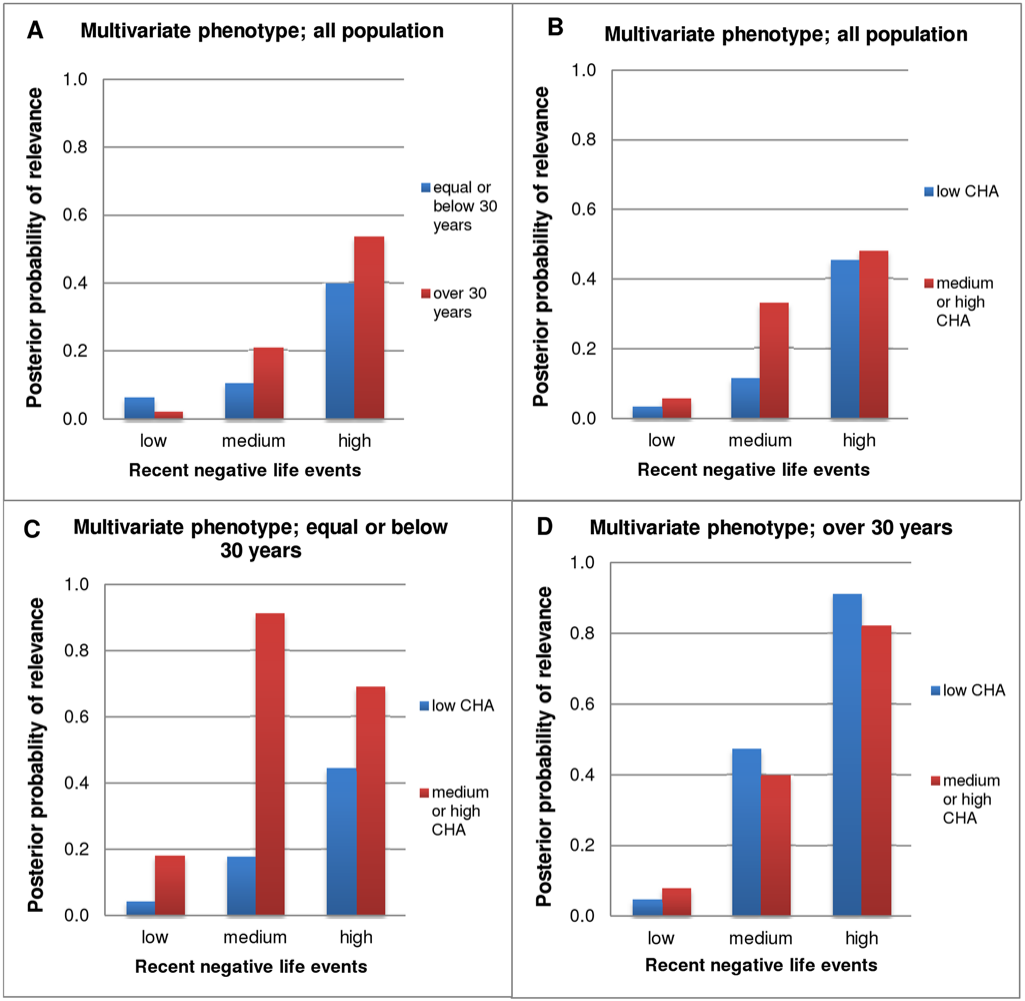
Bayesian posterior probabilities of relevance of *5-HTTLPR* for the multivariate phenotype. RLE was grouped into three categories: low = 0–1, medium = 2, high = 3 or more number of recent negative life events (in the past year). CHA (childhood adversity) was divided into two categories (based on the original three): low = 0–3, medium or high = 4 or more scores. Multivariate phenotype (more accurately describes depression related psychiatric state than one phenotype measure alone) encompasses lifetime depression, BSI depression score and BSI anxiety score. Results are displayed according to CHA and age, in groups differentially exposed to RLE. **3A and 3B.** Results demonstrate moderate Bayesian probability of relevance of *5-HTTLPR* in both age groups and CHA groups in those who had 3 or more RLE. **3C.** In the younger age group (≤30) *5-HTTLPR* was strongly relevant in those who had medium or high CHA and even moderate number of RLE. **3D.** In the older age group (>30) *5-HTTLPR* was strongly relevant in those who had 3 or more RLE, irrespective of CHA.

Finally we calculated Bayesian probability of relevance for the combined, multivariate phenotype using all three measured phenotypes: lifetime depression, BSI depression score, and BSI anxiety score. Our results demonstrated moderate probability of relevance (Pr = 0.3–0.5) of *5-HTTLPR* in both age groups (Fig. 3A) and childhood adversity groups (Fig. 3B), in those who had high recent negative life events. In addition, in the younger age group (≤30) *5-HTTLPR* was strongly relevant in those who had medium or high childhood adversity and even moderate number of recent negative life events (Pr = 0.91 and Pr = 0.69 in moderate and high RLE groups, respectively; Fig. 3C), while in the older age group (>30) *5-HTTLPR* was strongly relevant only in those who had high recent negative life events but this effect was present irrespective of childhood adversity (Pr = 0.91 and Pr = 0.82 in low and medium-high CHA groups, respectively; Fig. 3D).

## Discussion

According to our results, exposure either to RLE or CHA caused a marked, dose-dependent increase in all three depression-related phenotypes (Supporting Information and Fig. A in S1 File). However, *5-HTTLPR* weakly modulated the effect of RLE on lifetime depression and current BSI-depression, while in BSI-anxiety it showed a weak main effect, namely a direct association with the risk s allele even in individuals having experienced low life stress, and stress exposure caused only a minor additional increase in anxiety. These weak genetic associations are in line with the hypothesis that depression and anxiety are highly polygenic and multifactorial disorders [2,13]. This is confirmed also by the GxG interaction effect of 5-HTTLPR with CB1 receptor gene promoter polymorphisms in the anxiety phenotype, and supported also by animal experiments in anxiety models [32,33]. The direct association of anxiety with the s allele was stronger in young (≤30) individuals. In addition, although CHA did not show any interaction with *5-HTTLPR* on any of the phenotypes, it had an important influence on the *5-HTTLPR*xRLE interaction. Namely, in young subjects it sensitized towards the effect of RLE even if RLE was mild, when a combined multivariate outcome was used. In older subjects (>30) *5-HTTLPR* was only relevant when more RLE were reported irrespective of childhood adversity. The results suggest that the modulatory effects of serotonin transporter gene variation on the risk of depression may involve different mechanisms in different age groups.

### Pleiotropic effect of 5-HTTLPR on depression-related phenotypes

In keeping with previous findings we found genetic effects of *5-HTTLPR* in both anxiety and depression in the predicted direction, namely s allele being a risk variant (Table E in S1 File). What is more intriguing, is that we replicated a direct association between *5-HTTLPR* and anxiety [3,34,35] and a *5-HTTLPR*xRLE interaction in lifetime and current BSI-depression [10,18] in a large non-clinical population sample. In addition, we found that anxiety partially mediates the effect of the *5-HTTLPR*xRLE interaction on the emergence of depression, as was previously hypothesized [36]. These findings may shed new light on the complex and multilevel relationship between different manifestations of increased vulnerability related to *5-HTTLPR* as well as on the relationship between anxious and depressive symptomatology and morbidity. Clinically there is an extensive correlation between anxiety and depression, reflected not only in the frequent comorbidity between the two conditions but also in the clinical entity of mixed anxiety and depression in ICD-10, and the shared genetic risk factors between these two phenomena [2,14]. Our results suggest that *5-HTTLPR* is one of the shared genetic factors. However, the fact that its effects on depression act through anxiety and life stress suggests multiple central nervous system actions. Important human neuroimaging findings demonstrated that *5-HTTLPR* s allele carriers have greater “tonic” amygdala activation at rest [5], which is in line with our finding of a direct association between *5-HTTLPR* and anxiety. Our GxE findings for depression suggest that in addition to tonic, a phasic effect of *5-HTTLPR* on emotion processing also exists. Indeed, other brain imaging studies showed increased amygdala response to negative emotional stimuli [4], and indicated an association between the s allele and enhanced acute stress reactivity in a broader brain network [37].

### Stress sensitizing effect of CHA and the effect of 5-HTTLPR

Interestingly, and in contrast to previous results, we could only demonstrate an effect of recent life events in our interaction models, but not of childhood maltreatment [10,11,14,38]. As a possible explanation for the discrepancies concerning *5-HTTLPR* and different types of life events it has been suggested that the role of childhood adversity may be more specific to recurrent or chronic depression than to single depressive episode occurrences [38,39]. This is in line with the stress sensitization hypothesis, which suggests that childhood adversity leads to increased vulnerability to adult stressors and thus more psychopathology, especially increased risk for depression [40]. In our study, childhood adversity sensitized young subjects (≤30) to recent negative life events, such that even in the case of medium number of RLE *5-HTTLPR* showed high relevance in the depression-related multivariate phenotype. However, we could not demonstrate a similar sensitization effect in the older group (>30).

### Age and the effect of 5-HTTLPR

The GxE interaction between *5-HTTLPR* genotype and environmental adversities on the development of depression appears to be a function of several other variable conditions, and also seems to be an age dependent effect as it is reported as a significant finding in studies in young adults but not in adolescents or the elderly [14]. Based on our results, *5-HTTLPR* is strongly relevant in older adults (>30) only in the presence of high number of RLE suggesting that tonic effect of this polymorphism might weaken with age but robust stress could still elicit phasic effects. As the incidence of depression is also age-dependent, it is not unlikely that in different ages and stages of development different factors play a role in the development of depression, and thus this risk is also influenced by different genetic factors.

### Limitations

Besides advantages, there are several limitations of our study to be noted. In our cross-sectional population sample almost half of the willing participants reported lifetime depression that might introduce sampling bias. Current (BSI) or lifetime depression and current BSI-anxiety were assessed via self-report, with no psychiatric screening that might result inaccurate phenotype calling. However, our data were validated [17,19] in a subsample during face-to-face interviews using the Structured Clinical Interview for DSM-IV (SCID, [20]), the interviewer rated Montgomery Asberg Depression Rating Scale (MADRS, [41]) and the Clinical Anxiety Scale (CAS, [42]). Assessment of recent negative life events and childhood adversities was also based on self-report but again used validated questionnaires as described in the methods section. Our study was cross-sectional thus no longitudinal effects of life stresses were available and therefore persistence of depression and the timing of life events relative to depression onset could not be determined. And finally, because of the limited power further potentially important factors such as social support were not included in the analysis.

### Implication for future studies

Based on our results the effect of the *5-HTTLPR* might be better captured in specific subgroups of the population than in large but heterogeneous population samples. Our Bayesian Odds Ratio calculations demonstrated that in these subgroups the genetic ORs are increased. Such stratified analyses should result in greater power in future GxE interaction studies (see Table A in S1 File). In addition, our study demonstrated that the Bayesian network-based methodology, which was developed to analyze relevance of predictors with respect to a set of phenotypic, clinical and environmental descriptors, is a powerful approach to identify highly relevant genetic risk factors even when traditional (frequentist) analysis methods are not able to provide significant results.

## Conclusions

In conclusion, our results emphasize that genetic variation in *5-HTTLPR* is relevant to the development of depressive symptomatology. However its effects are expressed through a multilevel network of interactions among genes, and between genes and the environment. No doubt there are several other important modifying variables still to be discovered [43,44,45]. Our results also indicate how methodological differences between studies may obscure or mask important associations and emphasize the need for further studies applying sophisticated designs and alternative mathematical methods to clarify and deepen our understanding of the role of genetic risk factors for depression and anxiety.

## Supporting Information

S1 File Contents. Power calculation (description)Table A. Required sample sizes for 90% powerTable B. Similar effects of continuous and grouped life event scores in the PLINK analysisTable C. Effect of *5-HTTLPR* on RLE or CHATable D. Effect of age and childhood adversity on *5-HTTLPR*xRLE interactionFig. A. Likelihood ratio of lifetime depression according to the life event groupsEffects of life events (description)Fig. B. Interaction between *5-HTTLPR* and RLE on different phenotypes, in two age subgroupsBayesian analysis of relevance (description)Bayesian Odds Ratio (description)The analysis of the joint effect of *5-HTTLPR* and RLE (description)Table E. Comparison of logistic regression models of *5-HTTLPR* (ss vs. ll) with respect to phenotypes BSI-ANX, BSI-DEP and DEP
(PDF)

S2 File Contents. Data
(PDF)
