# Supplementary material for: Variability in the Effect of 5-HTTLPR on Depression in a Large European Population: The Role of Age, Symptom Profile, Type and Intensity of Life Stressors
Source: PLoS One. 2015 Mar 6;10(3):e0116316. doi: 10.1371/journal.pone.0116316 (PMC4351953; doi:10.1371/journal.pone.0116316)
Supplement: S1 File — Power calculation (description)Table A. Required sample sizes for 90% powerTable B. Similar effects of continuous and grouped life event scores in the PLINK analysisTable C. Effect of 5-HTTLPR on RLE or CHATable D. Effect of age and childhood adversity on 5-HTTLPRxRLE interactionFig. A. Likelihood ratio of lifetime depression according to the life event groupsEffects of life events (description)Fig. B. Interaction between 5-HTTLPR and RLE on different phenotypes, in two age subgroupsBayesian analysis of relevance (description)Bayesian Odds Ratio (description)The analysis of the joint effect of 5-HTTLPR and RLE (description)Table E. Comparison of logistic regression models of 5-HTTLPR (ss vs. ll) with respect to phenotypes BSI-ANX, BSI-DEP and DEP (PDF) [file pone.0116316.s001.pdf]

Variability in the effect of 5-HTTLPR on depression in a large European population: the role of age, symptom profile, type and intensity of life stressors

Juhasz et al.

Correspondence to: Gabriella Juhasz: Department of Pharmacodynamics, Faculty of Pharmacy, Semmelweis University, Budapest, Hungary, Postal address: 1089 Budapest, Nagyvarad ter 4. Hungary. Phone: +36-1-4591500/56362, Fax: +36-1-4591494, E-mail: gabriella.juhasz@manchester.ac.uk,

Keywords: serotonin transporter, *5-HTTLPR*, gene-by-environment interaction, depression, anxiety

Supporting information

Contents of the file:

- **Power calculation (description)**
- **Table A. Required sample sizes for 90% power**
- **Table B. Similar effects of continuous and grouped life event scores in the PLINK analysis**
- **Table C. Effect of *5-HTTLPR* on RLE or CHA**
- **Table D. Effect of age and childhood adversity on *5-HTTLPR*xRLE interaction**
- **Figure A. Likelihood ratio of lifetime depression according to the life event groups**
- **Effects of life events (description)**
- **Figure B. Interaction between *5-HTTLPR* and RLE on different phenotypes, in two age subgroups**
- **Bayesian analysis of relevance (description)**
- **Bayesian Odds Ratio (description)**
- **The analysis of the joint effect of *5-HTTLPR* and RLE (description)**
- **Table E. Comparison of logistic regression models of *5-HTTLPR* (ss vs. ll) with respect to phenotypes BSI-ANX, BSI-DEP and DEP**

Power calculation

Quanto 1.2.4 version (http://hydra.usc.edu/gxe) was employed to calculate the power of the present study.

In the total population (n=2358, with a minor allele frequency 40%) we have higher than 90% power to detect genetic effects that explain 0.5% of variations in a continuous variable, or are associated with an OR 1.15 for a disease. We also have higher than 90% power to detect a gene x environment interaction (40% of the population reported environmental stressors) that explain 0.5% of variations in a continuous variable, or are associated with 1.3 odds ratio for a disease.

In the subpopulations according to age groups ($n_{\leq 30}$=1082 and $n_{>30}$=1200, with a minor allele frequency 40%) we have higher than 90% power to detect genetic effects that explain 1% of variations in a continuous variable, or are associated with an OR 1.2 for a disease. We also have higher than 90% power to detect a gene x environment interaction (40% of the population reported environmental stressors) that explain 1% of variations in a continuous variable, or are associated with 1.5 odds ratio for a disease.

In **Table A** required sample sizes are displayed at different exposure levels to recent negative life events (RLE), with respect to varying genetic relative risk. Ideal sample size decreases as a function of both the increasing RLE and the increasing relative risk. A CGAS study, focusing only on effects of candidate genes, always requires a smaller sample than a GWAS study with the same exposure level and genetic relative risk, to gain the same power.

Table A. Required sample sizes for 90% power.

Environmental exposure	Genetic relative risk	Sample size	
		CGAS (p=0.05)	GWAS ($p=5x10^{-8}$)
0-1 (low) RLE	1.05	35978	155214
	1.1	9426	40664
2 or more RLE	1.2	2588	11162
	1.3	1256	5420
3 or more (high) RLE	1.4	774	3342
	1.5	538	2324
	1.6	406	1748
	1.7	322	1386
	1.8	264	1142
	1.9	224	970
	2	196	842

RLE: recent negative life events (in the last year); CGAS: candidate gene association study (effect detected at a 0.05 significance level); GWAS: genome-wide association study (effect detected at a $5x10^{-8}$ significance level). Genetic relative risk refers to the odds ratio of major depressive disorder given the risk allele. CGAS and GWAS studies need different significance levels (related to Type I errors) to gain the same power.

Table B. Similar effects of continuous and grouped life event scores in the PLINK analysis.

	DEP					BSI-DEP				BSI-ANX			
	OR	L95	U95	STAT	P	BETA	SE	STAT	P	BETA	SE	STAT	P
**main effects**	1.037	0.920	1.168	0.589	0.556	0.011	0.027	0.413	0.680	**0.054**	**0.027**	**2.022**	**0.043**
**RLE (cont)**	**1.106**	**1.002**	**1.221**	**1.991**	**0.046**	*0.040*	*0.021*	*1.919*	*0.055*	*0.034*	*0.020*	*1.708*	*0.088*
**RLE (3 groups)**	**1.198**	**1.014**	**1.415**	**2.129**	**0.033**	**0.075**	**0.036**	**2.065**	**0.039**	0.043	0.035	1.220	0.223
**CHA (cont)**	0.995	0.956	1.035	-0.271	0.786	0.005	0.007	0.699	0.485	0.001	0.007	0.145	0.885
**CHA (3 groups)**	1.017	0.864	1.197	0.203	0.839	0.013	0.033	0.377	0.706	0.005	0.033	0.156	0.876

BSI: Brief Symptom Inventory; BSI-DEP: BSI depression score; BSI-ANX: BSI anxiety score; CHA: childhood adversity; DEP: lifetime depression; RLE: recent negative life events (in the last year); cont: continuous.

Additive genetic models were calculated, where *5-HTTLPR* s allele represents minor allele. Regression equations (linear regression and beta for BSI-DEP and BSI-ANX scores, and logistic regression and odds ratio for DEP) always involve sex and age as covariates. In case of interaction models, main effect of the respective life event (RLE or CHA) was also covariate in the equation, besides its interaction with *5-HTTLPR*.
The three categories of RLE were: low=0-1, medium=2, high=3 or more number of recent negative life events. The three categories of CHA were: low=0-3, medium=4-6, high=7 or more scores.
Italics represent trends, and bold represents significant findings.

Table C. Effect of *5-HTTLPR* on RLE or CHA.

	**Main effect of *5-HTTLPR***			
	BETA	SE	STAT	P
**RLE**	*-0.067*	*0.038*	*-1.755*	*0.079*
**CHA**	0.035	0.099	0.350	0.726

CHA: childhood adversity; RLE: recent negative life events (in the last year). Both were used as continuous variables without grouping.
Italics represent a trend to report less RLEs with the increasing number of s alleles (in an additive model). Linear regression equations involve gender and age as covariates.

Table D. Effect of age and childhood adversity on *5-HTTLPR*xRLE interaction.

*5-HTTLPR*xRLE	**Low CHA score (0-3)**					**Medium or high CHA score (4 or more)**				
**DEP**	OR	L95	U95	STAT	P	OR	L95	U95	STAT	P
**all**	1.139	0.917	1.416	1.174	0.240	1.234	0.928	1.640	1.446	0.148
**≤30**	1.082	0.800	1.462	0.510	0.610	*1.431*	*0.960*	*2.135*	*1.758*	*0.079*
**>30**	1.234	0.888	1.713	1.252	0.211	0.965	0.630	1.478	-0.164	0.870
**BSI-DEP**	BETA	SE		STAT	P	BETA	SE		STAT	P
**all**	0.005	0.040		0.126	0.900	**0.141**	**0.065**		**2.186**	**0.029**
**≤30**	0.023	0.057		0.415	0.678	*0.163*	*0.089*		*1.840*	*0.067*
**>30**	-0.028	0.058		-0.489	0.625	0.118	0.094		1.247	0.213
**BSI-ANX**	BETA	SE		STAT	P	BETA	SE		STAT	P
**all**	0.023	0.040		0.583	0.560	0.038	0.063		0.598	0.550
**≤30**	0.028	0.055		0.501	0.616	0.086	0.085		1.012	0.312
**>30**	0.004	0.059		0.062	0.951	-0.034	0.093		-0.366	0.715

BSI: Brief Symptom Inventory; BSI-DEP: BSI depression score; BSI-ANX: BSI anxiety score; CHA: childhood adversity; DEP: lifetime depression; RLE: recent negative life events (in the last year).

Additive genetic models were calculated, where *5-HTTLPR* s allele represents minor allele. Results are displayed in different groups for both CHA subsets: total population (all); those up to 30 years (≤30); and those above 30 years (>30). Regression equations (linear regression and beta for BSI-DEP and BSI-ANX scores, and logistic regression and odds ratio for DEP) always involve gender, age and main effect of RLE as covariates. The three categories of RLE were: low=0-1, medium=2, high=3 or more number of recent negative life events. Interactional effect of *5-HTTLPR*xRLE showed significance neither in low CHA groups, nor in elder groups, on any of the phenotypes. However, the same interactional effect became a trend (displayed in italics) in those who are equal or below 30 years and had medium or high CHA, but only on the two depression phenotypes. These results suggest a gene x environment x environment x age interaction on depression.
*5-HTTLPR*xRLE interaction on BSI-DEP is significant (displayed in bold) in the group with medium or high CHA but without age split.

Figure A. Likelihood ratio of lifetime depression according to the life event groups.

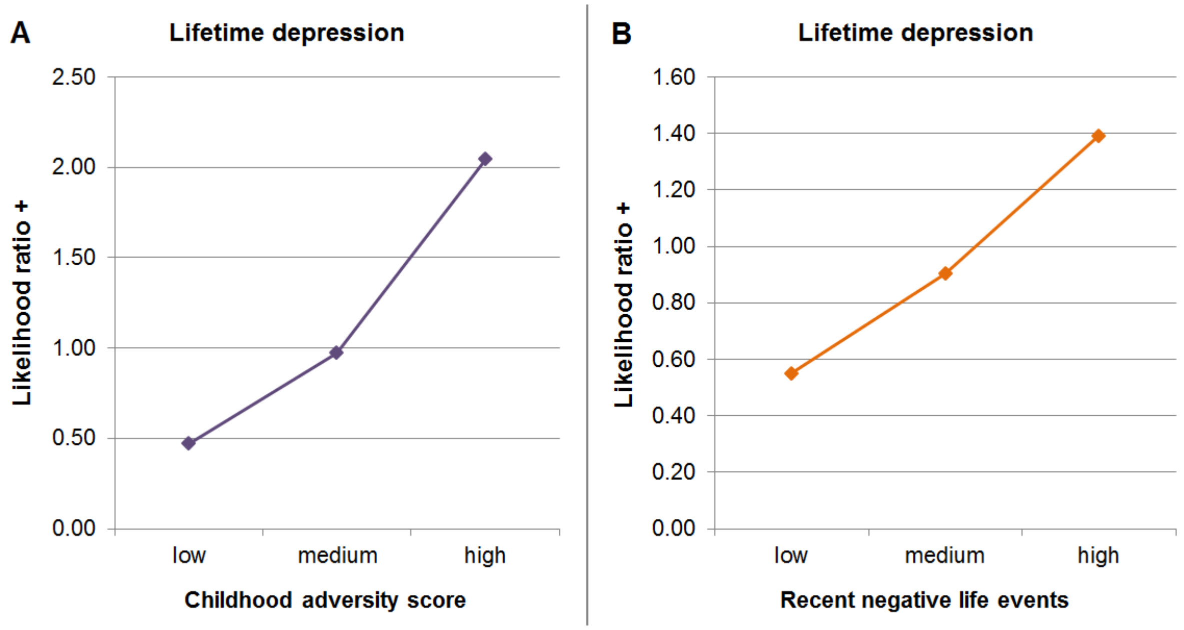

Likelihood ratio of emergence of lifetime depression increases with increasing presence of either type of negative life events: **A.** Childhood adversity; **B.** Recent negative life events (in the last year).

Effects of life events

As it was expected, both childhood adversity and recent negative life events increased the risk of reported lifetime depression (CHA: Pearson Chi-Square=173.54 df=2 $p<0.001$; RLE: Pearson Chi-Square=70.06 df=2 $p<0.001$; see also **Figure S1**), and resulted in significantly higher BSI depression (CHA: Pearson F=145.83 df=2,2338 $p<0.001$; RLE: F=80.78 df=2,2347 $p<0.001$) and anxiety (CHA: Pearson F=94.57 df=2,2338 $p<0.001$; RLE: F=67.72 df=2,2347 $p<0.001$) scores.

Figure B. Interaction between *5-HTTLPR* and RLE on different phenotypes, in two age subgroups.

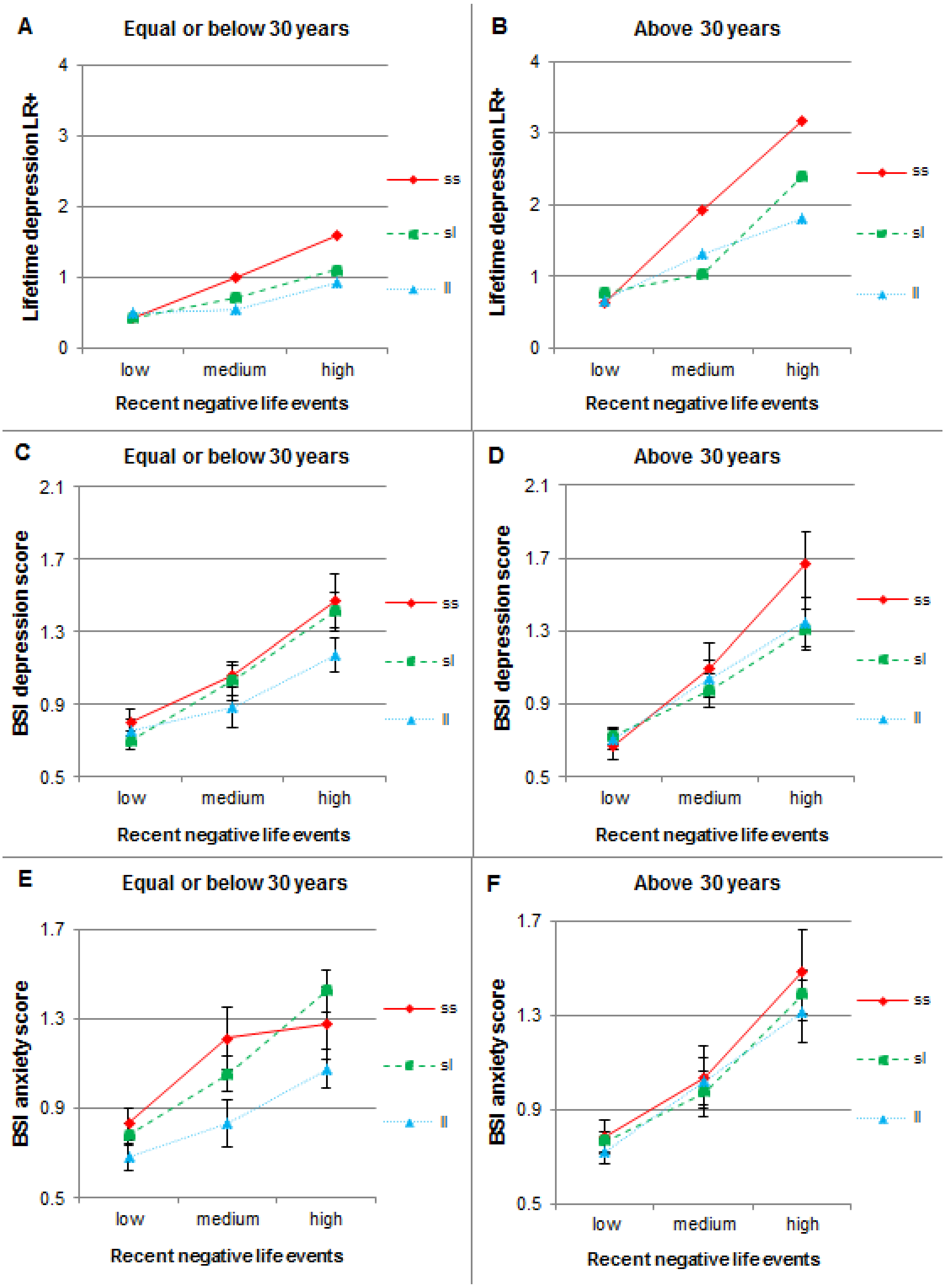

Standard errors of means are displayed in case of continuous variables. BSI: Brief Symptom Inventory; LR+: likelihood ratio of emergence of the disease; RLE: recent negative life events (in the last year).

Numbers in groups, equal or below 30 years: low RLE: ss=135, sl=330, ll=203; medium RLE: ss=38, sl=120, ll=63; high RLE: ss=26, sl=78, ll=89.

Numbers in groups, above 30 years: low RLE: ss=157, sl=416, ll=277; medium RLE: ss=44, sl=87, ll=81; high RLE: ss=25, sl=68, ll=45.

Interactional effect of *5-HTTLPR* genotype (in an additive model) and RLE on lifetime depression proved to be a trend among young (**Figure B-A**), but not among elder persons (**B-B**).
Interactional effect of *5-HTTLPR* genotype (in an additive model) and RLE on BSI depression score could not be reproduced in any of the age subgroups (**B-C and B-D**).
Significant main effect of *5-HTTLPR* s allele (in an additive model) on BSI anxiety score could only be seen in the young subgroup (**B-E**), but not in those above 30 years (**B-F**).

Bayesian analysis of relevance

The Bayesian network-based Bayesian multilevel analysis of relevance (BN-BMLA) methodology was developed to enable the analysis of the relevance of predictors with respect to a set of phenotypic, clinical and environmental descriptors.

It is based on a subclass of probabilistic graphical models, the Bayesian networks (BN) which provide a unifying framework for systems-based modeling and data analysis [1]. Bayesian network based frameworks gained popularity in genetics, particularly for the analysis of genetic association studies (GAS) due to their systems-based foundation [2,3].

An additional challenge of genetic association studies with complex phenotypes is the severe presence of the multiple hypothesis testing problem. BN-BMLA was proposed as a systems-based association analysis methodology using the Bayesian statistical framework, which allows optimal correction to cope with this challenge [4,5]. It also provides a principled foundation for multivariate association and interaction analysis. Furthermore, it also allows the explicit modeling of the interdependency structure of the target (or outcome) variables.

A Bayesian network model $BN(G, \theta)$ consists of a directed acyclic graph structure G, and a parameterization θ. The former represents the multivariate dependency relationships of variables $\boldsymbol{V}$ (factors), whereas the latter quantitatively defines the dependency relationships by conditional probability distributions.

The *a posteriori* probability (posterior) of a multivariate dependency model (i.e. a BN) is defined by the Bayes rule:

$$P(G,\theta \mid D) \propto P(D \mid G,\theta) P(G,\theta),$$

which is the product of marginal likelihood $P(D|G,\theta)$ of the data D given structure G and its parameters θ, and the prior probability $P(G,\theta)$. The likelihood is a probability measure indicating the probability that the data was generated by a given BN, and the prior $P(G,\theta)$ encodes prior knowledge on possible structures and their parameterizations as structure and parameter priors respectively. We used BDeu parameter priors with virtual sample size 1 and a uniform prior over structures with a restricted number of possible incoming arcs.

Basically, BN-BMLA aims to discover strongly relevant factors X_i with respect to a target Y. This concept of strong relevance is closely related to the Markov blanket sets of Y, which can be defined as a structural property of a BN. More specifically, a factor X is strongly relevant if it is a member of the Markov blanket set of Y. Let $I_{SR}(X,Y,G)$ be an indicator function of X being in the Markov blanket of Y. Since there are several possible models G, the probability of strong relevance of factor X with respect to target Y denoted as $P(SR(X,Y))$ can be defined by model averaging [6,7] as

$$P(SR(X,Y) \mid D) = \sum_{G} P(G \mid D) I_{SR}(X,Y,G)$$

In fact, the number of possible structures is intractable even for a moderate number of variables, therefore the posterior probability $P(SR(X,Y))$ has to be estimated by some approximation method. BN-BMLA facilitates this by performing a random walk in the space of BN structures G and using a Markov Chain Monte Carlo (MCMC) sampling method, which inserts, deletes, and inverts edges [8]. The probability of applying different operators in the proposal distribution is uniform, the length of the burn-in is 10^6 and the length of the sample collection is $5\text{x}10^6$.

The convergence of the MCMC simulation for the estimated posteriors was validated by Geweke Z-scores and the Gelman-Rubin R-scores (less than 0.1 and 1.1, respectively) and confidence intervals (less than 0.1) [9].

Bayesian Odds Ratio

The Bayesian Odds Ratio is an extension to BN-BMLA which applies a hybrid approach towards effect size estimation by combining parametric relevance and strong relevance [10]. The estimation of Bayesian Odds Ratios is performed based on the same Bayesian framework that allows the structural exploration of associations.

This can be regarded as a "full Bayesian" approach, because the parameter level uncertainty is combined with the structure level uncertainty using Bayesian model averaging [6,7] as follows:

$$p(OR \mid D) = \sum_{G} \int_{\theta} p(OR \mid \theta, G, D) P(\theta \mid G, D) P(G \mid D) d\theta,$$

in which $p(OR|\theta,G,D)$ is the posterior distribution of the odds ratio given data D and a possible graph structure G and parameterization θ; $p(\theta\ |G,D)$ is the probability of the parameterization given G, whereas $p(G|D)$ is the probability of that graph structure given data D. The Bayesian Odds Ratio $p(OR|D)$ is a weighted average of posteriors $p(OR|\ \theta, G,D)$.

However, even in case of moderate number of variables, the number of possible structures is intractable, i.e. the summing over $\boldsymbol{G}$ is computationally unfeasible. A possible solution is to consider only Markov blanket graphs which do not contain all the dependency relationships of variables just those in which the target Y is involved.

Since the aim of effect size measures is to quantify the effect of a factor on the target Y, this is a useful property. Therefore, we devised a Bayesian structure conditional effect size measure, which applies Markov blanket graphs to estimate the effect size distribution of variable X_i (with respect to a specific target Y). For each Markov blanket graph MBG_Y^j an odds ratio is estimated $p(OR|MBG_Y^j,D)$ and weighted by the corresponding posterior probability $p(MBG_Y^j|D)$ of MBG_Y^j given data D

$$p(OR \mid D) \sim \sum_{MBG_Y} p(OR \mid MBG_Y, D) P(MBG_Y \mid D),$$

If X_i is not a member of an MBG_Y^j, then X_i is not relevant with respect to the target Y (i.e. X_i has no relevant effect on Y) in that particular model. Such an MBG_Y^j can be treated as a case with neutral effect size ($OR=1$), or can be excluded from the averaging. In this paper we only used those MBG_Y^j for effect size estimation in which X_i was a member and X_i had a direct connection to the target Y.

The resulting Bayesian Odds Ratio $p(OR|D)$ is a posterior distribution over odds ratios, which provides a finer characterization of effect size and allows a more detailed analysis than a conventional confidence interval. The Bayesian analogue of a confidence interval is called credible interval, which is computed based on the 95% HPD (high probability density) region of the Bayesian Odds Ratio.

The analysis of the joint effect of *5-HTTLPR* and RLE

Figures 1B and **1D** illustrate the joint effect of *5-HTTLPR* and RLE for the DEP and BSI-DEP phenotypes. It is shown that the effect size of *5-HTTLPR* remarkably differs between various RLE categories. The variance of the odds ratio distribution of each RLE category reflects the corresponding sample size, i.e. smaller sample size induces a wider credible interval.

However, in case of the BSI-ANX phenotype this difference is less pronounced (see **Figure 1F**). On the other hand, by comparing these results to the effect size observed on the whole population (see **Figure 2. 'ALL'**) an interesting phenomenon can be seen. Namely that all peak values of the Bayesian Odds Ratio curves related to the various RLE subpopulations (RLE 0 or 1: $OR_{Bayesian}$= 1.36, RLE 2: $OR_{Bayesian}$= 1.5, RLE 3 or more: $OR_{Bayesian}$= 1.6) are greater than that of the whole population ($OR_{Bayesian}$= 1.34). To investigate this phenomenon we applied logistic regression to model the effect of *5-HTTLPR* with and without the RLE interaction. **Table S5** shows two logistic regression models consisting of *5-HTTLPR*, RLE, and AGE for each of the three phenotypes. Only one of the models includes a *5-HTTLPR*xRLE interaction term. Logistic regression coefficients (*Exp(B)*) can be interpreted as effect sizes related to a specific model.

For the DEP phenotype the observed effect size of *5-HTTLPR* is low in case of a simple logistic regression model (Exp(B)= 1.137), and *5-HTTLPR* is not significant (p-value = 0.306) with respect to the model. On the other hand, the logistic regression model containing a *5-HTTLPR*xRLE interaction term produces a higher effect size for the interaction (Exp(B)= 1.483) which is moderately significant (p-value= 0.028), and a lower effect size for the main effect of *5-HTTLPR* (Exp(B)= 0.945). This confirms the importance of taking *5-HTTLPR*xRLE interaction into consideration when exploring the effects of *5-HTTLPR.*

In contrast, in case of the BSI-ANX phenotype the interaction term does not improve the model. In the simple logistic regression model *5-HTTLPR* has a relatively high effect size (Exp(B)= 1.499), which is moderately significant with respect to the model (p-value=0.025). However, the addition of the interaction term results in a lower effect size for both the main effect and the interaction (Exp(B)=1.426 and Exp(B)=1.085 respectively).

These results indicate that *5-HTTLPR* affects BSI-ANX through a different mechanism than it affects DEP, as the former required a simple model, whereas the latter required a model with an interaction term. For the potential intermediate role of anxiety in depression see the discussion section.
The observed phenomenon (described above) is the consequence of the non-linear nature of odds ratios, which can be thus modeled by the multivariate capability of logistic regression.

Table E. Comparison of logistic regression models of *5-HTTLPR* (ss vs. ll) with respect to phenotypes BSI-ANX, BSI-DEP and DEP.

Phenotype	Model	p-value	Exp(B)	95% C.I. for EXP(B)	
				Lower	Upper
DEP	RLE	<0.0001	1.690	1.441	1.981
	5-HTTLPR	.306	1.137	.889	1.455
	AGE	<0.0001	1.271	1.134	1.424
	RLE	<0.0001	1.501	1.242	1.814
	5-HTTLPR	.707	.945	.702	1.271
	AGE	<0.0001	1.264	1.128	1.417
	*5-HTTLPR*xRLE	.028	1.483	1.045	2.106
BSI-DEP*	RLE	<0.0001	2.028	1.649	2.494
	5-HTTLPR	.129	1.304	.926	1.836
	AGE	.303	1.088	.927	1.278
	RLE	<0.0001	1.851	1.437	2.386
	5-HTTLPR	.659	1.104	.713	1.709
	AGE	.339	1.082	.921	1.271
	*5-HTTLPR*xRLE	.224	1.309	.848	2.019
BSI-ANX*	RLE	<0.0001	2.051	1.644	2.558
	5-HTTLPR	.025	1.499	1.051	2.137
	AGE	.033	1.198	1.015	1.416
	RLE	<0.0001	1.993	1.517	2.619
	5-HTTLPR	.122	1.426	.909	2.237
	AGE	.035	1.197	1.013	1.414
	*5-HTTLPR*xRLE	.726	1.085	.686	1.716

BSI: Brief Symptom Inventory; BSI-DEP: BSI depression score; BSI-ANX: BSI anxiety score; DEP: lifetime depression; RLE: recent negative life events (in the last year).

Exp(B): logistic regression coefficient (i.e. effect size of a variable in the model); *95% C.I.* : the 95% confidence interval for Exp(B); *BSI-ANX** and *BSI-DEP**: are discretized forms of the respective phenotypes. For the purposes of logistic regression only samples with 'severe' and 'low' values were used.

References

1. Friedman N (2004) Inferring cellular networks using probabilistic graphical models. Science 303: 799-805.
2. Verzilli C, Stallard N, Whittaker J (2006) Bayesian graphical models for genomewide association studies. American Journal of Human Genetics 79: 100-112.
3. Mourad R, Sinoquet C, Leray P (2012) Probabilistic graphical models for genetic association studies. Briefings in Bioinformatics 13: 20-33.
4. Ungvári I, Hullám G, Antal P, Kiszel PS, Gézsi A, et al. (2012) Evaluation of a partial genome screening of two asthma susceptibility regions using Bayesian network based Bayesian multilevel analysis of relevance. PLOS ONE.
5. Antal P, Millinghoffer A, Hullam G, Szalai C, Falus A. A bayesian view of challenges in feature selection: Feature aggregation, multiple targets, redundancy and interaction. In: Yvan Saeys HL, Iñaki Inza, Louis Wehenkel and Yves Van de Peer, editor; 2008; September 15, 2008, Antwerp, Belgium. JMLR Workshop and Conference Proceedings. pp. 74-89.
6. Hoeting JAM, D.; Raftery, A. E.; Volinsky, C. T. (1999) Bayesian Model Averaging: A Tutorial. Statistical Science 14: 382-417.
7. Madigan D, Andersson S, Perlman M, Volinsky C (1996) Bayesian model averaging and model selection for Markov equivalence classes of acyclic digraphs. Communications in Statistics-Theory and Methods 25: 2493-2519.
8. Giudici P, Castelo R (2003) Improving Markov Chain Monte Carlo model search for data mining. Machine Learning 50: 127-158.
9. Antal P, Millinghoffer A, Hullám G, Hajós G, Szalai C, et al. (2014) Bayesian, systems-based, multilevel analysis of associations for complex phenotypes: from interpretation to decisions. In: Sinoquet C, Mourad R, editors. Probabilistic Graphical Models for Genetics, Genomics and Postgenomics. USA: Oxford University Press.
10. Hullam G, Antal P. (2012) Estimation of effect size posterior using model averaging over Bayesian network structures and parameters. Proceedings of the Sixth European Workshop on Probabilistic Graphical Models. Granada, Spain. pp. 147-154
