# Supplementary material for: Variability in the Effect of 5-HTTLPR on Depression in a Large European Population: The Role of Age, Symptom Profile, Type and Intensity of Life Stressors
Source: PLoS One. 2015 Mar 6;10(3):e0116316. doi: 10.1371/journal.pone.0116316 (PMC4351953; doi:10.1371/journal.pone.0116316)
Supplement: S2 File — Data (PDF) [file pone.0116316.s002.pdf]

ID	GENDER	AGE	AGE_30	DEPR	BSI_ANX	BSI_ANX_3	BSI_DEP	BSI_DEP_3	RLE	RLE_3	CHA	CHA_3	5HTTLPR
1	2	22	1	1	0.17	1	0	1	1	0	1	1	LL
2	2	#NULL!	#NULL!	0	0	1	0	1	0	0	1	1	SL
3	2	21	1	1	1.5	2	3	3	4	2	1	1	LL
4	2	21	1	0	0.83	1	0.1	1	0	0	0	1	SL
5	1	20	1	0	0.17	1	0.6	1	1	0	1	1	SS
6	2	20	1	0	0.17	1	0	1	0	0	0	1	SL
7	2	29	1	0	0.17	1	0.5	1	1	0	1	1	SS
8	1	26	1	0	0	1	0.2	1	2	1	7	3	LL
9	2	25	1	0	0.17	1	0.2	1	0	0	6	2	SS
10	2	25	1	1	0	1	0.1	1	1	0	2	1	SL
11	2	31	2	0	0	1	0	1	1	0	3	1	SL
12	2	34	2	0	0.67	1	0.3	1	0	0	1	1	LL
13	1	22	1	0	0	1	0	1	1	0	0	1	SL
14	2	52	2	0	0	1	0.2	1	3	2	0	1	SS
15	2	22	1	0	0	1	0	1	1	0	1	1	SL
16	2	21	1	0	0	1	0.3	1	0	0	9	3	LL
17	2	23	1	0	0.67	1	0.4	1	0	0	4	2	SL
18	1	46	2	0	0	1	0.2	1	0	0	5	2	LL
19	2	39	2	0	0.5	1	0.4	1	2	1	9	3	SL
20	2	40	2	1	0.83	1	0.4	1	0	0	9	3	SL
21	2	50	2	1	0.33	1	0.4	1	0	0	11	3	SL
22	2	42	2	0	0.67	1	0.3	1	0	0	0	1	SL
23	1	25	1	0	1	2	0.2	1	3	2	15	3	SL
24	2	41	2	0	0.33	1	0.2	1	0	0	5	2	SL
25	2	26	1	0	0.67	1	0.2	1	0	0	3	1	SL
26	2	51	2	1	0	1	0	1	0	0	4	2	LL
27	2	44	2	0	0.17	1	0.1	1	1	0	4	2	LL
28	2	42	2	1	2.17	3	0.7	1	1	0	1	1	SL
29	2	46	2	0	0.67	1	0	1	0	0	2	1	LL
30	2	38	2	0	0	1	0.3	1	1	0	4	2	SL
31	2	29	1	0	1.83	2	1.4	2	1	0	4	2	LL
32	1	39	2	1	1.5	2	1.2	2	1	0	2	1	LL
33	2	26	1	0	0.5	1	0.3	1	1	0	0	1	SL
34	1	36	2	0	0.67	1	0.2	1	1	0	6	2	SL
35	2	40	2	0	0	1	0	1	1	0	4	2	LL
36	1	28	1	0	0.33	1	0	1	0	0	2	1	LL
37	2	43	2	0	0.17	1	0.2	1	0	0	0	1	SL
38	1	29	1	0	0.5	1	0.2	1	0	0	3	1	SL
39	2	29	1	0	1.17	2	2.3	3	1	0	2	1	SS
40	2	52	2	0	0.5	1	0.1	1	1	0	1	1	SS
41	2	30	1	0	0.33	1	0.4	1	0	0	9	3	SS
42	1	37	2	0	0.33	1	0.2	1	1	0	0	1	SS
43	2	25	1	0	0.17	1	0.1	1	1	0	2	1	SL
44	2	48	2	0	0	1	0	1	0	0	12	3	SL
45	2	35	2	0	0.67	1	0.5	1	2	1	1	1	SL
46	2	36	2	0	0.17	1	0	1	0	0	2	1	SS
47	2	28	1	0	0.67	1	0.3	1	0	0	1	1	LL
48	1	#NULL!	#NULL!	0	0.17	1	0	1	0	0	#NULL!	#NULL!	SL
49	2	23	1	1	1	2	1.7	2	2	1	10	3	SL
50	2	34	2	0	1.67	2	0.9	1	0	0	3	1	SL
51	2	24	1	1	3.33	3	1.7	2	2	1	7	3	SS
52	2	43	2	0	2	3	0.6	1	1	0	6	2	SL
53	2	36	2	1	0.5	1	0.1	1	4	2	8	3	LL
54	2	50	2	0	0.33	1	0.1	1	1	0	2	1	SL
55	2	27	1	0	1	2	0.3	1	1	0	1	1	SL
56	1	28	1	0	0	1	0.2	1	0	0	2	1	LL

57	2	22	1	0	1	2	1	2	2	1	1	1	SL
58	2	32	2	0	0.17	1	0	1	0	0	1	1	SS
59	1	26	1	0	2	3	1.6	2	0	0	5	2	LL
60	2	37	2	0	0.17	1	0.3	1	0	0	0	1	SS
61	2	26	1	1	0.67	1	0.4	1	0	0	0	1	SL
62	1	33	2	1	0.17	1	0	1	1	0	3	1	SS
63	1	30	1	0	0.83	1	1.2	2	2	1	0	1	SL
64	2	49	2	1	2	3	0.7	1	2	1	1	1	LL
65	2	26	1	1	1.83	2	0.7	1	4	2	6	2	LL
66	2	45	2	0	1	2	0.3	1	2	1	10	3	LL
67	2	35	2	0	0.17	1	0.2	1	1	0	5	2	SL
68	2	46	2	1	0	1	0	1	1	0	3	1	SL
69	2	54	2	0	0	1	0.3	1	1	0	2	1	LL
70	2	49	2	0	0.33	1	1.1	2	2	1	4	2	LL
71	2	48	2	0	0.67	1	0.4	1	0	0	2	1	LL
72	2	#NULL!	#NULL!	0	0.83	1	1.1	2	1	0	8	3	SS
73	2	48	2	0	0	1	0.1	1	0	0	0	1	SL
74	1	24	1	1	0.5	1	0.2	1	2	1	1	1	SL
75	1	43	2	0	0.33	1	0.3	1	0	0	3	1	LL
76	2	31	2	0	0.17	1	0.3	1	4	2	2	1	SS
77	2	54	2	0	0	1	0.3	1	2	1	5	2	SL
78	2	28	1	0	0.83	1	0.4	1	1	0	2	1	SL
79	2	33	2	0	0.83	1	0.1	1	2	1	0	1	SL
80	2	#NULL!	#NULL!	1	1.67	2	0.9	1	0	0	3	1	LL
81	2	39	2	0	0.83	1	0.2	1	0	0	1	1	SL
82	2	46	2	0	0.5	1	0.6	1	0	0	0	1	SL
83	2	25	1	1	1.17	2	0.8	1	1	0	0	1	SS
84	2	21	1	0	0	1	0.3	1	0	0	3	1	LL
85	2	36	2	0	0.33	1	0.1	1	0	0	0	1	LL
86	1	28	1	0	1.17	2	0.8	1	2	1	3	1	SL
87	2	54	2	0	0	1	0.8	1	1	0	13	3	SL
88	2	27	1	0	0	1	0.4	1	0	0	2	1	LL
89	2	34	2	1	1.17	2	1.6	2	1	0	10	3	LL
90	2	24	1	0	0.17	1	0	1	0	0	2	1	LL
91	2	28	1	0	0.33	1	0.4	1	3	2	1	1	LL
92	1	32	2	0	0	1	0	1	0	0	0	1	SL
93	1	28	1	0	0.83	1	0.9	1	0	0	6	2	SS
94	2	27	1	1	0.33	1	0.3	1	1	0	4	2	LL
95	2	43	2	1	0.17	1	0.1	1	0	0	8	3	LL
96	2	37	2	0	0.83	1	0.9	1	1	0	0	1	SL
97	2	36	2	0	0.33	1	0.2	1	1	0	4	2	LL
98	2	30	1	0	1	2	0.5	1	0	0	3	1	LL
99	2	24	1	0	1.17	2	0.2	1	0	0	2	1	SL
100	1	25	1	0	0	1	0.4	1	0	0	2	1	SL
101	2	26	1	0	0.33	1	0.2	1	3	2	0	1	SL
102	1	29	1	0	0.33	1	0.2	1	0	0	1	1	SL
103	2	25	1	0	1.17	2	0.1	1	1	0	4	2	SL
104	2	57	2	0	0	1	0	1	0	0	0	1	LL
105	1	56	2	0	0.83	1	1.1	2	2	1	2	1	SL
106	2	52	2	1	0	1	0.2	1	0	0	2	1	SL
107	2	42	2	0	0.17	1	0.5	1	3	2	1	1	LL
108	1	57	2	1	0.83	1	0.5	1	1	0	5	2	SL
109	1	26	1	0	0.17	1	0.3	1	1	0	8	3	SL
110	2	22	1	0	0	1	0.2	1	1	0	0	1	SL
111	2	36	2	0	0.5	1	0.2	1	1	0	0	1	SL
112	2	31	2	1	0.17	1	0.2	1	0	0	1	1	SS
113	2	32	2	1	0.25	1	0.1	1	2	1	2	1	SL
114	2	39	2	0	1.67	2	1.5	2	2	1	4	2	LL
115	2	30	1	1	0	1	0	1	1	0	2	1	SS

116	2	42	2	0	0	1	0	1	0	0	1	1	SL
117	2	40	2	0	0.17	1	0	1	0	0	2	1	SS
118	2	41	2	0	0	1	0	1	0	0	8	3	LL
119	2	52	2	0	0.33	1	0.1	1	0	0	3	1	SL
120	2	27	1	0	0.5	1	0.2	1	0	0	4	2	SL
121	2	42	2	0	2.17	3	0.8	1	0	0	0	1	SS
122	2	28	1	0	0.5	1	0.3	1	0	0	1	1	LL
123	1	36	2	0	0.83	1	0.6	1	2	1	2	1	LL
124	2	45	2	1	1	2	1	2	1	0	6	2	SL
125	2	27	1	0	0.67	1	0.3	1	1	0	1	1	SS
126	2	30	1	1	1	2	0.6	1	0	0	2	1	SS
127	2	31	2	0	0.5	1	0	1	1	0	0	1	SL
128	1	32	2	0	0.17	1	0.1	1	0	0	2	1	SL
129	2	48	2	0	0.17	1	0	1	1	0	7	3	SL
130	2	34	2	1	0	1	0	1	2	1	2	1	SL
131	2	#NULL!	#NULL!	0	0.67	1	0.1	1	0	0	8	3	LL
132	2	36	2	0	0	1	0.2	1	#NULL!	#NULL!	#NULL!	#NULL!	SL
133	1	26	1	0	1.33	2	2.8	3	2	1	13	3	SS
134	2	50	2	0	0.17	1	0.6	1	0	0	2	1	SL
135	2	34	2	0	0.67	1	0.1	1	1	0	13	3	SL
136	2	48	2	0	0	1	0.1	1	1	0	1	1	SL
137	2	#NULL!	#NULL!	0	1.5	2	0.3	1	0	0	2	1	SS
138	2	38	2	0	0.67	1	0.7	1	1	0	4	2	LL
139	2	45	2	0	1	2	0.5	1	1	0	2	1	LL
140	2	28	1	0	0	1	0	1	0	0	0	1	SL
141	2	33	2	1	0.83	1	0.1	1	1	0	3	1	LL
142	2	34	2	0	0	1	0	1	0	0	2	1	LL
143	2	45	2	0	0	1	0.3	1	1	0	2	1	SS
144	1	31	2	0	0.5	1	0.1	1	0	0	3	1	SL
145	2	36	2	0	0.5	1	0.2	1	1	0	2	1	LL
146	2	29	1	1	0.67	1	1.2	2	1	0	8	3	SL
147	2	47	2	0	0.5	1	0.1	1	1	0	1	1	LL
148	2	24	1	0	1.17	2	0.7	1	0	0	2	1	LL
149	2	32	2	0	0.33	1	0.2	1	#NULL!	#NULL!	#NULL!	#NULL!	LL
150	2	54	2	0	1.33	2	1.1	2	2	1	2	1	SL
151	2	22	1	0	0.17	1	0.6	1	2	1	1	1	LL
152	2	38	2	0	2.5	3	3.4	3	#NULL!	#NULL!	#NULL!	#NULL!	SL
153	1	49	2	0	0	1	0.3	1	0	0	5	2	SS
154	2	22	1	0	0.5	1	0.2	1	0	0	0	1	SL
155	2	#NULL!	#NULL!	0	1.17	2	0.8	1	4	2	1	1	LL
156	2	22	1	0	0	1	0	1	1	0	0	1	SL
157	2	21	1	1	0.17	1	0	1	1	0	1	1	SL
158	2	20	1	0	0.83	1	0.5	1	0	0	1	1	LL
159	2	21	1	0	0.33	1	0.3	1	1	0	4	2	SL
160	2	23	1	0	1.67	2	1.6	2	1	0	4	2	SL
161	1	24	1	0	0.17	1	0.2	1	0	0	0	1	SL
162	2	20	1	0	1.17	2	1	2	1	0	4	2	LL
163	1	21	1	0	0.67	1	0	1	1	0	0	1	LL
164	1	30	1	0	0.33	1	0.2	1	0	0	1	1	SS
165	2	22	1	0	0.33	1	0.3	1	0	0	0	1	SL
166	2	23	1	0	0.17	1	0.8	1	0	0	1	1	SL
167	2	20	1	1	3	3	1.4	2	2	1	1	1	SS
168	2	24	1	0	0.33	1	1	2	1	0	1	1	LL
169	2	21	1	0	0	1	0.4	1	0	0	2	1	SL
170	2	41	2	0	0.5	1	0	1	0	0	1	1	SS
171	2	29	1	1	1	2	0.4	1	1	0	8	3	SS
172	2	35	2	0	0	1	0	1	0	0	0	1	LL
173	2	34	2	1	0.33	1	0.1	1	1	0	1	1	LL
174	2	#NULL!	#NULL!	0	0.67	1	0	1	1	0	2	1	SS

175	2	50	2	0	0.83	1	0.7	1	0	0	1	1	SL
176	2	45	2	1	1.33	2	0.8	1	1	0	2	1	LL
177	2	24	1	0	0.33	1	0.1	1	2	1	7	3	LL
178	2	47	2	0	1.17	2	0.2	1	0	0	0	1	SL
179	2	40	2	0	0.83	1	0.2	1	1	0	2	1	SS
180	2	33	2	1	1.33	2	0.6	1	1	0	1	1	SL
181	2	33	2	0	0	1	0.1	1	1	0	0	1	LL
182	2	21	1	0	0.83	1	0.4	1	0	0	1	1	LL
183	1	24	1	0	0	1	0.4	1	2	1	1	1	LL
184	2	27	1	0	0.33	1	0	1	1	0	0	1	LL
185	2	33	2	0	0.17	1	0	1	2	1	0	1	SS
186	2	32	2	0	0.5	1	0	1	0	0	7	3	LL
187	2	28	1	1	0	1	0	1	0	0	1	1	SL
188	2	21	1	1	0.5	1	1.4	2	1	0	1	1	LL
189	2	19	1	1	0.67	1	2.8	3	3	2	1	1	LL
190	2	20	1	0	1.83	2	1.1	2	0	0	4	2	SL
191	1	20	1	0	0.83	1	0.6	1	1	0	2	1	SL
192	2	20	1	0	2	3	1.8	2	0	0	0	1	LL
193	2	21	1	0	0.83	1	0.5	1	0	0	0	1	SL
194	2	19	1	1	1.17	2	0.8	1	5	2	1	1	LL
195	2	20	1	1	2.17	3	2.2	3	2	1	2	1	SS
196	2	22	1	0	2	3	1.4	2	3	2	0	1	LL
197	2	20	1	0	1.33	2	1.2	2	2	1	4	2	LL
198	2	22	1	0	0.33	1	0.4	1	2	1	3	1	SL
199	2	22	1	0	0.67	1	0.3	1	2	1	0	1	LL
200	2	21	1	0	0.5	1	0.3	1	1	0	3	1	SL
201	2	22	1	0	0.67	1	0.9	1	0	0	2	1	SS
202	2	25	1	0	0	1	0	1	0	0	0	1	SS
203	2	19	1	1	2	3	1.5	2	2	1	2	1	SS
204	2	#NULL!	#NULL!	0	0.5	1	0.1	1	1	0	6	2	SL
205	2	22	1	0	0.67	1	0.6	1	0	0	2	1	SL
206	2	20	1	0	0.5	1	0.6	1	2	1	1	1	SL
207	2	20	1	0	0.33	1	0	1	0	0	2	1	LL
208	2	21	1	1	1.67	2	1.6	2	3	2	6	2	SS
209	2	20	1	0	1.17	2	0.3	1	0	0	1	1	SL
210	2	20	1	0	0.33	1	0	1	1	0	9	3	SL
211	2	22	1	1	1.5	2	1.4	2	1	0	6	2	LL
212	1	26	1	1	0.5	1	0.5	1	2	1	3	1	LL
213	2	22	1	0	0	1	0.4	1	0	0	0	1	SL
214	2	20	1	0	0.17	1	0.1	1	0	0	1	1	SS
215	2	21	1	0	0	1	0.1	1	0	0	3	1	SL
216	2	22	1	0	0	1	0	1	0	0	0	1	SL
217	2	20	1	0	1.83	2	0.2	1	1	0	7	3	SS
218	2	20	1	0	0.83	1	0	1	2	1	0	1	SL
219	2	19	1	0	0.83	1	0.7	1	0	0	1	1	SS
220	2	23	1	1	0.83	1	0.2	1	1	0	#NULL!	#NULL!	LL
221	2	25	1	0	0	1	0.3	1	1	0	0	1	LL
222	2	22	1	0	0	1	0.3	1	1	0	0	1	LL
223	2	23	1	0	0.17	1	0.1	1	2	1	0	1	SL
224	2	21	1	0	0	1	0	1	0	0	4	2	SS
225	2	22	1	0	0	1	0	1	1	0	1	1	SS
226	2	24	1	1	0.33	1	0	1	2	1	3	1	LL
227	2	25	1	0	0.17	1	0.7	1	2	1	6	2	SL
228	2	21	1	0	0.67	1	0.2	1	1	0	0	1	LL
229	2	22	1	0	0.17	1	0.1	1	0	0	7	3	SL
230	1	24	1	0	0.33	1	0.4	1	0	0	7	3	SL
231	1	27	1	1	0.17	1	0.1	1	0	0	0	1	LL
232	2	24	1	0	0.5	1	0.2	1	1	0	3	1	SS
233	1	24	1	1	0.67	1	0.9	1	1	0	3	1	SL

234	2	20	1	0	1	2	0.7	1	3	2	1	1	SL
235	2	22	1	0	0.83	1	0.4	1	2	1	2	1	SL
236	2	22	1	0	0.83	1	0	1	0	0	0	1	SL
237	2	57	2	0	0.5	1	0.2	1	1	0	0	1	SL
238	2	#NULL!	#NULL!	0	0.33	1	0.1	1	0	0	1	1	SS
239	2	51	2	1	0.33	1	2.2	3	1	0	2	1	LL
240	2	#NULL!	#NULL!	0	0.67	1	0.4	1	0	0	1	1	LL
241	2	53	2	0	0.67	1	1.1	2	3	2	3	1	SL
242	2	34	2	0	0.33	1	0.4	1	0	0	1	1	SL
243	2	29	1	0	0.5	1	0.1	1	0	0	1	1	LL
244	1	24	1	0	0.5	1	0.1	1	1	0	1	1	LL
245	2	24	1	0	0.5	1	0.1	1	4	2	5	2	SS
246	2	23	1	0	0.33	1	0.4	1	0	0	3	1	SL
247	2	26	1	0	0.67	1	0.1	1	1	0	1	1	SL
248	2	22	1	0	1.17	2	0.7	1	1	0	2	1	SL
249	2	25	1	0	0.5	1	0.2	1	1	0	1	1	SL
250	1	#NULL!	#NULL!	0	0	1	0	1	0	0	0	1	SL
251	2	26	1	0	0	1	0	1	0	0	0	1	LL
252	2	#NULL!	#NULL!	0	0	1	0	1	0	0	1	1	LL
253	2	21	1	0	0	1	0.1	1	0	0	1	1	LL
254	2	28	1	1	0.83	1	1.7	2	2	1	4	2	LL
255	2	38	2	1	0.17	1	0.4	1	3	2	2	1	SL
256	2	33	2	0	0	1	0	1	2	1	0	1	SL
257	2	42	2	1	0.5	1	0.3	1	0	0	12	3	SL
258	2	24	1	1	1	2	0.8	1	1	0	7	3	LL
259	2	22	1	1	0.17	1	0	1	2	1	2	1	SS
260	2	24	1	1	1	2	0.9	1	0	0	2	1	LL
261	2	22	1	0	0.5	1	0.1	1	1	0	6	2	LL
262	1	23	1	0	0	1	0.3	1	1	0	2	1	SS
263	2	22	1	1	1.33	2	1.1	2	1	0	3	1	SL
264	2	21	1	0	0.83	1	0.3	1	0	0	0	1	LL
265	2	21	1	1	0.33	1	0.2	1	2	1	3	1	LL
266	2	21	1	0	0.33	1	0	1	1	0	0	1	SL
267	2	22	1	0	0.67	1	0.3	1	0	0	0	1	SS
268	2	22	1	1	0.83	1	0.5	1	0	0	2	1	LL
269	2	32	2	0	0.17	1	0.1	1	0	0	0	1	LL
270	1	24	1	0	0.17	1	0.3	1	2	1	1	1	SS
271	2	21	1	0	0.83	1	0.8	1	0	0	#NULL!	#NULL!	SL
272	2	22	1	0	0.33	1	0.1	1	0	0	0	1	SL
273	2	18	1	0	0.33	1	0	1	1	0	0	1	SL
274	2	21	1	0	0.83	1	0.6	1	5	2	2	1	LL
275	2	32	2	1	0.5	1	0.2	1	0	0	3	1	LL
276	2	27	1	0	1.17	2	0.3	1	2	1	1	1	LL
277	2	#NULL!	#NULL!	0	0.67	1	1.7	2	1	0	0	1	LL
278	2	#NULL!	#NULL!	0	0.5	1	0.3	1	0	0	0	1	LL
279	2	31	2	0	0	1	0	1	1	0	0	1	LL
280	2	30	1	0	0	1	0	1	1	0	2	1	SL
281	2	25	1	0	0.83	1	0.3	1	2	1	9	3	SL
282	2	#NULL!	#NULL!	0	0.5	1	0.1	1	1	0	2	1	SL
283	1	40	2	0	1.83	2	1.1	2	0	0	2	1	SL
284	2	49	2	0	0.33	1	0.1	1	0	0	3	1	SL
285	2	22	1	0	0.67	1	0.1	1	3	2	2	1	SL
286	2	30	1	1	0.5	1	0.3	1	4	2	4	2	LL
287	2	26	1	1	1	2	0.2	1	1	0	4	2	SL
288	2	37	2	0	0.83	1	0.2	1	0	0	2	1	LL
289	2	#NULL!	#NULL!	0	0.17	1	0.1	1	0	0	1	1	SL
290	1	35	2	0	0	1	0	1	0	0	0	1	SL
291	2	47	2	1	0.33	1	0.1	1	0	0	12	3	LL
292	2	48	2	0	0	1	0	1	0	0	3	1	LL

293	2	41	2	0	0.17	1	0.2	1	1	0	0	1	LL
294	2	25	1	0	1.33	2	1.1	2	4	2	11	3	SL
295	2	33	2	0	1.33	2	0	1	2	1	2	1	SL
296	2	19	1	0	1	2	1	2	2	1	0	1	SL
297	1	28	1	0	0.83	1	1	2	1	0	0	1	LL
298	2	43	2	0	0.17	1	0.1	1	0	0	6	2	LL
299	2	31	2	0	0	1	0	1	0	0	3	1	SL
300	2	43	2	0	0.67	1	0.2	1	0	0	4	2	SL
301	2	48	2	1	1.17	2	0.8	1	0	0	1	1	LL
302	2	29	1	1	1.5	2	2.4	3	1	0	1	1	LL
303	2	29	1	0	1.17	2	0.7	1	0	0	2	1	SS
304	2	34	2	0	0.4	1	0.5	1	0	0	2	1	SS
305	2	#NULL!	#NULL!	1	1.17	2	1.4	2	1	0	13	3	LL
306	2	31	2	0	0	1	0	1	0	0	2	1	SS
307	2	47	2	0	0.33	1	0.1	1	1	0	6	2	SS
308	2	30	1	0	0	1	0	1	1	0	2	1	LL
309	2	40	2	0	1.17	2	0.7	1	0	0	5	2	LL
310	2	51	2	0	0	1	0	1	1	0	4	2	LL
311	2	42	2	0	1	2	1.2	2	1	0	2	1	SL
312	2	28	1	0	0	1	0.1	1	1	0	0	1	LL
313	2	29	1	1	2	3	0.9	1	1	0	2	1	LL
314	2	54	2	0	1.17	2	1.1	2	0	0	13	3	SL
315	1	50	2	0	0.17	1	0	1	1	0	2	1	SL
316	2	51	2	0	0.17	1	0.1	1	0	0	0	1	SL
317	2	49	2	0	1.17	2	0.3	1	1	0	0	1	SL
318	1	55	2	0	0.17	1	0.1	1	0	0	0	1	SL
319	1	42	2	0	0.5	1	0.3	1	1	0	0	1	SL
320	1	#NULL!	#NULL!	0	0	1	0.2	1	0	0	1	1	SL
321	2	42	2	0	0	1	0.2	1	2	1	0	1	LL
322	2	30	1	0	0.33	1	0.2	1	1	0	1	1	SL
323	2	41	2	0	0.5	1	0.3	1	2	1	0	1	LL
324	2	40	2	0	1.33	2	0.7	1	0	0	2	1	SL
325	1	36	2	1	#NULL!	#NULL!	#NULL!	#NULL!	2	1	2	1	LL
326	1	#NULL!	#NULL!	0	0	1	0	1	0	0	8	3	LL
327	1	41	2	0	0.33	1	0.4	1	2	1	2	1	LL
328	1	#NULL!	#NULL!	0	0.17	1	0	1	0	0	1	1	SL
329	1	40	2	0	1	2	0.3	1	2	1	2	1	LL
330	1	52	2	0	0.5	1	0.1	1	1	0	1	1	SL
331	1	52	2	0	0.33	1	0	1	0	0	2	1	SL
332	2	32	2	0	0.5	1	0.4	1	3	2	4	2	SL
333	1	28	1	0	0.67	1	0	1	0	0	1	1	SL
334	2	23	1	0	0.83	1	1	2	1	0	2	1	SL
335	2	50	2	0	0.5	1	0	1	0	0	0	1	LL
336	2	38	2	0	0	1	0	1	3	2	0	1	LL
337	2	33	2	0	0.67	1	1.1	2	0	0	2	1	SL
338	2	48	2	0	0	1	0	1	0	0	0	1	LL
339	2	44	2	1	0.33	1	0.2	1	0	0	0	1	LL
340	2	47	2	0	0.33	1	0	1	0	0	0	1	SL
341	2	37	2	0	1.67	2	0.9	1	3	2	2	1	SL
342	2	51	2	0	0	1	0.3	1	1	0	2	1	LL
343	2	45	2	0	0	1	1.8	2	1	0	4	2	SL
344	1	28	1	0	0	1	0.2	1	1	0	2	1	SL
345	2	54	2	1	3.17	3	2.9	3	0	0	15	3	SS
346	1	#NULL!	#NULL!	0	0.33	1	0.1	1	0	0	0	1	SL
347	1	37	2	0	0.17	1	0.3	1	1	0	9	3	LL
348	1	31	2	0	0	1	0	1	3	2	6	2	SS
349	1	#NULL!	#NULL!	1	3.33	3	3.1	3	4	2	1	1	SL
350	2	23	1	0	0.67	1	1.1	2	0	0	5	2	SL
351	2	22	1	0	0.67	1	0.2	1	2	1	0	1	SL

352	2	46	2	0	0.17	1	0.1	1	0	0	1	1	LL
353	2	35	2	0	0	1	0.3	1	0	0	0	1	SL
354	1	44	2	0	0.33	1	0.4	1	1	0	1	1	SL
355	2	40	2	0	0.33	1	0.2	1	2	1	2	1	LL
356	2	52	2	0	0.67	1	0.3	1	4	2	0	1	LL
357	2	36	2	1	1.17	2	0.7	1	3	2	0	1	LL
358	2	19	1	1	0	1	0.3	1	1	0	0	1	LL
359	2	19	1	0	1.5	2	0.8	1	3	2	4	2	SL
360	2	19	1	0	0.83	1	0.1	1	2	1	1	1	SL
361	2	19	1	0	0.17	1	0	1	2	1	2	1	SS
362	2	18	1	0	0.33	1	0.2	1	1	0	0	1	SL
363	2	20	1	1	0.5	1	0.4	1	1	0	0	1	SL
364	2	20	1	0	1.83	2	0.1	1	0	0	1	1	LL
365	2	23	1	0	0.67	1	0.7	1	0	0	1	1	SL
366	2	20	1	1	0.5	1	0.3	1	1	0	2	1	SL
367	2	19	1	0	1	2	0.2	1	1	0	0	1	SL
368	2	21	1	0	0.33	1	0	1	0	0	4	2	SL
369	2	18	1	0	#NULL!	#NULL!	#NULL!	#NULL!	1	0	2	1	SL
370	2	19	1	0	0.33	1	0.2	1	0	0	0	1	LL
371	2	19	1	0	0	1	0	1	0	0	0	1	LL
372	2	19	1	0	0.5	1	0.5	1	0	0	2	1	LL
373	2	19	1	0	0.67	1	0.2	1	0	0	2	1	SL
374	2	18	1	0	1	2	0.7	1	0	0	1	1	LL
375	2	19	1	1	0.33	1	0.6	1	0	0	2	1	SL
376	1	18	1	1	1.33	2	2.7	3	2	1	6	2	LL
377	1	20	1	0	0.17	1	0.1	1	0	0	2	1	SS
378	2	20	1	0	1	2	0	1	1	0	4	2	SL
379	2	19	1	0	3.33	3	2.1	3	2	1	1	1	SL
380	2	29	1	1	0.5	1	0.5	1	0	0	2	1	SL
381	2	20	1	0	1	2	0.7	1	2	1	7	3	SL
382	2	19	1	0	0.5	1	0	1	0	0	5	2	LL
383	2	39	2	0	0.33	1	0	1	0	0	1	1	SS
384	2	20	1	1	1.17	2	1.1	2	4	2	0	1	SL
385	2	21	1	0	0.5	1	0.4	1	0	0	2	1	LL
386	2	23	1	1	2.17	3	2.1	3	1	0	1	1	SS
387	2	29	1	0	1.33	2	1.5	2	0	0	2	1	SS
388	1	26	1	0	0	1	0.1	1	1	0	2	1	LL
389	2	18	1	0	0.17	1	0.3	1	0	0	1	1	SL
390	2	20	1	1	1.5	2	0.7	1	0	0	2	1	LL
391	2	19	1	0	0	1	0	1	2	1	0	1	LL
392	1	30	1	0	0.17	1	0	1	1	0	1	1	SL
393	2	28	1	0	0.17	1	0	1	0	0	6	2	SL
394	2	41	2	0	0.5	1	1.4	2	1	0	9	3	SL
395	2	29	1	0	1.17	2	0.5	1	1	0	0	1	LL
396	2	40	2	0	0.33	1	0	1	1	0	2	1	LL
397	2	34	2	0	0.5	1	0.5	1	1	0	5	2	SL
398	2	40	2	0	0.33	1	0.3	1	2	1	0	1	LL
399	2	39	2	0	0.67	1	0.3	1	2	1	5	2	SL
400	2	43	2	0	0.67	1	0.2	1	1	0	1	1	SL
401	2	#NULL!	#NULL!	0	0	1	0	1	0	0	2	1	LL
402	1	40	2	0	0.33	1	0.2	1	1	0	7	3	LL
403	2	41	2	0	0.5	1	0.1	1	0	0	0	1	SS
404	2	37	2	0	0.83	1	0.3	1	1	0	3	1	SL
405	2	40	2	0	0.33	1	0.1	1	3	2	4	2	SL
406	2	44	2	0	1.17	2	0.4	1	2	1	0	1	LL
407	2	38	2	0	0.5	1	0.4	1	2	1	7	3	SL
408	2	39	2	1	1	2	0.5	1	0	0	2	1	SS
409	2	#NULL!	#NULL!	0	0	1	0	1	0	0	4	2	SL
410	2	30	1	1	0.33	1	1.3	2	0	0	2	1	LL

411	2	33	2	0	0.17	1	0	1	0	0	0	1	SL
412	2	36	2	0	0.33	1	0.1	1	2	1	4	2	LL
413	2	27	1	0	1.5	2	0.5	1	1	0	5	2	SL
414	2	40	2	0	0.17	1	0.1	1	4	2	0	1	LL
415	2	41	2	0	0.33	1	0.1	1	1	0	0	1	SL
416	2	28	1	1	2.33	3	2.9	3	0	0	2	1	SS
417	2	39	2	0	0.5	1	0.3	1	0	0	4	2	SL
418	2	54	2	0	0.33	1	0.2	1	0	0	4	2	SL
419	2	50	2	0	0.67	1	0.5	1	5	2	4	2	SL
420	2	33	2	0	1.33	2	0.1	1	0	0	7	3	SL
421	2	34	2	0	0.5	1	0.3	1	0	0	1	1	SL
422	2	44	2	0	0.5	1	0.7	1	1	0	9	3	SL
423	2	31	2	1	0.17	1	0.1	1	2	1	6	2	LL
424	2	35	2	0	0	1	0	1	1	0	2	1	SS
425	2	44	2	1	0.83	1	1.3	2	0	0	2	1	SL
426	2	40	2	0	0.5	1	0	1	0	0	2	1	SS
427	2	32	2	1	0.5	1	0.4	1	0	0	6	2	SS
428	2	30	1	0	1	2	1.1	2	4	2	1	1	LL
429	2	48	2	0	0	1	0.2	1	1	0	6	2	SS
430	2	41	2	0	0.33	1	0.1	1	1	0	3	1	SL
431	2	43	2	0	1	2	0.1	1	2	1	13	3	SL
432	2	42	2	0	0.5	1	0.1	1	1	0	1	1	SS
433	2	38	2	1	0.67	1	0.6	1	0	0	0	1	SL
434	2	28	1	0	0.33	1	0.1	1	1	0	6	2	SL
435	2	32	2	1	0.83	1	0.3	1	1	0	4	2	SS
436	1	31	2	0	0.5	1	0.2	1	0	0	7	3	SL
437	2	38	2	0	0.17	1	0.3	1	0	0	0	1	SL
438	2	37	2	0	0.67	1	0	1	0	0	3	1	SL
439	2	#NULL!	#NULL!	1	0.5	1	0.1	1	0	0	6	2	SL
440	2	37	2	1	0	1	0.2	1	2	1	2	1	SL
441	2	19	1	0	2.33	3	1.4	2	0	0	4	2	SL
442	2	19	1	0	0.5	1	0	1	1	0	1	1	LL
443	2	45	2	0	0.33	1	0	1	1	0	1	1	LL
444	2	34	2	0	1.17	2	1.6	2	0	0	9	3	LL
445	2	31	2	0	0.5	1	0.6	1	3	2	1	1	LL
446	2	28	1	0	0	1	0	1	0	0	9	3	SS
447	2	41	2	0	1	2	0.1	1	1	0	1	1	SL
448	2	20	1	1	0	1	1.2	2	2	1	0	1	LL
449	2	27	1	0	0.33	1	0.7	1	0	0	2	1	SL
450	2	34	2	0	0.17	1	0.3	1	2	1	1	1	LL
451	2	48	2	0	0	1	0.2	1	0	0	4	2	LL
452	2	32	2	0	1.83	2	1.2	2	0	0	5	2	SS
453	1	#NULL!	#NULL!	0	0.17	1	0	1	1	0	2	1	SL
454	2	28	1	0	0.33	1	0	1	2	1	3	1	SL
455	2	57	2	0	0.67	1	0.9	1	2	1	5	2	SL
456	2	41	2	0	0	1	0	1	0	0	3	1	SL
457	1	33	2	1	2.17	3	2.5	3	3	2	13	3	LL
458	2	32	2	1	0.67	1	0.3	1	1	0	3	1	LL
459	2	41	2	0	1.33	2	1.6	2	1	0	1	1	SS
460	2	55	2	0	0.33	1	1.2	2	1	0	3	1	SL
461	1	27	1	0	2.17	3	2.4	3	2	1	1	1	LL
462	1	48	2	1	2.83	3	1.6	2	1	0	2	1	SS
463	1	33	2	0	0.67	1	0.2	1	0	0	5	2	SL
464	2	34	2	0	1.17	2	0.5	1	0	0	2	1	SS
465	2	33	2	0	0	1	0.3	1	1	0	1	1	SS
466	1	53	2	0	0.5	1	0.7	1	1	0	1	1	LL
467	2	53	2	0	0.67	1	1.7	2	1	0	2	1	SL
468	2	22	1	1	1	2	0.4	1	2	1	10	3	SL
469	1	24	1	0	0.67	1	1.2	2	1	0	1	1	LL

470	1	28	1	0	0	1	0.1	1	0	0	2	1	SL
471	1	57	2	0	0.17	1	0	1	1	0	5	2	SL
472	1	52	2	0	0	1	0	1	0	0	0	1	SL
473	1	57	2	0	0.5	1	0.3	1	0	0	1	1	SL
474	1	32	2	1	0.67	1	1.1	2	4	2	9	3	SS
475	1	49	2	0	2.67	3	1.5	2	1	0	9	3	LL
476	2	40	2	1	0.17	1	0	1	2	1	4	2	LL
477	2	31	2	0	0.83	1	0.6	1	1	0	0	1	SL
478	2	35	2	0	0.33	1	0.3	1	0	0	3	1	SL
479	2	52	2	0	0.83	1	0.1	1	0	0	2	1	SL
480	2	48	2	1	0.83	1	0.2	1	2	1	2	1	SL
481	1	53	2	0	0.33	1	0.1	1	1	0	7	3	SL
482	1	57	2	0	0.83	1	0.44	1	0	0	5	2	LL
483	1	54	2	0	0.5	1	0.4	1	1	0	4	2	LL
484	1	#NULL!	#NULL!	0	1.83	2	1.8	2	0	0	#NULL!	#NULL!	SL
485	1	39	2	0	0.33	1	0.8	1	0	0	1	1	LL
486	1	41	2	0	0.17	1	0.1	1	0	0	4	2	LL
487	1	48	2	1	1.5	2	2.5	3	0	0	6	2	SL
488	1	53	2	0	0	1	0	1	1	0	11	3	LL
489	1	#NULL!	#NULL!	0	2.17	3	0.9	1	1	0	3	1	LL
490	2	48	2	1	2.33	3	3.8	3	5	2	10	3	SL
491	2	32	2	0	0.67	1	0.7	1	3	2	6	2	LL
492	2	53	2	1	2.33	3	3	3	1	0	7	3	SL
493	2	25	1	0	0.83	1	0.2	1	1	0	1	1	LL
494	2	#NULL!	#NULL!	0	0.67	1	0.1	1	1	0	1	1	LL
495	1	38	2	0	0.17	1	0	1	0	0	4	2	LL
496	1	24	1	1	2.5	3	2.7	3	1	0	11	3	SL
497	2	28	1	0	0.67	1	0.5	1	0	0	1	1	SL
498	1	38	2	0	0.5	1	0.3	1	2	1	3	1	LL
499	1	#NULL!	#NULL!	1	3.33	3	1.9	2	1	0	0	1	SL
500	2	46	2	0	2.17	3	1.5	2	3	2	2	1	LL
501	1	30	1	0	0	1	0	1	0	0	0	1	SS
502	2	24	1	1	1.67	2	1.1	2	3	2	4	2	LL
503	1	28	1	0	0.33	1	0.2	1	0	0	1	1	SL
504	1	60	2	1	0.67	1	1.8	2	0	0	2	1	SL
505	1	31	2	1	0.6	1	0	1	2	1	2	1	SL
506	2	21	1	0	0	1	0	1	0	0	0	1	LL
507	2	36	2	0	0.5	1	0	1	0	0	1	1	SL
508	2	20	1	0	0.17	1	0.5	1	0	0	0	1	SL
509	1	28	1	1	2.5	3	2.6	3	3	2	9	3	LL
510	1	26	1	0	0.67	1	0.7	1	1	0	0	1	SL
511	2	34	2	1	0.83	1	1.1	2	2	1	12	3	SS
512	2	59	2	1	2.17	3	0.6	1	1	0	1	1	SL
513	2	25	1	0	0	1	0	1	3	2	5	2	LL
514	2	51	2	1	3.67	3	3.1	3	1	0	13	3	SS
515	2	49	2	1	2.5	3	2.1	3	0	0	9	3	SL
516	2	31	2	1	1.83	2	1.1	2	1	0	1	1	SL
517	2	26	1	0	1.67	2	2.2	3	0	0	13	3	SS
518	2	#NULL!	#NULL!	0	0.17	1	0.6	1	3	2	0	1	LL
519	2	32	2	0	0.17	1	0.7	1	2	1	5	2	LL
520	2	24	1	0	1	2	0.5	1	1	0	4	2	LL
521	2	43	2	0	2	3	1.7	2	2	1	1	1	LL
522	2	33	2	0	0.33	1	0.2	1	1	0	3	1	SL
523	2	#NULL!	#NULL!	0	0.67	1	0.6	1	1	0	0	1	SL
524	2	31	2	0	0.5	1	0.1	1	2	1	1	1	SL
525	2	30	1	0	0.33	1	0.4	1	3	2	1	1	LL
526	2	26	1	0	1	2	0.9	1	4	2	1	1	LL
527	2	34	2	0	0.83	1	1.3	2	2	1	0	1	SS
528	2	33	2	0	1.33	2	0.2	1	3	2	2	1	LL

529	2	#NULL!	#NULL!	1	1.5	2	1	2	3	2	4	2	SL
530	2	38	2	1	0.83	1	0.5	1	1	0	1	1	SL
531	2	40	2	0	0.5	1	0.3	1	1	0	2	1	LL
532	2	37	2	1	2.17	3	0.9	1	0	0	1	1	SL
533	2	38	2	0	0.5	1	0.9	1	1	0	4	2	SS
534	2	40	2	0	0.33	1	0.2	1	0	0	3	1	SS
535	2	#NULL!	#NULL!	0	1.17	2	0.1	1	1	0	0	1	LL
536	1	26	1	0	0.33	1	1.1	2	2	1	4	2	LL
537	2	23	1	0	0.67	1	0.2	1	2	1	4	2	SS
538	1	40	2	0	0.67	1	0.4	1	0	0	3	1	SL
539	1	42	2	0	1.33	2	2.4	3	3	2	6	2	SL
540	2	35	2	0	1.83	2	0.4	1	2	1	5	2	SL
541	1	#NULL!	#NULL!	0	0	1	0	1	0	0	0	1	SL
542	2	53	2	0	0.17	1	0.2	1	0	0	0	1	SL
543	1	38	2	0	0.5	1	0.3	1	0	0	5	2	LL
544	2	52	2	0	1.17	2	1.9	2	1	0	0	1	LL
545	2	51	2	0	0.67	1	1.3	2	1	0	0	1	SS
546	2	43	2	0	0	1	0	1	0	0	2	1	LL
547	1	43	2	0	#NULL!	#NULL!	#NULL!	#NULL!	1	0	0	1	LL
548	1	29	1	0	2	3	2.7	3	2	1	3	1	SS
549	2	#NULL!	#NULL!	0	0.33	1	0.2	1	0	0	7	3	SL
550	2	23	1	0	0.17	1	0.3	1	1	0	2	1	SL
551	2	50	2	0	0.67	1	1.3	2	1	0	12	3	SL
552	2	50	2	1	1.67	2	0.9	1	5	2	10	3	SL
553	2	54	2	0	0.83	1	0.1	1	0	0	0	1	SL
554	2	19	1	1	2.33	3	1	2	0	0	7	3	SL
555	2	20	1	0	0.83	1	2.2	3	1	0	2	1	SL
556	2	42	2	1	0.5	1	0.7	1	2	1	8	3	SL
557	2	20	1	1	0.83	1	0.1	1	2	1	0	1	SL
558	2	19	1	0	0.33	1	0.1	1	0	0	2	1	SL
559	2	21	1	0	0.5	1	0.1	1	2	1	0	1	SL
560	2	22	1	0	0	1	0.1	1	0	0	0	1	LL
561	2	21	1	1	0.67	1	0.8	1	2	1	9	3	SL
562	2	#NULL!	#NULL!	0	0.67	1	0.1	1	0	0	1	1	SL
563	2	29	1	1	1.33	2	2.2	3	0	0	6	2	LL
564	1	49	2	1	1.5	2	1.4	2	6	2	8	3	SL
565	2	47	2	1	3	3	3.2	3	2	1	10	3	SL
566	2	#NULL!	#NULL!	0	0	1	0.1	1	0	0	0	1	SS
567	2	19	1	0	1.33	2	1.5	2	1	0	7	3	SL
568	2	23	1	1	0.33	1	0.3	1	0	0	3	1	LL
569	2	19	1	0	0.33	1	0.9	1	2	1	2	1	SL
570	2	20	1	1	3.5	3	3.1	3	1	0	6	2	SL
571	2	23	1	0	0.83	1	0.3	1	0	0	1	1	SL
572	1	20	1	0	0	1	0.2	1	0	0	0	1	SL
573	2	20	1	0	1.5	2	0.8	1	1	0	3	1	LL
574	2	#NULL!	#NULL!	0	0.5	1	0.3	1	1	0	2	1	LL
575	2	#NULL!	#NULL!	0	2	3	2.5	3	2	1	13	3	LL
576	2	20	1	0	2.17	3	2.4	3	2	1	3	1	SL
577	2	#NULL!	#NULL!	0	0.5	1	1	2	0	0	0	1	SS
578	2	21	1	0	1.67	2	0.1	1	1	0	0	1	SL
579	2	21	1	0	0	1	0	1	1	0	1	1	SL
580	2	23	1	1	0.5	1	0.6	1	0	0	1	1	LL
581	2	21	1	0	0.5	1	0.2	1	0	0	1	1	SL
582	1	21	1	0	2.17	3	2.2	3	6	2	3	1	SL
583	2	20	1	0	1.17	2	0.6	1	1	0	9	3	LL
584	2	22	1	0	1	2	0.3	1	0	0	5	2	LL
585	2	21	1	0	0.5	1	1.4	2	4	2	5	2	SL
586	2	21	1	0	2	3	0.7	1	2	1	1	1	SL
587	2	23	1	0	0.17	1	0.3	1	1	0	2	1	SL

588	2	21	1	0	0.17	1	0	1	3	2	2	1	LL
589	2	20	1	1	1.17	2	1.6	2	2	1	7	3	SL
590	2	22	1	0	0.17	1	0.1	1	1	0	0	1	LL
591	1	21	1	0	0	1	0.4	1	0	0	1	1	LL
592	2	21	1	0	0.33	1	0.7	1	1	0	1	1	SS
593	2	19	1	0	0.83	1	0.8	1	2	1	0	1	SL
594	2	21	1	0	0	1	0	1	0	0	2	1	LL
595	2	26	1	0	0.5	1	0.2	1	1	0	0	1	SL
596	2	20	1	0	0.33	1	0.2	1	3	2	3	1	LL
597	1	24	1	0	1.67	2	1	2	1	0	3	1	SL
598	2	20	1	1	2.17	3	1.6	2	3	2	4	2	SL
599	2	20	1	0	0.5	1	0.5	1	1	0	1	1	LL
600	2	21	1	0	0.83	1	0.8	1	3	2	0	1	SS
601	2	22	1	1	0.17	1	0	1	2	1	8	3	SL
602	2	21	1	0	0.5	1	0.4	1	1	0	2	1	SL
603	2	20	1	0	0.17	1	0.1	1	1	0	0	1	SL
604	2	21	1	0	1.33	2	0.5	1	3	2	2	1	LL
605	2	21	1	1	2.33	3	1.8	2	1	0	6	2	LL
606	2	36	2	0	0.67	1	0.2	1	0	0	2	1	LL
607	2	20	1	0	0.33	1	0.2	1	0	0	2	1	SL
608	2	21	1	0	0	1	0.3	1	3	2	0	1	SL
609	2	20	1	0	0	1	0	1	2	1	0	1	SL
610	2	20	1	0	0.5	1	0	1	1	0	0	1	LL
611	2	21	1	0	1.33	2	0.8	1	0	0	0	1	SS
612	2	21	1	0	0	1	0.2	1	0	0	0	1	SL
613	2	21	1	0	0.33	1	0.1	1	1	0	0	1	SL
614	2	20	1	1	2.33	3	1.5	2	2	1	6	2	SL
615	2	20	1	0	0.83	1	0.8	1	3	2	1	1	LL
616	2	22	1	0	0.17	1	0	1	3	2	1	1	LL
617	2	21	1	0	0.5	1	0.3	1	0	0	0	1	SL
618	2	22	1	1	2.33	3	1.9	2	1	0	1	1	SL
619	1	22	1	0	0	1	0	1	0	0	0	1	LL
620	1	21	1	0	0	1	0.1	1	0	0	0	1	LL
621	1	23	1	1	0.67	1	1.2	2	1	0	1	1	SL
622	2	21	1	0	0.67	1	0.3	1	1	0	5	2	SL
623	2	20	1	1	1.67	2	1.3	2	2	1	4	2	SS
624	2	28	1	0	0.33	1	0.2	1	0	0	0	1	SL
625	2	21	1	0	3	3	1.6	2	0	0	4	2	LL
626	2	22	1	0	0.5	1	0	1	0	0	2	1	LL
627	2	21	1	0	0	1	0.1	1	1	0	1	1	LL
628	2	22	1	0	1	2	0.5	1	3	2	2	1	LL
629	2	21	1	0	0.67	1	0.5	1	1	0	1	1	LL
630	2	22	1	0	0.67	1	0.4	1	2	1	5	2	SL
631	2	22	1	0	0.17	1	0.6	1	3	2	7	3	LL
632	2	24	1	0	1.67	2	0.4	1	2	1	7	3	LL
633	2	21	1	0	0	1	0.5	1	1	0	1	1	SS
634	2	20	1	1	0.67	1	0.7	1	3	2	0	1	LL
635	2	20	1	0	2.33	3	0.5	1	2	1	1	1	SS
636	2	20	1	0	0.33	1	0.2	1	2	1	4	2	LL
637	2	22	1	0	1.17	2	0.7	1	1	0	2	1	SS
638	2	22	1	0	0	1	0.3	1	0	0	0	1	LL
639	2	21	1	0	0.5	1	0.1	1	1	0	5	2	LL
640	2	22	1	0	0.67	1	0.1	1	2	1	0	1	SL
641	2	22	1	0	0.33	1	0.2	1	2	1	4	2	LL
642	2	21	1	0	0.17	1	0.4	1	0	0	0	1	LL
643	2	21	1	0	0	1	0.2	1	0	0	1	1	LL
644	2	21	1	1	1.17	2	2.6	3	2	1	6	2	SL
645	2	20	1	0	0.33	1	0.1	1	0	0	11	3	SS
646	1	21	1	0	1	2	0.6	1	0	0	1	1	SL

647	2	22	1	1	0.67	1	0.4	1	0	0	3	1	SL
648	2	20	1	1	1.17	2	0.5	1	2	1	2	1	SL
649	2	20	1	0	0.67	1	0.9	1	0	0	2	1	LL
650	2	#NULL!	#NULL!	0	0.83	1	0.1	1	1	0	3	1	SS
651	2	21	1	0	0	1	0	1	1	0	0	1	LL
652	2	21	1	1	1.5	2	2.3	3	1	0	1	1	LL
653	2	20	1	0	0.83	1	0.6	1	0	0	6	2	SS
654	2	25	1	0	0.17	1	0.2	1	3	2	4	2	SS
655	2	21	1	0	0.67	1	1.4	2	1	0	3	1	SS
656	2	21	1	1	0.5	1	0.4	1	3	2	3	1	LL
657	2	23	1	1	0.83	1	0.2	1	1	0	0	1	SS
658	2	23	1	0	0.33	1	0.3	1	1	0	0	1	SL
659	2	22	1	0	0.33	1	0	1	2	1	0	1	SL
660	2	23	1	0	0.33	1	0.3	1	3	2	3	1	SL
661	2	25	1	1	0	1	0.9	1	1	0	0	1	LL
662	2	30	1	0	0.33	1	0.2	1	1	0	1	1	SL
663	2	25	1	1	0.67	1	0.6	1	2	1	2	1	LL
664	2	22	1	0	1.33	2	0.8	1	1	0	3	1	LL
665	2	21	1	0	0.17	1	0	1	2	1	6	2	LL
666	2	22	1	0	0.67	1	0.5	1	0	0	1	1	SL
667	2	21	1	0	0.5	1	0.3	1	2	1	1	1	SL
668	1	22	1	0	0.33	1	0	1	1	0	2	1	LL
669	1	20	1	0	0.17	1	1.3	2	0	0	0	1	LL
670	2	19	1	0	1.67	2	1	2	2	1	2	1	SL
671	2	20	1	0	0.17	1	0	1	0	0	0	1	SL
672	2	33	2	0	0.83	1	0.4	1	0	0	2	1	SS
673	2	25	1	0	0.33	1	0.6	1	1	0	1	1	SL
674	2	#NULL!	#NULL!	0	0.33	1	0.9	1	0	0	0	1	SS
675	1	20	1	1	0.17	1	0.1	1	1	0	3	1	SL
676	2	21	1	0	2.33	3	2.1	3	1	0	1	1	SL
677	2	30	1	1	1.67	2	1.6	2	3	2	6	2	LL
678	1	25	1	1	0.17	1	0.6	1	8	2	0	1	SL
679	2	23	1	0	0.83	1	1.7	2	0	0	9	3	SL
680	2	21	1	0	1.33	2	0.6	1	2	1	6	2	SS
681	1	21	1	1	1.83	2	1.5	2	3	2	1	1	SS
682	2	21	1	0	0.17	1	0.2	1	1	0	0	1	SL
683	1	22	1	0	0.17	1	0	1	1	0	1	1	SL
684	2	21	1	0	0.83	1	1	2	4	2	0	1	LL
685	2	20	1	0	1	2	0.3	1	0	0	2	1	SL
686	2	20	1	1	3	3	1.4	2	4	2	8	3	LL
687	2	24	1	0	1.17	2	0.9	1	1	0	10	3	SS
688	2	21	1	0	1	2	0.1	1	1	0	3	1	SL
689	1	#NULL!	#NULL!	0	0.33	1	0.7	1	0	0	2	1	SL
690	1	20	1	0	0.67	1	0.7	1	1	0	2	1	LL
691	2	20	1	0	1.67	2	0.9	1	3	2	1	1	LL
692	2	19	1	1	2.33	3	1.5	2	0	0	0	1	SL
693	2	34	2	0	0.83	1	0.1	1	1	0	0	1	LL
694	2	21	1	0	2.67	3	1.6	2	2	1	1	1	SL
695	2	22	1	0	0	1	0.1	1	#NULL!	#NULL!	#NULL!	#NULL!	LL
696	2	22	1	1	1.67	2	1.4	2	1	0	6	2	SL
697	1	38	2	1	0.33	1	0.6	1	1	0	3	1	SL
698	2	20	1	0	1.33	2	1.6	2	3	2	6	2	LL
699	2	21	1	0	0.17	1	0.5	1	0	0	3	1	LL
700	2	20	1	0	0.17	1	0.1	1	0	0	0	1	SL
701	2	22	1	0	0.67	1	0	1	2	1	0	1	SS
702	2	22	1	0	0.33	1	0.1	1	1	0	1	1	SL
703	2	32	2	0	0	1	0.3	1	2	1	2	1	SS
704	2	20	1	1	0.17	1	0	1	1	0	2	1	SL
705	2	21	1	0	2	3	1.3	2	3	2	0	1	SL

706	2	21	1	0	1	2	0.7	1	1	0	3	1	LL
707	2	21	1	0	0.33	1	0.9	1	0	0	1	1	SL
708	1	21	1	0	0.33	1	0.2	1	1	0	1	1	SS
709	2	20	1	0	0.33	1	0.5	1	1	0	2	1	LL
710	1	#NULL!	#NULL!	0	0.83	1	0.8	1	0	0	6	2	LL
711	2	23	1	0	0.33	1	0.4	1	0	0	1	1	LL
712	2	39	2	0	0.17	1	0.1	1	0	0	12	3	LL
713	2	31	2	0	0	1	0	1	1	0	0	1	LL
714	2	21	1	0	1.83	2	0.5	1	1	0	2	1	SL
715	2	20	1	0	1	2	1.4	2	2	1	0	1	SL
716	2	20	1	1	0.67	1	0.3	1	0	0	0	1	SS
717	2	23	1	0	1.5	2	1.2	2	1	0	0	1	SL
718	1	19	1	0	0.83	1	0.6	1	1	0	0	1	LL
719	1	20	1	1	1	2	0.7	1	1	0	7	3	SS
720	2	21	1	0	1.5	2	0.6	1	2	1	3	1	SL
721	2	20	1	0	1.17	2	0.3	1	0	0	0	1	SL
722	1	21	1	0	1.17	2	1.5	2	0	0	10	3	SL
723	2	19	1	0	1	2	0.6	1	2	1	1	1	LL
724	2	21	1	1	0.67	1	0.2	1	0	0	0	1	LL
725	2	21	1	0	1.5	2	1.7	2	0	0	0	1	SL
726	2	21	1	1	0.5	1	1.7	2	0	0	3	1	SL
727	2	22	1	0	1.5	2	1	2	2	1	4	2	SL
728	1	21	1	0	2.33	3	1	2	0	0	1	1	SS
729	2	21	1	0	0.5	1	0.8	1	0	0	1	1	SL
730	2	20	1	0	1.17	2	0.3	1	2	1	2	1	SL
731	1	21	1	0	1.17	2	0.4	1	1	0	1	1	SS
732	2	20	1	0	1.33	2	0.3	1	0	0	2	1	SL
733	2	21	1	1	0.67	1	0.4	1	2	1	1	1	SL
734	2	#NULL!	#NULL!	0	1.5	2	0.7	1	2	1	0	1	SL
735	2	30	1	0	0.17	1	0	1	1	0	4	2	SS
736	2	20	1	0	0.5	1	0.1	1	2	1	2	1	SL
737	2	20	1	0	1.33	2	0.6	1	2	1	1	1	LL
738	2	20	1	0	1.17	2	0.1	1	2	1	1	1	SL
739	2	20	1	0	1.33	2	0.7	1	2	1	6	2	LL
740	2	20	1	0	1	2	0.3	1	1	0	0	1	SL
741	2	23	1	1	0.17	1	2.4	3	3	2	4	2	SS
742	2	21	1	1	1.17	2	1.3	2	2	1	2	1	SS
743	2	21	1	0	0.17	1	0.2	1	0	0	2	1	SL
744	2	22	1	0	0.67	1	0.1	1	0	0	0	1	SL
745	1	21	1	0	1.17	2	0.5	1	0	0	3	1	SS
746	2	21	1	0	0.33	1	0.2	1	0	0	0	1	LL
747	2	21	1	0	0	1	0	1	1	0	1	1	SL
748	2	20	1	0	2.33	3	1.2	2	2	1	3	1	SS
749	2	22	1	0	0.17	1	1	2	2	1	2	1	LL
750	2	21	1	1	0.83	1	2.5	3	1	0	4	2	LL
751	2	26	1	0	1.67	2	0.6	1	1	0	0	1	SS
752	2	23	1	1	0.83	1	1.6	2	2	1	0	1	SL
753	2	23	1	0	1.33	2	0.9	1	2	1	3	1	SL
754	2	21	1	0	1.17	2	1.9	2	1	0	2	1	LL
755	2	22	1	0	0.17	1	0.1	1	2	1	0	1	SL
756	2	21	1	0	0.5	1	0.4	1	2	1	2	1	SL
757	1	49	2	0	0	1	0.1	1	0	0	3	1	LL
758	2	26	1	0	1.17	2	0.5	1	4	2	3	1	LL
759	1	29	1	1	1.17	2	1.8	2	2	1	3	1	SL
760	2	#NULL!	#NULL!	0	0.67	1	0.1	1	0	0	0	1	SL
761	1	30	1	0	1.17	2	0.7	1	0	0	4	2	SL
762	1	41	2	0	0.33	1	0	1	1	0	2	1	SS
763	1	28	1	0	0.17	1	0.2	1	0	0	0	1	LL
764	1	31	2	0	1.33	2	1.8	2	4	2	0	1	SL

765	1	18	1	0	1	2	0.3	1	3	2	0	1	LL
766	2	42	2	0	1.5	2	1.5	2	0	0	6	2	SL
767	2	50	2	0	1.17	2	0.6	1	2	1	4	2	LL
768	2	28	1	1	1.33	2	1.3	2	1	0	2	1	SL
769	2	47	2	0	0	1	0	1	0	0	3	1	SL
770	1	48	2	0	0	1	0.3	1	0	0	0	1	LL
771	2	30	1	0	0.17	1	0.2	1	0	0	0	1	SS
772	2	41	2	0	1.83	2	0.6	1	2	1	2	1	SL
773	2	58	2	0	1.5	2	1.1	2	0	0	1	1	SL
774	2	51	2	0	2.5	3	1.6	2	1	0	1	1	SS
775	1	31	2	0	0.33	1	0.4	1	0	0	5	2	SL
776	1	34	2	0	0.67	1	0.8	1	2	1	3	1	SS
777	1	28	1	0	0.17	1	0.1	1	2	1	3	1	LL
778	1	29	1	0	0.33	1	0.4	1	1	0	10	3	SS
779	2	42	2	1	1.5	2	2.3	3	0	0	2	1	SS
780	2	31	2	1	0.5	1	0.3	1	2	1	0	1	SS
781	1	41	2	1	1.17	2	0.6	1	0	0	6	2	LL
782	2	48	2	1	2	3	3	3	1	0	5	2	LL
783	1	33	2	1	0	1	0.1	1	0	0	1	1	SL
784	1	41	2	0	0.17	1	0.1	1	0	0	3	1	LL
785	1	#NULL!	#NULL!	0	0	1	0	1	1	0	0	1	SS
786	1	32	2	0	0.17	1	0.1	1	0	0	0	1	SL
787	1	34	2	0	1.17	2	0.7	1	0	0	3	1	SL
788	1	#NULL!	#NULL!	0	0	1	0.1	1	1	0	0	1	SL
789	1	#NULL!	#NULL!	0	0.17	1	0.5	1	1	0	7	3	SS
790	1	29	1	0	0.67	1	0.4	1	0	0	1	1	SS
791	2	27	1	0	0.17	1	0.1	1	0	0	4	2	SL
792	1	24	1	1	2.5	3	2.7	3	1	0	2	1	SS
793	1	47	2	0	0.33	1	0.1	1	0	0	2	1	SS
794	1	36	2	1	0.67	1	2	3	2	1	5	2	SL
795	1	29	1	0	0.17	1	0.6	1	1	0	5	2	SS
796	1	29	1	0	0	1	0.9	1	1	0	2	1	SS
797	2	55	2	1	2.33	3	1.1	2	0	0	4	2	SL
798	1	29	1	0	0.17	1	0.1	1	2	1	3	1	SL
799	1	34	2	0	0	1	0.1	1	0	0	0	1	SL
800	1	24	1	0	0.67	1	0.4	1	0	0	0	1	SL
801	1	38	2	0	0	1	0	1	1	0	1	1	LL
802	1	32	2	0	1.33	2	0.4	1	0	0	3	1	LL
803	1	39	2	0	0.17	1	0	1	1	0	1	1	SS
804	1	34	2	0	0.17	1	0.1	1	0	0	2	1	SL
805	1	28	1	0	0.17	1	0.1	1	0	0	6	2	LL
806	1	39	2	0	0	1	0	1	2	1	9	3	SS
807	1	38	2	0	0	1	0	1	1	0	0	1	SL
808	1	31	2	0	0	1	0	1	0	0	1	1	SL
809	1	52	2	0	0.83	1	0.1	1	1	0	3	1	LL
810	1	33	2	0	0.33	1	0	1	0	0	2	1	SL
811	1	54	2	1	1.17	2	1.7	2	2	1	2	1	LL
812	2	33	2	1	0.17	1	0	1	1	0	1	1	LL
813	2	60	2	1	2.33	3	2.8	3	2	1	4	2	SL
814	1	60	2	1	1.6	2	1.6	2	0	0	8	3	LL
815	2	47	2	1	2.83	3	2.8	3	0	0	9	3	LL
816	2	31	2	1	0.83	1	0.4	1	3	2	6	2	SL
817	2	44	2	1	2.33	3	1.8	2	0	0	11	3	LL
818	2	43	2	1	0.67	1	1.3	2	2	1	2	1	LL
819	2	49	2	1	3	3	1.4	2	6	2	0	1	SS
820	2	37	2	1	2.17	3	1.6	2	3	2	1	1	SS
821	2	55	2	1	2.33	3	2.9	3	0	0	2	1	SS
822	1	#NULL!	#NULL!	0	0	1	0	1	0	0	1	1	SL
823	2	22	1	0	0.33	1	0.1	1	0	0	0	1	SS

824	1	29	1	0	0.17	1	0	1	2	1	1	1	SL
825	1	32	2	0	0	1	0.1	1	3	2	1	1	LL
826	1	32	2	1	0.67	1	0.3	1	0	0	1	1	SL
827	1	27	1	0	0	1	0.1	1	0	0	0	1	SL
828	1	33	2	0	0	1	0	1	0	0	1	1	SS
829	2	54	2	1	4	3	3.7	3	5	2	7	3	SL
830	2	22	1	1	1.17	2	1.6	2	0	0	4	2	SS
831	1	22	1	1	0	1	0.3	1	0	0	3	1	SS
832	2	41	2	1	0.67	1	0.4	1	3	2	6	2	SL
833	1	31	2	0	1.83	2	1.7	2	0	0	5	2	SL
834	1	40	2	1	0.33	1	0.1	1	0	0	2	1	LL
835	1	27	1	0	0.33	1	0.3	1	1	0	2	1	SL
836	1	30	1	0	0	1	0.1	1	1	0	4	2	LL
837	1	25	1	0	0	1	0.3	1	0	0	0	1	SS
838	1	27	1	0	0.17	1	0.4	1	1	0	1	1	SL
839	1	28	1	0	0.5	1	0.1	1	1	0	0	1	SL
840	1	36	2	0	3.67	3	3.8	3	0	0	1	1	SL
841	2	48	2	1	2	3	0.7	1	0	0	0	1	SL
842	2	49	2	1	2	3	2.6	3	2	1	12	3	SS
843	2	53	2	1	1.83	2	1.7	2	2	1	12	3	SL
844	1	28	1	1	2.83	3	4	3	6	2	13	3	SS
845	2	#NULL!	#NULL!	1	0	1	0	1	2	1	1	1	SL
846	1	35	2	0	0.83	1	0.1	1	1	0	2	1	LL
847	1	33	2	0	0.5	1	0.4	1	1	0	2	1	LL
848	1	54	2	0	0.17	1	0.2	1	2	1	2	1	SL
849	1	#NULL!	#NULL!	0	1.17	2	0.7	1	2	1	9	3	SL
850	1	34	2	0	0.67	1	0.4	1	0	0	4	2	LL
851	1	#NULL!	#NULL!	0	0	1	0	1	0	0	7	3	SL
852	1	60	2	0	0	1	0.1	1	1	0	0	1	LL
853	1	34	2	0	0.17	1	0.1	1	0	0	1	1	LL
854	1	48	2	1	0.83	1	0.4	1	1	0	2	1	SS
855	1	26	1	0	0.33	1	0.3	1	2	1	3	1	SL
856	2	24	1	0	0	1	0	1	0	0	1	1	SL
857	1	45	2	0	0.83	1	1	2	1	0	3	1	LL
858	1	37	2	0	2.17	3	2	3	3	2	3	1	SS
859	2	58	2	0	1.17	2	0.8	1	3	2	0	1	SS
860	1	39	2	0	0	1	0	1	1	0	0	1	SS
861	2	42	2	1	3.17	3	1.7	2	5	2	6	2	LL
862	2	52	2	0	0.33	1	1	2	0	0	2	1	SL
863	1	38	2	0	0.17	1	2	3	0	0	2	1	LL
864	1	27	1	0	0.67	1	0.4	1	4	2	6	2	SL
865	2	39	2	0	0.17	1	0	1	1	0	1	1	SL
866	1	28	1	0	0	1	0	1	0	0	1	1	LL
867	2	28	1	1	0.17	1	0.2	1	3	2	3	1	SL
868	1	33	2	1	0.67	1	0.2	1	1	0	1	1	LL
869	1	28	1	0	0	1	0.4	1	1	0	0	1	LL
870	1	33	2	0	0.33	1	0.2	1	1	0	5	2	SL
871	1	39	2	1	0	1	0	1	0	0	6	2	LL
872	1	26	1	1	0.17	1	0.2	1	1	0	3	1	SS
873	2	28	1	0	1.17	2	0	1	2	1	6	2	SL
874	1	31	2	0	0.17	1	0	1	0	0	1	1	LL
875	1	28	1	0	0.17	1	0.1	1	1	0	0	1	SS
876	1	21	1	0	0.5	1	0.3	1	1	0	1	1	SL
877	2	60	2	0	0.33	1	1.2	2	3	2	4	2	SL
878	1	21	1	0	0.17	1	1.6	2	1	0	6	2	SL
879	1	28	1	0	0.5	1	0.3	1	2	1	1	1	SL
880	1	23	1	0	0.17	1	0.4	1	2	1	1	1	SL
881	2	23	1	0	0	1	0.2	1	1	0	2	1	SL
882	1	26	1	0	0.67	1	0.9	1	3	2	3	1	SL

883	2	25	1	0	0.33	1	0.2	1	0	0	4	2	SL
884	1	40	2	0	0.33	1	0.2	1	0	0	1	1	SL
885	1	#NULL!	#NULL!	0	0.33	1	0.3	1	0	0	2	1	SS
886	1	39	2	1	0.67	1	0.1	1	1	0	8	3	SS
887	1	33	2	1	0	1	0	1	0	0	9	3	SL
888	1	41	2	0	0.33	1	0	1	1	0	4	2	SL
889	1	31	2	0	0	1	0	1	0	0	2	1	SL
890	1	#NULL!	#NULL!	0	0.33	1	0.6	1	3	2	3	1	SL
891	1	22	1	0	0.17	1	0	1	3	2	4	2	LL
892	1	24	1	0	1.5	2	1.7	2	2	1	6	2	LL
893	1	41	2	0	1	2	1.1	2	1	0	5	2	LL
894	1	#NULL!	#NULL!	0	0.5	1	0.1	1	2	1	2	1	LL
895	2	34	2	1	3.17	3	2.4	3	6	2	3	1	SL
896	1	30	1	0	0.5	1	0.2	1	0	0	1	1	SS
897	1	26	1	0	1	2	0.5	1	3	2	2	1	SL
898	1	32	2	0	0.17	1	0.1	1	2	1	2	1	LL
899	1	25	1	0	0.67	1	0.7	1	3	2	1	1	SL
900	1	51	2	0	0.33	1	0.4	1	0	0	0	1	SS
901	1	30	1	0	0.33	1	0	1	0	0	0	1	LL
902	1	27	1	0	0.33	1	1.3	2	1	0	2	1	LL
903	1	19	1	0	0.33	1	0.9	1	2	1	2	1	LL
904	1	59	2	0	0.17	1	0	1	2	1	2	1	SL
905	1	22	1	0	0.5	1	0.8	1	2	1	1	1	SL
906	1	32	2	0	0.33	1	0	1	0	0	8	3	SS
907	1	28	1	0	0.5	1	0.5	1	2	1	0	1	SL
908	1	46	2	0	0.17	1	0.2	1	2	1	2	1	SL
909	1	38	2	0	0.17	1	0.2	1	1	0	3	1	SS
910	1	30	1	0	0.67	1	0.2	1	1	0	2	1	SL
911	1	55	2	0	0.83	1	0.2	1	0	0	1	1	LL
912	1	53	2	0	0.17	1	0.1	1	1	0	4	2	SL
913	1	47	2	0	0.17	1	0	1	1	0	1	1	SL
914	1	22	1	1	0.33	1	0.1	1	1	0	0	1	LL
915	1	31	2	0	0.83	1	0.7	1	1	0	8	3	LL
916	2	23	1	0	0.5	1	0.2	1	0	0	0	1	LL
917	1	24	1	0	0.17	1	0.5	1	1	0	1	1	SS
918	2	22	1	0	1.5	2	0.6	1	1	0	1	1	SL
919	2	22	1	0	0.5	1	0.9	1	0	0	3	1	LL
920	1	22	1	0	1.33	2	0.2	1	5	2	2	1	SL
921	2	23	1	0	0.67	1	0.5	1	0	0	2	1	SL
922	1	22	1	0	0.33	1	0	1	0	0	0	1	SL
923	2	21	1	0	0.33	1	0.3	1	3	2	0	1	SS
924	2	23	1	1	0	1	0.6	1	2	1	3	1	SL
925	1	20	1	0	0.5	1	0.1	1	2	1	1	1	SL
926	2	48	2	0	0.5	1	0.1	1	0	0	1	1	SL
927	1	22	1	0	0	1	0.2	1	2	1	4	2	LL
928	1	#NULL!	#NULL!	0	0	1	0.1	1	1	0	2	1	SS
929	2	24	1	0	0	1	0.6	1	0	0	1	1	SL
930	1	35	2	0	0.33	1	0	1	1	0	2	1	SS
931	1	46	2	0	0	1	0	1	1	0	2	1	LL
932	1	27	1	0	0	1	0	1	1	0	0	1	LL
933	1	28	1	0	0	1	0.2	1	1	0	0	1	SS
934	1	26	1	0	0.67	1	0.4	1	0	0	2	1	SL
935	1	30	1	0	0	1	0.1	1	0	0	3	1	SL
936	1	26	1	0	0	1	0	1	1	0	1	1	SL
937	1	40	2	0	0.17	1	0	1	0	0	2	1	SL
938	1	31	2	0	0	1	0	1	0	0	0	1	SS
939	1	#NULL!	#NULL!	0	0.17	1	0.4	1	1	0	0	1	LL
940	1	32	2	0	0.17	1	0	1	2	1	0	1	SL
941	1	32	2	0	0.33	1	0.1	1	0	0	5	2	SS

942	2	30	1	0	0	1	0	1	0	0	10	3	LL
943	2	25	1	0	0.17	1	0.4	1	1	0	0	1	SS
944	1	39	2	1	0.67	1	0.2	1	0	0	4	2	SL
945	1	24	1	0	0.17	1	0.6	1	0	0	4	2	SL
946	1	39	2	0	0.67	1	0.3	1	1	0	4	2	LL
947	1	22	1	0	0.67	1	0.7	1	2	1	3	1	SL
948	1	27	1	0	0	1	0.2	1	0	0	3	1	SL
949	1	23	1	0	0	1	0	1	2	1	0	1	SL
950	2	26	1	0	1.33	2	0.2	1	1	0	5	2	SS
951	1	26	1	0	0.83	1	0.4	1	0	0	2	1	SL
952	2	#NULL!	#NULL!	0	1	2	0.7	1	1	0	3	1	LL
953	1	26	1	0	0.17	1	0	1	1	0	2	1	LL
954	1	#NULL!	#NULL!	0	0	1	0	1	0	0	1	1	SL
955	1	#NULL!	#NULL!	0	0	1	0	1	2	1	2	1	LL
956	2	39	2	0	0.5	1	0.2	1	0	0	2	1	SL
957	1	31	2	1	1.33	2	0.5	1	2	1	2	1	LL
958	2	30	1	0	0.67	1	0.5	1	3	2	8	3	LL
959	1	39	2	1	1.33	2	1.6	2	2	1	6	2	LL
960	1	28	1	0	0	1	0	1	0	0	0	1	SS
961	1	25	1	0	0	1	0.4	1	2	1	2	1	LL
962	2	26	1	0	1.17	2	0.4	1	1	0	2	1	SS
963	1	#NULL!	#NULL!	0	0	1	0.1	1	1	0	9	3	LL
964	2	37	2	0	1.33	2	1.5	2	1	0	9	3	SS
965	1	33	2	0	0.5	1	0.1	1	0	0	4	2	SS
966	1	#NULL!	#NULL!	0	0	1	0	1	0	0	1	1	SL
967	1	38	2	0	1.33	2	2.4	3	2	1	13	3	LL
968	1	34	2	0	0	1	0	1	1	0	1	1	SL
969	1	#NULL!	#NULL!	0	0	1	0	1	0	0	2	1	SL
970	1	28	1	0	0.17	1	0.3	1	1	0	10	3	LL
971	1	28	1	0	0	1	0	1	0	0	8	3	SS
972	1	29	1	0	0	1	0	1	1	0	0	1	LL
973	2	26	1	0	0.17	1	0.1	1	1	0	0	1	SL
974	2	27	1	0	0.17	1	0	1	1	0	2	1	SL
975	2	41	2	0	0	1	0	1	1	0	1	1	LL
976	1	34	2	0	0	1	0	1	0	0	3	1	SS
977	1	27	1	1	0.17	1	0.4	1	0	0	2	1	LL
978	1	#NULL!	#NULL!	0	0	1	0	1	3	2	1	1	SL
979	1	#NULL!	#NULL!	0	0.5	1	0.1	1	2	1	1	1	SL
980	2	39	2	0	0.67	1	0.1	1	0	0	0	1	SS
981	2	37	2	0	0.17	1	0.1	1	0	0	0	1	SL
982	2	34	2	0	0.17	1	0.1	1	1	0	1	1	SL
983	1	28	1	0	0	1	0	1	0	0	2	1	LL
984	2	31	2	0	0	1	0	1	0	0	0	1	SS
985	1	37	2	0	0	1	0	1	1	0	0	1	LL
986	1	29	1	0	0.2	1	0.3	1	0	0	10	3	SL
987	1	26	1	0	0	1	0	1	2	1	4	2	LL
988	1	43	2	1	1.83	2	0.8	1	2	1	2	1	SL
989	2	36	2	0	0.17	1	0.1	1	2	1	1	1	SL
990	2	#NULL!	#NULL!	1	1.67	2	1	2	3	2	2	1	SS
991	1	35	2	0	#NULL!	#NULL!	#NULL!	#NULL!	0	0	2	1	SL
992	1	22	1	0	0.5	1	0.9	1	0	0	2	1	SL
993	1	32	2	0	0.17	1	0	1	1	0	1	1	SL
994	1	29	1	0	0.5	1	0.3	1	1	0	4	2	SS
995	2	26	1	1	0	1	0	1	2	1	7	3	SL
996	2	20	1	0	0	1	0.1	1	1	0	2	1	LL
997	1	30	1	0	0	1	0	1	0	0	1	1	LL
998	1	26	1	0	1	2	0.9	1	1	0	3	1	SL
999	1	31	2	0	0.5	1	0.8	1	1	0	2	1	LL
1000	1	29	1	0	0.33	1	0.1	1	2	1	3	1	LL

1001	1	23	1	0	0.17	1	0.4	1	6	2	1	1	LL
1002	1	25	1	0	0.33	1	0.1	1	1	0	2	1	SL
1003	2	41	2	0	0	1	0	1	0	0	0	1	SL
1004	2	55	2	1	1.33	2	0.3	1	2	1	2	1	SS
1005	2	40	2	1	1.17	2	0.8	1	0	0	#NULL!	#NULL!	LL
1006	2	56	2	1	2.67	3	3	3	1	0	10	3	SL
1007	2	46	2	0	0.67	1	0.2	1	0	0	0	1	LL
1008	2	34	2	1	2.5	3	2.1	3	0	0	4	2	LL
1009	2	24	1	0	0	1	0.2	1	1	0	0	1	SS
1010	1	38	2	1	0.17	1	0.7	1	1	0	1	1	LL
1011	2	25	1	0	1.5	2	1.1	2	0	0	0	1	SL
1012	2	49	2	0	1.5	2	1.4	2	1	0	8	3	SL
1013	2	45	2	0	0.33	1	0.1	1	0	0	7	3	LL
1014	2	22	1	0	0.83	1	0.9	1	3	2	0	1	LL
1015	2	23	1	1	2.5	3	2.8	3	2	1	5	2	SL
1016	1	29	1	0	0	1	0.1	1	0	0	6	2	SL
1017	2	28	1	0	0.67	1	0.5	1	0	0	1	1	SL
1018	1	45	2	0	0.17	1	0.4	1	1	0	8	3	SS
1019	2	46	2	1	0.33	1	0.6	1	1	0	2	1	SL
1020	1	40	2	0	0.33	1	0.3	1	3	2	1	1	SS
1021	1	49	2	0	0	1	0.1	1	0	0	1	1	SS
1022	2	37	2	1	0.33	1	0.1	1	0	0	0	1	LL
1023	2	46	2	0	1	2	0.6	1	0	0	0	1	SL
1024	2	21	1	1	4	3	3.9	3	1	0	0	1	SL
1025	2	50	2	0	0	1	0.1	1	0	0	0	1	LL
1026	2	39	2	1	1.5	2	0.5	1	2	1	7	3	LL
1027	2	35	2	1	1.33	2	0.8	1	3	2	1	1	SS
1028	1	35	2	0	1	2	0	1	2	1	9	3	SL
1029	1	45	2	0	0.5	1	0.2	1	0	0	6	2	SL
1030	2	32	2	1	0.83	1	0.7	1	1	0	3	1	SS
1031	2	29	1	0	0	1	0.1	1	1	0	1	1	SS
1032	2	37	2	1	0.17	1	0.1	1	0	0	0	1	SL
1033	2	47	2	1	0.17	1	0.3	1	0	0	12	3	SS
1034	2	45	2	0	0.33	1	0.1	1	0	0	6	2	SS
1035	2	43	2	0	1.67	2	1.7	2	2	1	9	3	LL
1036	2	36	2	0	2.17	3	0.8	1	0	0	9	3	LL
1037	2	28	1	1	0.33	1	0.2	1	0	0	1	1	SL
1038	2	33	2	1	0	1	0.2	1	0	0	2	1	SL
1039	1	29	1	0	0.5	1	0.6	1	1	0	0	1	LL
1040	2	26	1	1	1.67	2	1.5	2	0	0	0	1	LL
1041	1	46	2	0	0.17	1	0.3	1	1	0	6	2	SL
1042	2	45	2	1	2.5	3	1.7	2	2	1	4	2	SL
1043	2	38	2	0	0.67	1	0.7	1	2	1	1	1	SL
1044	2	36	2	0	0.17	1	0.2	1	0	0	2	1	SS
1045	1	49	2	0	0	1	0	1	2	1	3	1	LL
1046	2	48	2	0	0.67	1	0.4	1	1	0	1	1	LL
1047	2	47	2	1	0.5	1	0.9	1	1	0	2	1	SS
1048	2	44	2	1	2.83	3	1.8	2	0	0	7	3	SL
1049	2	36	2	0	0.17	1	0.8	1	1	0	0	1	SL
1050	2	35	2	0	0	1	0	1	2	1	6	2	SL
1051	2	35	2	0	0.17	1	0.1	1	1	0	0	1	LL
1052	2	45	2	0	1	2	0.6	1	4	2	2	1	SL
1053	2	30	1	0	0.33	1	0.2	1	1	0	1	1	SL
1054	2	40	2	0	0.67	1	0.4	1	1	0	0	1	SL
1055	2	31	2	0	0.5	1	0.2	1	2	1	2	1	SS
1056	2	43	2	1	1.5	2	0.7	1	4	2	2	1	SL
1057	2	24	1	1	0.83	1	0.7	1	1	0	5	2	SL
1058	2	49	2	1	0.33	1	1.9	2	0	0	10	3	SL
1059	1	36	2	1	1	2	0.8	1	0	0	5	2	SL

1060	1	37	2	0	0.33	1	0.4	1	1	0	0	1	SL
1061	2	26	1	0	0.67	1	0.7	1	0	0	1	1	SL
1062	1	23	1	0	0.17	1	0.1	1	1	0	2	1	SL
1063	2	37	2	1	2.5	3	2.8	3	0	0	6	2	LL
1064	1	28	1	1	1.5	2	2	3	1	0	2	1	SL
1065	1	40	2	0	0.67	1	0.4	1	0	0	6	2	SL
1066	1	40	2	0	0.17	1	0.1	1	1	0	0	1	SL
1067	2	32	2	0	0.17	1	0	1	1	0	3	1	SL
1068	2	29	1	0	0	1	0	1	1	0	0	1	SL
1069	1	43	2	1	0.33	1	0.4	1	1	0	1	1	LL
1070	2	48	2	1	0	1	0	1	0	0	2	1	SS
1071	2	18	1	0	0.33	1	0	1	1	0	0	1	SL
1072	2	45	2	0	0.17	1	0.2	1	1	0	0	1	SS
1073	2	29	1	0	0	1	0.1	1	2	1	0	1	SS
1074	1	41	2	1	0	1	0	1	0	0	3	1	SL
1075	1	35	2	1	1.5	2	1.6	2	0	0	1	1	SL
1076	2	46	2	0	0.17	1	0.1	1	2	1	0	1	SL
1077	2	37	2	0	0.33	1	0.4	1	0	0	10	3	LL
1078	2	34	2	0	0.17	1	0	1	2	1	1	1	LL
1079	2	34	2	1	0.17	1	0.5	1	0	0	7	3	SL
1080	2	35	2	0	0.17	1	0	1	1	0	0	1	SS
1081	2	37	2	0	0.5	1	0.6	1	1	0	3	1	SL
1082	2	45	2	1	1.17	2	0.8	1	0	0	0	1	SL
1083	2	47	2	0	0.67	1	0	1	0	0	2	1	SL
1084	2	42	2	0	0	1	0	1	0	0	2	1	SL
1085	2	24	1	1	0.83	1	0.5	1	1	0	7	3	LL
1086	2	30	1	1	0	1	0.1	1	1	0	0	1	SS
1087	2	46	2	0	0.33	1	0.2	1	2	1	4	2	SL
1088	2	49	2	0	0.67	1	0.1	1	1	0	0	1	LL
1089	2	37	2	0	0.17	1	0.9	1	2	1	2	1	LL
1090	2	36	2	0	0	1	0.1	1	0	0	2	1	SS
1091	1	46	2	1	3.33	3	3.1	3	1	0	12	3	SL
1092	2	42	2	1	0.5	1	0.4	1	2	1	0	1	SS
1093	1	38	2	1	1	2	1.3	2	1	0	7	3	LL
1094	1	40	2	0	0.5	1	0.3	1	2	1	1	1	LL
1095	2	30	1	0	0.33	1	0	1	1	0	0	1	SL
1096	2	22	1	1	1.33	2	1	2	0	0	0	1	SL
1097	2	34	2	0	0	1	0	1	0	0	0	1	SL
1098	2	38	2	0	0.17	1	0.2	1	3	2	0	1	SL
1099	2	44	2	0	0	1	0	1	2	1	1	1	SL
1100	2	32	2	0	0	1	0.1	1	0	0	0	1	LL
1101	2	31	2	0	0.17	1	0.1	1	0	0	1	1	SL
1102	1	46	2	1	0.33	1	0.9	1	2	1	3	1	SL
1103	2	38	2	1	0.17	1	0.4	1	1	0	2	1	SL
1104	2	38	2	1	2.83	3	2.2	3	2	1	6	2	LL
1105	2	38	2	1	0.33	1	0.2	1	0	0	0	1	LL
1106	2	38	2	1	1.17	2	1.5	2	0	0	7	3	SL
1107	1	19	1	0	0	1	0.1	1	1	0	0	1	SL
1108	2	26	1	0	0.17	1	0.1	1	0	0	8	3	SS
1109	2	30	1	0	0.33	1	0.6	1	0	0	0	1	SS
1110	2	35	2	1	2	3	1.3	2	0	0	0	1	SL
1111	1	47	2	1	0.5	1	0.6	1	2	1	3	1	SL
1112	2	33	2	0	0.5	1	0.5	1	0	0	6	2	SS
1113	2	29	1	0	0.5	1	0.2	1	0	0	1	1	LL
1114	2	19	1	0	1	2	0.5	1	1	0	0	1	SL
1115	2	43	2	1	1.83	2	2.1	3	2	1	7	3	SL
1116	2	40	2	1	0	1	0	1	0	0	0	1	SS
1117	2	43	2	0	0.5	1	0.9	1	1	0	3	1	LL
1118	1	18	1	0	0.33	1	0.4	1	1	0	1	1	SS

1119	2	30	1	1	3	3	3.1	3	2	1	8	3	SS
1120	2	30	1	0	0.17	1	0.5	1	1	0	0	1	SL
1121	2	34	2	0	0.33	1	0.4	1	1	0	0	1	LL
1122	2	42	2	1	0	1	1.2	2	3	2	9	3	SS
1123	2	42	2	1	0.67	1	0.9	1	0	0	5	2	SL
1124	1	40	2	0	0.33	1	0.2	1	0	0	0	1	SS
1125	2	38	2	1	0.33	1	0.4	1	1	0	0	1	SL
1126	2	38	2	0	0	1	0	1	1	0	3	1	LL
1127	1	34	2	0	0	1	0.2	1	0	0	1	1	SS
1128	2	43	2	0	0	1	0.5	1	1	0	1	1	SL
1129	2	39	2	0	2.17	3	1.8	2	0	0	0	1	SL
1130	2	47	2	1	1.67	2	2	3	0	0	1	1	LL
1131	1	43	2	1	0.2	1	1	2	2	1	5	2	LL
1132	1	48	2	1	0.17	1	1.9	2	1	0	2	1	SS
1133	2	38	2	0	0.17	1	0	1	0	0	0	1	SL
1134	2	38	2	1	0.33	1	0.9	1	4	2	6	2	LL
1135	1	37	2	0	0	1	0	1	1	0	3	1	SL
1136	2	49	2	1	0.17	1	0.4	1	1	0	4	2	LL
1137	2	47	2	0	0.33	1	0.2	1	0	0	6	2	LL
1138	2	36	2	1	1.67	2	0.9	1	2	1	9	3	SL
1139	1	32	2	1	0.33	1	0.5	1	0	0	2	1	SL
1140	2	26	1	0	0.33	1	1	2	1	0	0	1	SL
1141	1	35	2	0	0	1	0.5	1	3	2	4	2	SL
1142	2	38	2	1	0.17	1	0.1	1	0	0	2	1	LL
1143	2	45	2	0	0.17	1	0.1	1	0	0	0	1	LL
1144	2	47	2	1	0.5	1	0.1	1	0	0	8	3	SL
1145	1	36	2	0	2.33	3	2.3	3	0	0	4	2	LL
1146	2	37	2	0	0.67	1	0.8	1	0	0	8	3	SL
1147	1	49	2	0	0.33	1	0.1	1	0	0	3	1	SL
1148	2	36	2	1	0.67	1	0.7	1	1	0	5	2	LL
1149	2	43	2	0	0.33	1	0	1	1	0	0	1	SL
1150	1	32	2	1	2	3	1.4	2	2	1	12	3	LL
1151	2	33	2	0	0.17	1	0.1	1	1	0	0	1	SS
1152	2	34	2	0	0.83	1	0.3	1	1	0	0	1	LL
1153	2	23	1	0	0.17	1	0.2	1	0	0	3	1	SL
1154	1	41	2	0	0	1	0	1	0	0	2	1	LL
1155	1	40	2	1	0.83	1	1.4	2	1	0	7	3	LL
1156	2	49	2	1	0.33	1	0.5	1	0	0	0	1	SS
1157	2	47	2	0	0.17	1	0.4	1	0	0	5	2	SL
1158	2	30	1	1	0.5	1	0.6	1	2	1	1	1	SS
1159	2	36	2	1	0.33	1	0.3	1	2	1	2	1	SS
1160	2	49	2	0	0.5	1	0.6	1	2	1	2	1	SL
1161	1	28	1	0	1.33	2	1.5	2	4	2	14	3	SL
1162	1	27	1	0	0.5	1	0	1	0	0	2	1	SL
1163	2	42	2	1	0.17	1	0.2	1	0	0	1	1	SL
1164	2	34	2	0	1	2	0.4	1	1	0	4	2	SS
1165	2	27	1	1	0.33	1	0.9	1	2	1	0	1	LL
1166	2	23	1	0	2.83	3	0.9	1	3	2	0	1	LL
1167	2	19	1	1	0.67	1	1	2	0	0	3	1	LL
1168	1	45	2	0	1.5	2	1.1	2	1	0	1	1	SS
1169	2	33	2	1	0.5	1	1.2	2	2	1	5	2	SL
1170	2	42	2	0	0.17	1	0.1	1	0	0	3	1	SS
1171	2	43	2	1	0.17	1	0.5	1	2	1	1	1	SS
1172	2	36	2	1	0.17	1	0.3	1	0	0	2	1	SL
1173	1	43	2	1	2.67	3	2.8	3	1	0	1	1	SL
1174	1	42	2	0	0.33	1	0.3	1	1	0	6	2	SS
1175	2	39	2	0	0	1	0.1	1	0	0	1	1	LL
1176	1	40	2	1	0.17	1	0	1	1	0	0	1	SS
1177	2	27	1	0	0.33	1	0.2	1	2	1	2	1	SS

1178	2	21	1	0	0.17	1	0.2	1	0	0	0	1	SS
1179	2	25	1	0	0.83	1	0.7	1	0	0	2	1	LL
1180	1	20	1	0	0	1	0.1	1	2	1	1	1	SL
1181	2	26	1	0	0	1	0	1	0	0	4	2	LL
1182	2	39	2	0	1	2	0.7	1	0	0	5	2	LL
1183	2	36	2	0	0.33	1	0.7	1	0	0	3	1	LL
1184	2	45	2	0	0	1	0.2	1	0	0	4	2	SL
1185	2	36	2	0	0.17	1	0.3	1	3	2	0	1	SL
1186	1	41	2	0	0.33	1	0.3	1	1	0	2	1	SL
1187	2	41	2	1	1.5	2	1.5	2	2	1	3	1	SL
1188	1	45	2	0	0	1	0.1	1	0	0	3	1	LL
1189	2	41	2	0	2.17	3	1.9	2	0	0	6	2	SL
1190	2	43	2	1	0	1	0.2	1	1	0	3	1	SL
1191	2	45	2	1	0.33	1	0	1	1	0	2	1	SL
1192	2	49	2	0	0	1	0	1	0	0	6	2	SS
1193	2	43	2	1	0.33	1	0.8	1	2	1	3	1	SS
1194	1	40	2	1	1.17	2	1.8	2	3	2	3	1	SS
1195	2	39	2	0	0	1	0.1	1	0	0	2	1	LL
1196	2	46	2	0	3.33	3	3.9	3	0	0	1	1	SL
1197	2	42	2	0	1.67	2	0.5	1	5	2	6	2	SL
1198	2	37	2	1	0	1	1	2	1	0	3	1	LL
1199	2	31	2	0	0.67	1	0.9	1	1	0	1	1	LL
1200	2	25	1	0	1	2	0.8	1	1	0	0	1	SS
1201	2	36	2	0	0	1	0.1	1	0	0	0	1	SS
1202	2	22	1	0	0.67	1	0.5	1	2	1	0	1	SS
1203	1	22	1	0	0	1	0.1	1	2	1	0	1	LL
1204	2	23	1	0	0.33	1	0.1	1	5	2	0	1	LL
1205	2	19	1	0	1	2	0.7	1	1	0	7	3	SL
1206	2	24	1	0	0	1	0.3	1	1	0	6	2	SL
1207	1	36	2	0	0.5	1	0.9	1	0	0	2	1	LL
1208	1	19	1	0	0.33	1	0.8	1	1	0	2	1	SL
1209	2	35	2	1	1.33	2	1.3	2	1	0	2	1	SL
1210	1	38	2	0	0.17	1	0.1	1	0	0	0	1	SL
1211	2	48	2	0	1.5	2	0.6	1	1	0	3	1	LL
1212	2	34	2	0	0	1	0.3	1	0	0	5	2	LL
1213	2	44	2	1	2.5	3	2.3	3	1	0	11	3	SS
1214	2	34	2	0	0.33	1	0.1	1	2	1	0	1	LL
1215	2	36	2	0	0.17	1	0.1	1	1	0	6	2	LL
1216	2	24	1	0	0.17	1	0.9	1	1	0	4	2	LL
1217	1	42	2	0	0	1	0	1	0	0	1	1	LL
1218	2	29	1	1	0	1	0	1	1	0	8	3	SL
1219	1	46	2	0	0	1	0	1	3	2	2	1	SL
1220	1	43	2	1	0	1	0.4	1	0	0	1	1	SL
1221	2	30	1	0	0	1	0	1	0	0	2	1	SL
1222	1	48	2	1	1.83	2	0.6	1	0	0	5	2	LL
1223	2	49	2	0	0.33	1	0.2	1	1	0	2	1	SS
1224	2	27	1	1	1.17	2	1.5	2	0	0	1	1	SS
1225	2	21	1	0	0.5	1	0.6	1	1	0	0	1	SS
1226	1	36	2	0	0.17	1	0.7	1	1	0	2	1	LL
1227	2	42	2	1	0.33	1	0	1	1	0	0	1	SL
1228	2	50	2	1	3	3	2.8	3	1	0	10	3	SL
1229	1	47	2	0	0	1	0.1	1	1	0	8	3	LL
1230	2	20	1	1	0.83	1	2.2	3	0	0	1	1	LL
1231	1	26	1	1	0	1	0.2	1	0	0	11	3	LL
1232	1	28	1	0	0.17	1	0.4	1	1	0	1	1	SL
1233	2	28	1	0	0.83	1	1.3	2	1	0	0	1	SL
1234	1	26	1	0	0	1	0.4	1	0	0	3	1	SS
1235	1	24	1	1	0.17	1	0.3	1	0	0	2	1	SL
1236	2	43	2	0	0	1	0	1	3	2	0	1	LL

1237	1	30	1	0	0.33	1	0.6	1	2	1	1	1	LL
1238	1	43	2	0	0.17	1	0.1	1	1	0	2	1	LL
1239	2	42	2	0	0.17	1	0.1	1	0	0	1	1	SL
1240	2	49	2	1	1	2	0.7	1	2	1	1	1	SS
1241	2	34	2	1	0.17	1	0	1	0	0	0	1	SL
1242	2	39	2	0	0	1	0	1	0	0	0	1	LL
1243	2	32	2	1	0.67	1	0.8	1	0	0	2	1	SL
1244	2	38	2	1	2.5	3	2.8	3	0	0	13	3	LL
1245	2	29	1	0	0.5	1	0.1	1	3	2	0	1	SS
1246	2	19	1	1	1.33	2	2	3	3	2	14	3	SS
1247	2	19	1	1	3	3	3	3	0	0	5	2	LL
1248	2	25	1	1	1.5	2	1.2	2	1	0	0	1	LL
1249	1	49	2	1	0.17	1	0	1	1	0	2	1	SL
1250	2	38	2	1	0.17	1	0.5	1	0	0	4	2	LL
1251	2	32	2	1	2.17	3	1.3	2	2	1	5	2	LL
1252	2	34	2	1	0.33	1	0.5	1	4	2	10	3	SL
1253	1	26	1	1	0.33	1	0.1	1	0	0	3	1	LL
1254	1	27	1	1	1	2	2.1	3	0	0	0	1	LL
1255	2	21	1	1	1	2	0.5	1	3	2	1	1	LL
1256	1	38	2	1	1.5	2	2	3	1	0	10	3	SL
1257	1	24	1	0	0	1	0.1	1	1	0	1	1	SS
1258	1	28	1	0	0	1	0.3	1	0	0	2	1	SL
1259	2	21	1	1	0.67	1	1	2	0	0	3	1	SL
1260	2	19	1	0	0.17	1	0.6	1	0	0	5	2	SL
1261	2	29	1	0	2.17	3	0.8	1	6	2	5	2	SL
1262	1	22	1	0	0	1	0	1	2	1	0	1	SL
1263	2	23	1	0	0	1	0	1	1	0	0	1	SL
1264	2	48	2	0	0.17	1	0	1	0	0	5	2	LL
1265	2	23	1	0	0	1	0.3	1	1	0	3	1	LL
1266	1	46	2	1	1	2	0.5	1	0	0	1	1	SS
1267	2	23	1	1	3.83	3	3.3	3	2	1	2	1	LL
1268	2	40	2	1	0.2	1	1	2	1	0	0	1	SL
1269	2	33	2	1	0	1	0.3	1	1	0	2	1	SL
1270	2	37	2	0	0.17	1	0	1	0	0	3	1	LL
1271	2	20	1	1	0.17	1	0.4	1	3	2	1	1	SL
1272	2	34	2	0	0.17	1	0.1	1	0	0	3	1	SL
1273	2	26	1	1	0.67	1	0.3	1	0	0	0	1	SL
1274	2	39	2	1	2	3	1.8	2	2	1	8	3	LL
1275	1	26	1	1	0.33	1	0.8	1	1	0	4	2	SS
1276	1	33	2	1	0.17	1	0.7	1	1	0	2	1	SL
1277	2	40	2	1	0.33	1	0.4	1	4	2	0	1	SL
1278	1	33	2	0	0.83	1	1.4	2	2	1	3	1	LL
1279	2	30	1	0	0	1	0.1	1	2	1	7	3	LL
1280	1	39	2	1	0.33	1	0.4	1	2	1	1	1	SS
1281	2	21	1	1	0.67	1	0.8	1	2	1	0	1	SL
1282	2	29	1	1	0.83	1	0.9	1	3	2	3	1	LL
1283	2	31	2	0	0.33	1	0	1	1	0	0	1	SL
1284	2	45	2	0	0.83	1	0.4	1	0	0	0	1	LL
1285	2	43	2	0	0.33	1	0.4	1	0	0	2	1	LL
1286	1	39	2	1	3.5	3	3.5	3	3	2	4	2	SL
1287	1	40	2	1	1.5	2	0.6	1	3	2	3	1	SL
1288	2	33	2	0	0.67	1	0	1	0	0	2	1	LL
1289	1	35	2	1	0.5	1	1.3	2	0	0	3	1	SL
1290	2	37	2	1	1	2	0.9	1	0	0	3	1	SS
1291	1	37	2	0	0.33	1	0.3	1	0	0	1	1	LL
1292	2	40	2	0	0	1	0.5	1	2	1	2	1	LL
1293	2	36	2	0	0	1	0	1	0	0	1	1	SL
1294	2	21	1	1	0.5	1	0.5	1	1	0	8	3	SL
1295	2	42	2	0	0.33	1	0.2	1	0	0	2	1	SL

1296	2	44	2	0	0	1	0.1	1	0	0	0	1	SS
1297	1	46	2	0	0	1	0.3	1	2	1	2	1	SS
1298	2	38	2	1	3	3	2.1	3	0	0	3	1	LL
1299	2	28	1	1	2.67	3	2.9	3	0	0	11	3	SS
1300	2	19	1	1	0	1	0.4	1	0	0	2	1	SS
1301	2	20	1	1	4	3	3.3	3	7	2	#NULL!	#NULL!	SL
1302	1	23	1	0	0.5	1	0.7	1	0	0	10	3	LL
1303	2	30	1	1	0.83	1	0.9	1	1	0	4	2	SS
1304	1	26	1	1	2.83	3	2.1	3	3	2	7	3	SS
1305	2	44	2	0	0.33	1	0.4	1	0	0	0	1	SL
1306	1	29	1	0	0	1	0.8	1	0	0	3	1	SL
1307	2	48	2	0	0.33	1	0	1	0	0	1	1	SS
1308	2	39	2	1	0.83	1	0.2	1	3	2	3	1	SL
1309	2	26	1	0	0.5	1	0.2	1	1	0	1	1	SL
1310	1	22	1	0	2	3	2.2	3	1	0	4	2	SL
1311	2	20	1	1	0	1	0	1	0	0	0	1	LL
1312	1	32	2	1	1.33	2	2.3	3	4	2	2	1	LL
1313	2	40	2	1	1.17	2	1.2	2	4	2	8	3	LL
1314	1	18	1	0	0	1	0	1	2	1	0	1	LL
1315	2	30	1	1	0.5	1	1.3	2	0	0	5	2	SL
1316	2	47	2	1	1.83	2	1.2	2	1	0	3	1	SL
1317	2	43	2	0	0	1	0.8	1	2	1	1	1	SL
1318	1	36	2	1	1.33	2	1.4	2	0	0	8	3	SL
1319	2	41	2	0	0.33	1	0.4	1	2	1	4	2	SS
1320	2	47	2	1	0.67	1	0	1	1	0	2	1	LL
1321	1	33	2	0	0.5	1	1.5	2	1	0	4	2	SS
1322	2	33	2	0	0.67	1	0.8	1	0	0	5	2	SL
1323	2	24	1	0	1.17	2	0.8	1	2	1	2	1	SL
1324	2	26	1	1	3.67	3	2.8	3	0	0	2	1	SL
1325	1	33	2	1	0.83	1	0.7	1	0	0	5	2	SL
1326	2	43	2	1	0.5	1	1.1	2	2	1	2	1	SS
1327	2	30	1	0	0.83	1	0.3	1	3	2	2	1	SL
1328	2	29	1	0	0	1	0.2	1	0	0	0	1	LL
1329	2	22	1	0	0.67	1	0.7	1	4	2	1	1	LL
1330	2	31	2	1	1.17	2	1.7	2	1	0	11	3	LL
1331	2	49	2	1	3	3	2.1	3	1	0	8	3	LL
1332	2	35	2	1	1.33	2	1.9	2	3	2	6	2	LL
1333	2	49	2	0	0	1	0	1	0	0	6	2	SS
1334	2	39	2	0	0.33	1	0.2	1	2	1	2	1	SL
1335	2	26	1	0	0.33	1	0.3	1	1	0	2	1	SL
1336	2	23	1	0	2.5	3	1.8	2	1	0	1	1	SL
1337	1	41	2	0	0.33	1	0.4	1	1	0	3	1	SS
1338	2	29	1	0	2	3	1.2	2	1	0	2	1	SS
1339	1	43	2	1	1.33	2	0.4	1	0	0	9	3	SL
1340	1	33	2	1	1.67	2	2.3	3	0	0	1	1	SL
1341	2	55	2	1	1.8	2	1.2	2	1	0	10	3	LL
1342	1	44	2	1	1	2	0.2	1	1	0	6	2	SS
1343	2	48	2	1	3	3	3	3	1	0	7	3	LL
1344	2	40	2	1	0.83	1	2.1	3	4	2	5	2	LL
1345	2	48	2	1	3.33	3	1.9	2	3	2	0	1	SL
1346	1	35	2	0	0.83	1	0.6	1	2	1	2	1	SS
1347	1	43	2	0	0.33	1	0.2	1	3	2	1	1	SL
1348	2	27	1	1	1.17	2	1.5	2	3	2	1	1	SL
1349	2	39	2	1	2.33	3	2.9	3	3	2	12	3	SS
1350	2	31	2	1	0.17	1	0.1	1	2	1	5	2	SS
1351	2	25	1	0	1.67	2	0.8	1	2	1	0	1	SL
1352	2	43	2	0	0	1	0	1	0	0	0	1	SL
1353	2	48	2	0	0	1	0.1	1	1	0	0	1	SL
1354	1	34	2	1	0.83	1	0.1	1	1	0	2	1	SL

1355	1	44	2	0	1.17	2	0.3	1	0	0	2	1	SL
1356	2	43	2	1	1.5	2	1.1	2	1	0	7	3	SL
1357	2	31	2	1	2.5	3	1.7	2	4	2	3	1	SL
1358	2	35	2	0	1	2	0.4	1	2	1	0	1	LL
1359	2	46	2	0	0.17	1	0.1	1	0	0	4	2	SS
1360	2	36	2	1	1.17	2	1.1	2	1	0	3	1	SS
1361	1	34	2	1	0.83	1	1.2	2	4	2	0	1	SL
1362	2	37	2	1	1.33	2	0.7	1	0	0	13	3	LL
1363	2	34	2	1	3.5	3	2.2	3	2	1	5	2	LL
1364	1	33	2	1	1.17	2	1.8	2	3	2	3	1	SL
1365	2	35	2	1	0.17	1	0.1	1	0	0	11	3	LL
1366	2	38	2	1	1.5	2	2.5	3	1	0	8	3	LL
1367	2	38	2	1	0.33	1	0.3	1	2	1	4	2	SL
1368	2	40	2	1	2.33	3	1.4	2	1	0	10	3	LL
1369	1	30	1	1	0.17	1	0	1	1	0	6	2	SL
1370	1	26	1	1	0.67	1	1.3	2	0	0	2	1	SL
1371	2	39	2	1	0.17	1	2.6	3	1	0	10	3	SL
1372	2	46	2	1	0.33	1	0.3	1	0	0	1	1	SL
1373	2	38	2	1	0	1	0.2	1	0	0	1	1	LL
1374	2	44	2	1	1.67	2	0.3	1	0	0	0	1	SL
1375	1	43	2	0	0.33	1	0.1	1	0	0	7	3	LL
1376	2	38	2	1	0.8	1	1.4	2	2	1	5	2	SS
1377	1	48	2	0	0.17	1	0.3	1	2	1	4	2	SS
1378	1	48	2	0	0.17	1	0	1	1	0	0	1	SL
1379	2	37	2	1	2	3	1.7	2	0	0	8	3	SL
1380	1	48	2	1	2	3	2.8	3	2	1	7	3	SL
1381	2	53	2	1	0.33	1	0.8	1	2	1	5	2	LL
1382	2	60	2	0	0.33	1	0	1	1	0	8	3	SL
1383	2	40	2	0	0.33	1	0.2	1	2	1	0	1	LL
1384	2	38	2	1	1.33	2	1.7	2	1	0	4	2	LL
1385	2	26	1	0	1.5	2	1.6	2	5	2	1	1	SS
1386	2	47	2	1	0.17	1	0.5	1	2	1	7	3	LL
1387	2	44	2	0	0.17	1	0.1	1	0	0	0	1	LL
1388	2	20	1	0	2.5	3	1.2	2	1	0	6	2	SS
1389	2	26	1	1	2.4	3	2.8	3	2	1	6	2	LL
1390	2	44	2	1	0.5	1	0.3	1	0	0	1	1	SL
1391	2	40	2	1	3	3	2.2	3	3	2	5	2	SL
1392	2	42	2	0	0	1	0	1	0	0	0	1	SL
1393	2	43	2	0	0	1	0	1	0	0	11	3	SL
1394	2	19	1	0	0.33	1	0	1	3	2	0	1	SL
1395	2	42	2	1	1.33	2	2.3	3	0	0	7	3	SL
1396	2	38	2	0	0	1	0.1	1	2	1	0	1	SL
1397	2	33	2	1	1	2	0.6	1	2	1	8	3	LL
1398	1	39	2	1	0.83	1	1.3	2	2	1	10	3	SL
1399	2	42	2	1	0.33	1	0	1	1	0	0	1	SL
1400	2	40	2	1	0	1	0	1	0	0	1	1	SL
1401	2	44	2	1	0.5	1	0	1	1	0	0	1	SS
1402	1	36	2	0	0.33	1	0.2	1	0	0	0	1	SL
1403	2	45	2	0	0.33	1	0.3	1	1	0	2	1	SS
1404	2	24	1	1	0.5	1	0.3	1	0	0	4	2	LL
1405	2	30	1	1	3	3	2.7	3	0	0	8	3	SL
1406	2	60	2	0	2.4	3	1	2	0	0	2	1	LL
1407	2	43	2	1	0.8	1	0.5	1	1	0	2	1	LL
1408	1	42	2	1	3.33	3	3.4	3	3	2	9	3	SL
1409	2	45	2	0	1.5	2	1.6	2	3	2	1	1	LL
1410	1	18	1	0	0	1	0.2	1	4	2	3	1	LL
1411	2	60	2	0	0	1	0.7	1	1	0	10	3	LL
1412	2	45	2	1	2	3	3.1	3	0	0	10	3	SL
1413	2	42	2	0	0.17	1	0.1	1	0	0	10	3	SS

1414	2	20	1	1	2.67	3	1.8	2	3	2	11	3	SL
1415	2	23	1	1	1.17	2	1.7	2	0	0	3	1	SS
1416	2	26	1	0	0.17	1	0.1	1	0	0	1	1	SL
1417	2	37	2	0	0.83	1	1.6	2	1	0	2	1	LL
1418	1	33	2	0	0.17	1	1.2	2	0	0	0	1	LL
1419	2	33	2	1	0.33	1	3	3	1	0	4	2	LL
1420	2	38	2	0	0.83	1	0.5	1	2	1	2	1	SL
1421	1	25	1	0	0	1	0.5	1	2	1	2	1	LL
1422	1	30	1	0	0.17	1	0.2	1	0	0	4	2	SL
1423	1	42	2	0	0	1	0	1	0	0	0	1	SL
1424	2	32	2	0	0.17	1	0.1	1	0	0	1	1	LL
1425	2	60	2	0	0	1	0.2	1	1	0	9	3	SL
1426	1	47	2	1	1.5	2	0.3	1	0	0	0	1	SL
1427	2	33	2	1	2.33	3	0.9	1	1	0	0	1	SL
1428	1	49	2	0	0.33	1	0	1	0	0	4	2	SL
1429	2	32	2	0	0	1	0	1	1	0	1	1	SL
1430	1	49	2	0	0	1	0.6	1	2	1	2	1	SL
1431	1	43	2	0	0.17	1	0.4	1	0	0	1	1	LL
1432	1	40	2	1	0	1	0.3	1	0	0	4	2	LL
1433	1	32	2	0	0.5	1	0.8	1	0	0	3	1	LL
1434	2	44	2	1	0.5	1	0	1	0	0	12	3	LL
1435	2	44	2	0	0.33	1	0.1	1	0	0	0	1	SS
1436	2	45	2	1	1.67	2	1	2	1	0	3	1	LL
1437	1	44	2	0	0.5	1	0.1	1	0	0	0	1	LL
1438	2	60	2	1	0.33	1	1.1	2	2	1	13	3	LL
1439	1	37	2	0	0.5	1	0.9	1	1	0	2	1	LL
1440	2	37	2	0	0	1	0.1	1	1	0	0	1	SL
1441	2	45	2	0	0.33	1	0.1	1	0	0	3	1	SS
1442	1	24	1	0	0.33	1	0	1	0	0	2	1	SL
1443	1	48	2	1	1.17	2	0.4	1	1	0	0	1	SS
1444	1	32	2	0	0.33	1	0.3	1	0	0	1	1	LL
1445	1	37	2	1	0	1	0.2	1	0	0	2	1	LL
1446	2	37	2	1	1.83	2	2.6	3	0	0	4	2	SL
1447	2	42	2	1	1.5	2	2.3	3	2	1	14	3	LL
1448	2	24	1	1	1.83	2	2.8	3	3	2	1	1	SL
1449	2	36	2	1	1.33	2	2	3	2	1	2	1	SL
1450	1	34	2	1	2.33	3	2.6	3	0	0	5	2	SL
1451	1	32	2	1	0.33	1	0.4	1	1	0	2	1	SL
1452	2	35	2	0	0.33	1	0.1	1	0	0	2	1	SL
1453	2	47	2	0	0.33	1	0.4	1	0	0	5	2	SL
1454	2	48	2	0	0.17	1	0.1	1	2	1	1	1	SL
1455	1	42	2	1	0.5	1	0.1	1	0	0	4	2	SS
1456	1	19	1	0	0	1	0	1	1	0	2	1	LL
1457	2	44	2	0	0.5	1	0.6	1	3	2	3	1	SL
1458	2	30	1	0	0.83	1	0.3	1	0	0	1	1	SL
1459	1	32	2	1	1	2	2.3	3	3	2	3	1	SS
1460	2	39	2	0	0.17	1	0.1	1	0	0	0	1	LL
1461	2	19	1	0	0.67	1	0.3	1	2	1	0	1	LL
1462	1	47	2	0	1.5	2	2.3	3	1	0	3	1	SL
1463	2	30	1	1	1.67	2	2.9	3	4	2	6	2	SS
1464	2	25	1	0	0	1	0.1	1	0	0	0	1	SL
1465	2	34	2	0	0.67	1	0.7	1	0	0	3	1	SS
1466	1	24	1	0	0	1	0	1	1	0	1	1	SL
1467	1	37	2	1	1.33	2	2.9	3	0	0	3	1	SL
1468	1	30	1	0	0.5	1	0.2	1	3	2	5	2	SL
1469	2	26	1	0	0	1	1.8	2	3	2	8	3	LL
1470	2	39	2	1	0.33	1	0.6	1	0	0	1	1	SL
1471	1	26	1	0	0	1	0.3	1	0	0	2	1	SL
1472	2	32	2	0	0.17	1	0.2	1	1	0	1	1	SL

1473	2	60	2	1	3.5	3	2.7	3	2	1	11	3	SL
1474	2	47	2	1	3.5	3	4	3	1	0	13	3	SL
1475	2	45	2	1	0.33	1	3.27	3	0	0	8	3	SL
1476	2	25	1	1	1.17	2	1.2	2	2	1	6	2	SS
1477	2	28	1	1	2.5	3	2.6	3	1	0	2	1	SL
1478	2	49	2	1	3	3	3.5	3	2	1	8	3	SL
1479	2	25	1	1	0.33	1	0.6	1	2	1	0	1	SL
1480	1	44	2	0	0	1	0	1	0	0	1	1	SL
1481	1	60	2	1	3.5	3	3.7	3	5	2	10	3	SL
1482	2	40	2	0	0	1	0	1	0	0	4	2	LL
1483	2	53	2	1	2.17	3	2.6	3	1	0	14	3	LL
1484	2	42	2	1	0.33	1	0.4	1	1	0	2	1	LL
1485	2	40	2	0	0.83	1	0.7	1	1	0	4	2	SL
1486	1	42	2	0	0	1	0.1	1	0	0	3	1	SL
1487	2	22	1	1	1.5	2	2.7	3	0	0	1	1	SS
1488	2	37	2	1	2.5	3	1.9	2	0	0	3	1	SL
1489	2	27	1	1	0.5	1	0.5	1	1	0	2	1	SS
1490	1	32	2	1	0.83	1	1.3	2	5	2	7	3	SL
1491	2	26	1	1	0.67	1	0	1	0	0	6	2	SL
1492	2	33	2	0	0.67	1	0.9	1	1	0	0	1	SL
1493	2	26	1	1	0.83	1	0.7	1	1	0	4	2	SL
1494	1	32	2	0	0.17	1	0	1	1	0	4	2	SL
1495	2	31	2	0	0.67	1	0.7	1	0	0	0	1	SL
1496	2	50	2	1	2.33	3	2.7	3	3	2	0	1	SL
1497	1	40	2	1	0.67	1	0.1	1	2	1	7	3	LL
1498	1	45	2	1	0.33	1	0.1	1	0	0	2	1	SL
1499	2	30	1	0	1.33	2	0.8	1	2	1	2	1	SS
1500	2	27	1	0	0.5	1	0.7	1	1	0	3	1	LL
1501	2	31	2	1	1.83	2	3.1	3	1	0	8	3	SL
1502	2	35	2	1	0.5	1	0.6	1	2	1	0	1	SL
1503	2	39	2	1	2.83	3	1	2	1	0	6	2	SL
1504	1	32	2	0	0.17	1	0.2	1	0	0	1	1	SL
1505	1	27	1	0	0	1	0	1	1	0	3	1	LL
1506	2	37	2	1	0.33	1	1	2	0	0	5	2	SL
1507	2	28	1	1	1	2	0.1	1	1	0	1	1	SL
1508	1	28	1	1	3.83	3	3.3	3	0	0	9	3	SS
1509	2	19	1	1	3	3	3.6	3	2	1	14	3	SL
1510	2	51	2	1	0.2	1	0	1	1	0	7	3	SL
1511	2	31	2	0	0.5	1	0.1	1	3	2	2	1	SL
1512	2	43	2	0	0.67	1	0.7	1	3	2	4	2	SL
1513	1	40	2	1	1.67	2	2.6	3	1	0	3	1	SS
1514	2	31	2	0	0.5	1	0	1	0	0	0	1	SS
1515	2	46	2	1	2	3	1.6	2	1	0	5	2	SL
1516	2	28	1	1	0.67	1	1.7	2	2	1	9	3	SL
1517	2	32	2	1	2.17	3	1.4	2	0	0	4	2	SS
1518	2	27	1	0	0.17	1	0.3	1	0	0	0	1	LL
1519	2	41	2	1	0.83	1	0.9	1	1	0	0	1	SL
1520	1	33	2	0	2	3	1.3	2	1	0	1	1	SS
1521	2	35	2	1	2.67	3	3.77	3	1	0	2	1	SL
1522	2	37	2	0	1.67	2	2.5	3	0	0	6	2	SL
1523	2	23	1	1	1.5	2	2.7	3	2	1	2	1	SL
1524	2	30	1	0	0.67	1	0.6	1	1	0	4	2	SL
1525	2	46	2	0	1.5	2	2.1	3	1	0	2	1	SL
1526	2	19	1	0	1	2	1.8	2	5	2	4	2	LL
1527	2	20	1	0	0.33	1	0.7	1	0	0	1	1	SL
1528	2	39	2	1	2.5	3	2.5	3	4	2	11	3	SL
1529	2	46	2	1	0	1	0.1	1	0	0	0	1	SL
1530	2	36	2	0	0	1	0.2	1	0	0	0	1	SL
1531	2	42	2	0	0.33	1	0.6	1	0	0	12	3	LL

1532	2	30	1	0	0.17	1	0.8	1	1	0	0	1	SL
1533	2	24	1	1	0.33	1	0.8	1	0	0	8	3	SL
1534	2	25	1	0	0.17	1	0.1	1	1	0	0	1	SS
1535	2	42	2	1	3.67	3	3.2	3	4	2	13	3	SL
1536	2	23	1	1	0.5	1	0.9	1	2	1	3	1	LL
1537	2	33	2	1	0.67	1	0.3	1	0	0	2	1	LL
1538	2	20	1	1	0.5	1	0.7	1	4	2	3	1	LL
1539	2	18	1	1	2	3	1.2	2	2	1	9	3	SL
1540	1	22	1	0	0.83	1	1	2	0	0	2	1	SL
1541	2	31	2	0	0.17	1	0.2	1	1	0	0	1	SL
1542	1	20	1	1	3	3	2.2	3	5	2	10	3	SL
1543	2	34	2	1	4	3	2.4	3	0	0	1	1	SS
1544	2	37	2	1	0.17	1	0.2	1	0	0	10	3	SL
1545	2	38	2	1	0.67	1	0.8	1	1	0	3	1	SL
1546	2	37	2	1	0.33	1	0.2	1	0	0	0	1	SS
1547	2	21	1	1	2.17	3	2	3	2	1	0	1	SL
1548	2	39	2	1	0	1	0	1	1	0	2	1	LL
1549	2	36	2	1	1.17	2	0.4	1	1	0	0	1	LL
1550	2	25	1	1	1.17	2	1.4	2	1	0	0	1	SL
1551	2	45	2	1	2.83	3	2.8	3	2	1	0	1	LL
1552	2	27	1	0	2	3	3	3	2	1	1	1	LL
1553	2	45	2	1	2.33	3	0.7	1	2	1	0	1	SL
1554	1	30	1	1	3	3	0.7	1	1	0	0	1	SS
1555	1	33	2	0	0.17	1	0.7	1	2	1	1	1	SS
1556	2	26	1	1	2.33	3	0.7	1	2	1	3	1	LL
1557	2	19	1	1	0.5	1	0.8	1	0	0	0	1	SL
1558	2	42	2	1	3.5	3	3.5	3	2	1	12	3	LL
1559	2	36	2	1	1.5	2	3.4	3	1	0	2	1	LL
1560	2	27	1	1	1.33	2	2.1	3	4	2	0	1	LL
1561	1	31	2	1	0.33	1	0.8	1	3	2	2	1	LL
1562	1	46	2	1	3	3	3	3	3	2	2	1	LL
1563	2	43	2	1	0.83	1	2.7	3	1	0	5	2	SS
1564	1	45	2	1	0.17	1	0.7	1	0	0	8	3	LL
1565	2	21	1	0	0.17	1	0.7	1	1	0	3	1	LL
1566	2	41	2	1	1.67	2	2.1	3	1	0	8	3	SS
1567	2	19	1	1	2	3	2.2	3	0	0	4	2	SS
1568	2	29	1	1	0.5	1	0.7	1	3	2	2	1	LL
1569	1	19	1	0	1.33	2	2.6	3	1	0	1	1	LL
1570	2	32	2	1	1.5	2	2.1	3	1	0	10	3	SS
1571	2	22	1	0	0.5	1	0.3	1	4	2	5	2	LL
1572	2	26	1	0	0	1	0.2	1	1	0	0	1	SL
1573	2	28	1	1	2.67	3	2	3	4	2	4	2	SS
1574	1	49	2	1	0.67	1	0.4	1	1	0	0	1	LL
1575	2	47	2	0	0.67	1	1.2	2	0	0	1	1	SL
1576	2	27	1	1	3.33	3	3.4	3	6	2	1	1	SL
1577	2	39	2	1	1.67	2	1.7	2	0	0	11	3	SL
1578	2	38	2	1	1.33	2	1.8	2	0	0	2	1	LL
1579	2	45	2	1	3.17	3	2.8	3	2	1	7	3	LL
1580	2	26	1	1	3	3	2.9	3	0	0	12	3	LL
1581	1	51	2	1	0.83	1	1.2	2	2	1	4	2	SS
1582	2	37	2	0	0.17	1	0.2	1	0	0	2	1	SS
1583	2	42	2	1	0.5	1	2.8	3	0	0	13	3	LL
1584	2	23	1	0	2.33	3	3.4	3	0	0	3	1	SL
1585	2	19	1	0	0.17	1	0.4	1	1	0	1	1	SL
1586	2	37	2	1	0	1	0.2	1	1	0	2	1	LL
1587	2	33	2	0	0.83	1	0.3	1	0	0	2	1	LL
1588	2	42	2	0	0.67	1	0.2	1	0	0	0	1	SL
1589	2	38	2	0	0	1	0	1	2	1	0	1	LL
1590	2	48	2	1	3.33	3	3.4	3	1	0	9	3	SS

1591	2	21	1	1	1.17	2	1.2	2	1	0	3	1	LL
1592	2	21	1	1	1.67	2	3.5	3	6	2	3	1	LL
1593	1	24	1	1	3.33	3	3.1	3	4	2	16	3	SS
1594	2	24	1	0	2.33	3	2.4	3	3	2	8	3	LL
1595	2	18	1	0	0.83	1	1.1	2	1	0	1	1	SS
1596	2	42	2	1	0.67	1	0.7	1	1	0	9	3	SL
1597	2	28	1	1	1.33	2	2.1	3	1	0	2	1	SL
1598	2	34	2	1	0.67	1	0.7	1	3	2	2	1	LL
1599	2	41	2	1	2.83	3	1.4	2	0	0	2	1	SS
1600	2	34	2	1	2.17	3	2.2	3	6	2	13	3	SL
1601	2	42	2	0	0.67	1	1.2	2	2	1	6	2	SS
1602	2	36	2	1	0.67	1	0.4	1	1	0	0	1	SL
1603	2	27	1	1	0.17	1	0.1	1	1	0	4	2	SL
1604	2	30	1	1	0.17	1	1.2	2	0	0	2	1	SL
1605	2	44	2	1	2.67	3	3	3	1	0	7	3	SL
1606	1	22	1	1	0.17	1	0.9	1	0	0	8	3	SL
1607	2	21	1	0	2.17	3	2.2	3	2	1	5	2	SL
1608	2	43	2	1	2.17	3	3.7	3	2	1	14	3	LL
1609	2	38	2	1	0.83	1	1.9	2	1	0	2	1	LL
1610	2	45	2	1	0.4	1	1	2	0	0	6	2	LL
1611	2	46	2	1	0.83	1	0.6	1	4	2	8	3	SS
1612	2	50	2	1	0.17	1	0.9	1	0	0	4	2	SL
1613	2	18	1	1	1	2	1.4	2	2	1	4	2	SL
1614	2	19	1	1	0.83	1	0.9	1	2	1	1	1	SL
1615	1	20	1	1	1.83	2	2.2	3	0	0	12	3	SL
1616	2	27	1	1	0.33	1	2.6	3	1	0	8	3	LL
1617	1	47	2	1	0.83	1	0.5	1	2	1	5	2	LL
1618	2	38	2	0	0.33	1	0.1	1	1	0	10	3	SL
1619	2	30	1	1	2	3	1.2	2	3	2	1	1	SL
1620	2	20	1	1	2.83	3	2.8	3	2	1	5	2	SL
1621	2	19	1	0	0	1	0.6	1	3	2	3	1	SS
1622	2	19	1	1	1	2	1.7	2	3	2	5	2	LL
1623	2	48	2	1	0.33	1	0.8	1	1	0	4	2	LL
1624	2	26	1	1	3.5	3	1.6	2	3	2	0	1	SL
1625	2	21	1	1	1	2	1.1	2	2	1	0	1	SL
1626	2	19	1	0	0	1	1.1	2	2	1	1	1	SL
1627	1	23	1	1	2.33	3	2.4	3	3	2	6	2	SL
1628	2	42	2	1	0.5	1	0.1	1	0	0	0	1	SL
1629	2	24	1	0	0.67	1	0.7	1	3	2	0	1	SS
1630	1	20	1	1	0	1	0.8	1	2	1	5	2	SL
1631	2	33	2	1	2.5	3	2.3	3	2	1	6	2	SL
1632	2	42	2	1	0.67	1	0.7	1	0	0	6	2	SS
1633	1	24	1	0	0.5	1	1.5	2	1	0	4	2	LL
1634	2	20	1	1	1.33	2	2	3	3	2	5	2	LL
1635	2	32	2	1	1.5	2	0.5	1	0	0	6	2	LL
1636	2	42	2	1	1	2	1.3	2	0	0	3	1	SL
1637	1	27	1	1	2	3	2.2	3	0	0	7	3	LL
1638	2	26	1	1	1.5	2	1.1	2	0	0	5	2	SL
1639	2	30	1	1	1.33	2	1.6	2	4	2	0	1	LL
1640	2	24	1	1	0.67	1	0.5	1	3	2	4	2	LL
1641	2	41	2	0	1	2	0.1	1	1	0	16	3	SL
1642	2	36	2	1	1.83	2	1.9	2	2	1	4	2	SL
1643	2	53	2	1	2	3	1.16	2	1	0	13	3	SL
1644	2	50	2	1	3.33	3	1.5	2	0	0	0	1	LL
1645	2	37	2	1	3.33	3	3.6	3	3	2	9	3	LL
1646	2	33	2	1	3.83	3	3.4	3	2	1	11	3	SL
1647	2	44	2	1	1.33	2	1.9	2	0	0	4	2	SL
1648	2	27	1	1	2	3	2.2	3	3	2	6	2	LL
1649	1	22	1	1	2.67	3	1.6	2	3	2	5	2	SL

1650	2	40	2	1	0.5	1	1.4	2	2	1	6	2	SL
1651	1	30	1	1	0.33	1	0.7	1	0	0	4	2	LL
1652	2	20	1	0	1.17	2	0.7	1	0	0	0	1	SL
1653	2	44	2	1	1.67	2	2.5	3	2	1	2	1	SL
1654	2	22	1	1	3.67	3	3.4	3	4	2	4	2	LL
1655	2	37	2	1	3	3	2.2	3	2	1	3	1	LL
1656	1	22	1	0	0	1	0.6	1	2	1	0	1	SL
1657	2	36	2	1	1.33	2	2.1	3	1	0	2	1	SL
1658	2	33	2	0	1	2	1.9	2	0	0	9	3	LL
1659	2	28	1	0	1.67	2	0.2	1	1	0	3	1	SS
1660	2	24	1	0	0	1	0.1	1	0	0	0	1	LL
1661	2	20	1	0	2.83	3	2.3	3	2	1	1	1	SL
1662	2	34	2	1	1.17	2	1.7	2	2	1	15	3	SS
1663	2	29	1	1	1.67	2	2.1	3	3	2	1	1	LL
1664	2	24	1	1	2.17	3	2.5	3	4	2	7	3	SL
1665	2	20	1	1	0.17	1	0.3	1	2	1	4	2	SS
1666	1	21	1	1	1.17	2	2.4	3	0	0	12	3	SL
1667	1	38	2	1	2.33	3	3.5	3	6	2	7	3	SS
1668	2	23	1	0	0.33	1	0.4	1	4	2	0	1	LL
1669	2	18	1	1	0.83	1	1.2	2	1	0	8	3	LL
1670	2	20	1	1	0	1	0.5	1	3	2	1	1	SL
1671	2	25	1	0	0	1	0	1	0	0	14	3	SL
1672	2	35	2	1	0.33	1	0.22	1	1	0	3	1	SL
1673	2	32	2	1	3.67	3	3.6	3	1	0	12	3	LL
1674	2	34	2	1	1.5	2	1.7	2	2	1	6	2	SS
1675	1	35	2	1	1	2	1.5	2	3	2	4	2	SS
1676	2	47	2	1	1	2	1.6	2	4	2	7	3	SS
1677	2	31	2	1	0.67	1	0.4	1	3	2	4	2	SL
1678	2	23	1	1	2.5	3	2.3	3	0	0	2	1	SL
1679	2	43	2	1	3.33	3	3.5	3	1	0	4	2	SL
1680	2	23	1	1	2	3	1.6	2	1	0	4	2	SS
1681	2	43	2	1	4	3	3	3	1	0	4	2	SL
1682	2	19	1	1	0.67	1	0.7	1	1	0	2	1	SL
1683	2	20	1	1	1.33	2	1.7	2	3	2	3	1	SL
1684	2	23	1	0	1.17	2	1.3	2	1	0	8	3	LL
1685	2	20	1	1	0.5	1	1.8	2	2	1	5	2	LL
1686	2	21	1	1	1.17	2	3	3	1	0	1	1	LL
1687	2	48	2	0	0.83	1	1.4	2	1	0	2	1	SL
1688	2	23	1	0	0.33	1	0.2	1	1	0	0	1	SL
1689	2	19	1	1	1.83	2	2	3	0	0	2	1	SS
1690	2	31	2	0	0	1	0	1	1	0	2	1	SL
1691	2	21	1	0	1	2	0.4	1	2	1	3	1	SL
1692	2	44	2	1	0.5	1	1.7	2	3	2	7	3	SL
1693	2	22	1	0	0.17	1	1.4	2	1	0	4	2	LL
1694	2	24	1	0	0.83	1	0.8	1	3	2	0	1	SL
1695	1	18	1	0	1.5	2	1.5	2	1	0	2	1	SL
1696	2	19	1	0	1.17	2	1.2	2	4	2	9	3	SS
1697	2	36	2	1	0.17	1	0.5	1	7	2	0	1	LL
1698	2	19	1	1	3.33	3	3.6	3	1	0	0	1	SL
1699	2	25	1	1	0.67	1	0.1	1	2	1	1	1	SL
1700	2	26	1	1	0.17	1	1	2	3	2	1	1	SL
1701	2	47	2	1	0.83	1	2.7	3	1	0	11	3	SS
1702	1	22	1	1	1.17	2	1.5	2	4	2	4	2	LL
1703	2	18	1	0	0.83	1	0.7	1	0	0	0	1	SS
1704	2	20	1	0	0.33	1	0.5	1	0	0	1	1	SS
1705	2	19	1	1	3.17	3	3.6	3	0	0	3	1	SL
1706	2	36	2	1	0.67	1	0.4	1	1	0	3	1	SL
1707	2	28	1	1	3.17	3	2.9	3	1	0	13	3	SS
1708	2	26	1	0	1	2	2.4	3	3	2	9	3	LL

1709	2	24	1	0	0.17	1	0.3	1	2	1	1	1	SS
1710	2	26	1	1	0.5	1	0.6	1	0	0	0	1	LL
1711	2	28	1	1	0.33	1	0	1	1	0	3	1	LL
1712	2	46	2	1	3.33	3	2.1	3	3	2	6	2	SL
1713	2	21	1	1	2.83	3	0.9	1	0	0	3	1	SL
1714	2	22	1	1	0.5	1	0.5	1	2	1	2	1	SL
1715	2	19	1	0	1.5	2	1.9	2	3	2	7	3	SL
1716	1	19	1	1	0	1	0.6	1	0	0	0	1	SL
1717	2	19	1	1	1.33	2	2.3	3	3	2	3	1	SL
1718	2	23	1	1	2.33	3	1.1	2	1	0	4	2	SL
1719	2	21	1	1	0.5	1	0.7	1	2	1	1	1	SL
1720	2	22	1	0	1.83	2	0.6	1	4	2	2	1	LL
1721	2	39	2	1	1	2	0.9	1	1	0	3	1	SL
1722	1	24	1	1	1.17	2	1.1	2	0	0	10	3	LL
1723	2	22	1	1	0.83	1	1.5	2	1	0	8	3	SS
1724	2	42	2	1	1	2	0.3	1	3	2	8	3	LL
1725	2	34	2	0	1.33	2	0.5	1	3	2	1	1	SL
1726	1	27	1	0	0	1	0.2	1	1	0	0	1	SL
1727	2	18	1	0	0.67	1	0.5	1	2	1	2	1	SS
1728	2	23	1	0	0.33	1	0.3	1	2	1	1	1	SS
1729	2	22	1	1	3.33	3	3.1	3	2	1	16	3	LL
1730	2	26	1	1	1.67	2	1.9	2	2	1	8	3	LL
1731	2	33	2	0	0.5	1	0.4	1	0	0	5	2	LL
1732	2	51	2	1	0.67	1	0.1	1	0	0	3	1	SS
1733	2	19	1	0	0.33	1	1	2	2	1	1	1	SS
1734	2	24	1	1	2	3	1.7	2	5	2	10	3	SL
1735	2	34	2	1	2.33	3	2.5	3	0	0	3	1	SL
1736	2	21	1	1	1.67	2	0.5	1	2	1	2	1	SS
1737	2	21	1	1	3.67	3	3.4	3	1	0	9	3	SL
1738	2	27	1	1	1.5	2	1	2	4	2	0	1	SS
1739	2	26	1	1	0.17	1	0.1	1	1	0	3	1	SL
1740	2	28	1	0	2.83	3	1.5	2	1	0	0	1	SL
1741	2	21	1	0	0.33	1	0.2	1	4	2	0	1	SL
1742	2	21	1	0	2.67	3	2.3	3	0	0	2	1	SL
1743	2	30	1	1	1.83	2	2.4	3	2	1	10	3	SL
1744	2	19	1	1	2.33	3	1.8	2	0	0	4	2	SL
1745	2	25	1	0	0.5	1	0.8	1	1	0	5	2	LL
1746	2	28	1	1	3.67	3	3.4	3	0	0	0	1	LL
1747	2	27	1	1	2.67	3	3.8	3	2	1	3	1	SS
1748	2	22	1	1	0.17	1	0.1	1	3	2	0	1	SL
1749	2	27	1	1	3.17	3	3.8	3	5	2	#NULL!	#NULL!	LL
1750	2	20	1	0	0	1	0.4	1	2	1	4	2	LL
1751	2	19	1	0	0.5	1	0.4	1	1	0	0	1	SS
1752	2	27	1	0	0.5	1	0.4	1	3	2	0	1	LL
1753	2	26	1	1	1.17	2	0.4	1	1	0	0	1	SL
1754	2	25	1	1	1.5	2	2.8	3	1	0	9	3	LL
1755	2	37	2	0	0	1	0	1	2	1	11	3	SS
1756	2	25	1	0	0.5	1	0.2	1	1	0	1	1	SL
1757	1	37	2	0	0.33	1	0.7	1	1	0	3	1	SL
1758	2	26	1	1	3.33	3	3.6	3	0	0	8	3	SL
1759	2	45	2	0	0.33	1	0	1	1	0	5	2	SL
1760	1	41	2	0	0.33	1	0.2	1	0	0	0	1	SL
1761	1	25	1	0	1.33	2	1	2	0	0	4	2	SS
1762	2	20	1	0	1.17	2	0.8	1	2	1	2	1	SL
1763	2	33	2	1	3.17	3	3.7	3	3	2	4	2	SL
1764	2	19	1	1	0.33	1	0.3	1	7	2	3	1	SL
1765	2	26	1	1	1.67	2	2.2	3	4	2	4	2	LL
1766	2	34	2	1	2	3	1.8	2	2	1	2	1	SL
1767	2	19	1	1	4	3	4	3	7	2	16	3	SL

1768	2	36	2	1	2.33	3	2.1	3	4	2	13	3	SL
1769	2	45	2	1	2.67	3	2.4	3	2	1	5	2	SL
1770	2	28	1	0	0.67	1	0.8	1	4	2	2	1	LL
1771	2	40	2	1	2	3	1.9	2	2	1	2	1	SS
1772	2	22	1	0	0.5	1	0	1	3	2	2	1	LL
1773	2	28	1	1	1.17	2	2	3	0	0	2	1	SS
1774	2	19	1	1	2.17	3	1.3	2	1	0	1	1	SL
1775	2	29	1	1	3.5	3	3.8	3	0	0	2	1	SL
1776	2	23	1	1	1.83	2	1.9	2	0	0	4	2	LL
1777	2	39	2	1	0.5	1	0.3	1	1	0	2	1	SL
1778	2	20	1	1	0.83	1	0.7	1	4	2	0	1	SS
1779	2	21	1	1	4	3	3.3	3	0	0	2	1	SL
1780	2	30	1	1	1.33	2	1.7	2	3	2	16	3	SS
1781	1	43	2	1	0.67	1	0.4	1	0	0	10	3	SS
1782	2	47	2	1	3	3	2.5	3	4	2	2	1	LL
1783	2	28	1	1	0	1	1.5	2	4	2	1	1	LL
1784	2	21	1	1	0.67	1	1.4	2	3	2	2	1	SL
1785	2	36	2	1	0.5	1	0.2	1	3	2	0	1	SL
1786	2	20	1	1	1.67	2	3.5	3	1	0	3	1	LL
1787	2	29	1	0	0	1	0.4	1	1	0	11	3	SL
1788	2	21	1	0	0.33	1	0.3	1	0	0	1	1	SS
1789	2	23	1	1	2.33	3	2.8	3	4	2	1	1	LL
1790	1	48	2	0	0.5	1	0.3	1	1	0	6	2	SL
1791	2	35	2	1	0.5	1	0	1	0	0	0	1	SL
1792	1	31	2	1	0.5	1	1.3	2	1	0	5	2	SL
1793	2	19	1	1	0.83	1	1.6	2	4	2	4	2	LL
1794	2	29	1	1	0.33	1	0.1	1	1	0	2	1	SS
1795	2	45	2	1	2.17	3	1.1	2	0	0	9	3	SL
1796	2	38	2	0	0.33	1	0.3	1	0	0	5	2	SL
1797	2	32	2	1	2.83	3	2.4	3	2	1	2	1	SS
1798	2	36	2	1	1.67	2	2.2	3	2	1	4	2	LL
1799	2	18	1	0	2.33	3	2.1	3	1	0	0	1	LL
1800	2	49	2	0	2.5	3	2.1	3	1	0	6	2	SS
1801	2	38	2	1	0.5	1	1.2	2	1	0	4	2	LL
1802	2	29	1	0	0.17	1	0.5	1	0	0	1	1	SL
1803	2	19	1	0	0.17	1	0.2	1	1	0	0	1	LL
1804	2	35	2	0	1.17	2	0.4	1	1	0	3	1	SS
1805	2	29	1	1	1.83	2	2.6	3	5	2	5	2	SL
1806	2	25	1	1	1.5	2	2.2	3	0	0	5	2	SS
1807	1	39	2	1	0.83	1	0.9	1	2	1	2	1	SL
1808	2	32	2	1	0.17	1	0.7	1	0	0	3	1	LL
1809	2	31	2	1	1	2	0.4	1	0	0	4	2	SL
1810	2	26	1	0	0.17	1	0.2	1	0	0	2	1	SL
1811	2	27	1	1	0.83	1	2	3	2	1	10	3	LL
1812	2	21	1	1	0.83	1	1.1	2	1	0	11	3	SL
1813	2	23	1	0	0.67	1	0.6	1	2	1	0	1	LL
1814	2	36	2	1	0.5	1	0.2	1	0	0	1	1	SS
1815	2	20	1	1	0.17	1	0.9	1	1	0	3	1	SS
1816	2	24	1	0	0.8	1	0.8	1	0	0	0	1	SS
1817	2	21	1	0	1.5	2	0.12	1	3	2	1	1	LL
1818	2	33	2	1	3.83	3	3.8	3	1	0	1	1	SL
1819	2	18	1	1	3	3	2.9	3	4	2	4	2	SL
1820	2	39	2	1	1	2	1.5	2	1	0	3	1	SL
1821	2	23	1	1	0.5	1	0	1	1	0	1	1	SL
1822	2	23	1	1	1.5	2	1.03	2	2	1	4	2	SS
1823	2	48	2	0	0.67	1	0.3	1	2	1	7	3	SL
1824	2	33	2	1	3.83	3	2.9	3	0	0	0	1	LL
1825	2	21	1	0	2.17	3	1.2	2	3	2	3	1	SL
1826	2	32	2	0	1.33	2	0.6	1	0	0	2	1	LL

1827	2	20	1	0	0	1	0	1	0	0	4	2	LL
1828	2	34	2	0	0	1	0	1	0	0	3	1	SS
1829	2	30	1	1	1	2	0.8	1	0	0	2	1	SL
1830	1	41	2	1	0.33	1	0.1	1	0	0	2	1	LL
1831	2	49	2	0	0.83	1	1.1	2	2	1	7	3	SL
1832	2	32	2	1	0.83	1	0.6	1	3	2	2	1	SL
1833	2	38	2	0	0	1	0	1	1	0	0	1	SL
1834	2	27	1	1	2.17	3	2.5	3	0	0	8	3	SL
1835	2	21	1	0	2	3	2.5	3	3	2	1	1	SL
1836	2	42	2	1	2.17	3	1.1	2	4	2	9	3	SL
1837	2	19	1	0	0	1	0	1	1	0	1	1	SL
1838	1	22	1	0	0.33	1	0.4	1	1	0	2	1	SL
1839	2	46	2	1	2.83	3	3.7	3	1	0	7	3	LL
1840	2	23	1	0	1	2	0.2	1	2	1	0	1	LL
1841	2	29	1	1	0.5	1	1.2	2	2	1	4	2	SS
1842	2	44	2	1	3.5	3	3.6	3	5	2	#NULL!	#NULL!	SS
1843	2	32	2	0	0.33	1	0	1	0	0	0	1	SS
1844	1	41	2	0	0.17	1	1.3	2	0	0	5	2	SL
1845	1	38	2	1	0.67	1	1.1	2	0	0	2	1	SL
1846	2	34	2	1	1.17	2	1.4	2	1	0	6	2	SL
1847	2	40	2	1	3.17	3	3	3	1	0	6	2	SS
1848	2	42	2	0	0.17	1	0	1	1	0	0	1	SL
1849	2	19	1	0	1.5	2	2.6	3	0	0	1	1	SL
1850	2	33	2	1	1.67	2	0.5	1	0	0	1	1	LL
1851	2	45	2	0	2.83	3	3.4	3	2	1	15	3	SL
1852	2	22	1	1	0.83	1	1.9	2	1	0	0	1	LL
1853	2	20	1	1	2	3	3.48	3	3	2	5	2	SL
1854	2	28	1	1	2.83	3	2.1	3	3	2	14	3	LL
1855	2	35	2	1	2.83	3	1.3	2	0	0	6	2	SL
1856	2	22	1	0	0	1	0.1	1	2	1	0	1	LL
1857	2	22	1	1	0.5	1	0.7	1	0	0	12	3	SL
1858	2	29	1	1	3.33	3	2.8	3	1	0	15	3	LL
1859	2	22	1	0	0.17	1	1.2	2	1	0	1	1	SL
1860	2	18	1	0	0.17	1	0.3	1	0	0	2	1	SL
1861	2	37	2	1	1.33	2	0.6	1	7	2	0	1	SS
1862	2	38	2	0	0	1	0.4	1	0	0	0	1	SL
1863	2	41	2	1	0.17	1	0.1	1	0	0	0	1	SL
1864	2	25	1	0	1.5	2	1.5	2	1	0	4	2	SL
1865	2	20	1	1	1.67	2	3.1	3	8	2	15	3	SL
1866	2	19	1	0	2.5	3	2.2	3	1	0	0	1	SS
1867	2	35	2	1	2.17	3	1.7	2	3	2	2	1	SL
1868	2	41	2	0	0.83	1	0.1	1	0	0	1	1	SL
1869	2	33	2	1	0.5	1	0.1	1	1	0	13	3	SS
1870	2	25	1	1	0.83	1	1.3	2	1	0	9	3	SL
1871	2	21	1	0	0.33	1	0.5	1	3	2	0	1	SL
1872	2	40	2	1	2.67	3	3.6	3	2	1	8	3	LL
1873	2	18	1	1	1.33	2	1.1	2	2	1	4	2	SS
1874	2	41	2	1	2	3	2.3	3	2	1	1	1	SS
1875	2	36	2	0	0.17	1	0.6	1	0	0	0	1	LL
1876	2	21	1	0	0	1	0	1	1	0	0	1	SL
1877	1	50	2	0	1.5	2	1.4	2	2	1	1	1	SS
1878	2	24	1	1	1.5	2	0.2	1	4	2	7	3	LL
1879	2	29	1	1	3.67	3	2.7	3	2	1	2	1	SL
1880	2	19	1	0	2.5	3	2.6	3	2	1	5	2	SL
1881	2	43	2	1	0	1	0.3	1	1	0	2	1	SL
1882	1	49	2	0	0	1	0.2	1	0	0	5	2	SS
1883	1	21	1	0	0.33	1	0.2	1	1	0	2	1	LL
1884	1	20	1	0	0.33	1	0.9	1	0	0	1	1	LL
1885	2	36	2	1	0.5	1	0.5	1	0	0	8	3	SL

1886	2	20	1	1	0.17	1	0	1	2	1	5	2	SS
1887	1	24	1	0	0.67	1	0.7	1	0	0	1	1	SS
1888	2	21	1	1	2.67	3	2.9	3	4	2	6	2	SL
1889	1	23	1	1	2.5	3	1.2	2	1	0	1	1	SL
1890	2	18	1	1	1	2	3.1	3	0	0	7	3	LL
1891	2	32	2	1	0.67	1	0.6	1	6	2	8	3	SL
1892	2	42	2	1	1	2	1.36	2	0	0	3	1	SL
1893	2	39	2	1	0.5	1	1.7	2	0	0	12	3	LL
1894	1	47	2	1	1.83	2	2.4	3	3	2	9	3	SL
1895	2	35	2	0	0.67	1	0.4	1	0	0	0	1	SL
1896	2	48	2	1	2.67	3	2.1	3	1	0	3	1	SS
1897	2	42	2	1	0.83	1	1.6	2	0	0	0	1	LL
1898	2	35	2	1	0	1	0.1	1	1	0	2	1	SL
1899	2	30	1	1	0.17	1	0.5	1	1	0	3	1	SL
1900	2	20	1	1	2	3	2.1	3	3	2	8	3	SL
1901	2	18	1	0	1.67	2	2.4	3	5	2	2	1	SL
1902	2	18	1	1	2.83	3	2.4	3	3	2	2	1	LL
1903	2	27	1	1	0.17	1	0.2	1	0	0	3	1	SL
1904	2	42	2	1	3.5	3	3	3	3	2	11	3	LL
1905	2	46	2	1	1.17	2	2.2	3	3	2	0	1	LL
1906	2	49	2	1	0.67	1	0.4	1	0	0	3	1	SL
1907	1	35	2	0	0	1	0.9	1	2	1	5	2	SL
1908	2	34	2	1	0.5	1	0.5	1	3	2	2	1	SL
1909	1	47	2	1	2	3	1.7	2	1	0	11	3	LL
1910	2	57	2	1	3	3	3.1	3	0	0	14	3	SL
1911	2	38	2	1	2.8	3	2.4	3	2	1	5	2	SL
1912	2	37	2	0	1.5	2	2.4	3	1	0	7	3	SL
1913	2	29	1	1	1.17	2	2.5	3	2	1	10	3	SL
1914	2	33	2	1	1.67	2	2.5	3	2	1	1	1	LL
1915	2	45	2	1	1.67	2	2.2	3	1	0	1	1	SL
1916	2	32	2	0	0.5	1	0.7	1	0	0	9	3	SS
1917	2	22	1	0	3.67	3	0.5	1	2	1	0	1	SL
1918	2	40	2	1	0.33	1	0.7	1	2	1	6	2	LL
1919	2	43	2	1	2.5	3	1.8	2	1	0	10	3	SL
1920	2	32	2	1	3.17	3	1.8	2	4	2	6	2	LL
1921	2	19	1	0	0.5	1	0.5	1	2	1	0	1	SL
1922	2	25	1	1	1	2	0.4	1	1	0	1	1	SL
1923	2	29	1	1	0.33	1	2.5	3	2	1	2	1	SL
1924	2	25	1	1	1.67	2	1.5	2	0	0	0	1	SL
1925	1	18	1	0	0.5	1	0.7	1	0	0	0	1	LL
1926	2	33	2	0	0.17	1	0	1	0	0	1	1	LL
1927	2	19	1	1	1	2	0.7	1	3	2	4	2	LL
1928	2	32	2	1	3	3	1.9	2	0	0	10	3	LL
1929	1	48	2	1	0.33	1	2.1	3	4	2	2	1	SL
1930	2	42	2	1	2.67	3	2.2	3	0	0	14	3	SS
1931	2	39	2	1	1.17	2	0.2	1	0	0	2	1	SL
1932	2	28	1	1	1.83	2	0.9	1	1	0	3	1	LL
1933	2	25	1	1	1	2	0.7	1	0	0	8	3	LL
1934	2	21	1	0	0.67	1	0.8	1	0	0	3	1	SL
1935	2	18	1	1	2.67	3	1.8	2	1	0	3	1	SL
1936	2	26	1	1	0.83	1	0.2	1	1	0	1	1	SL
1937	1	25	1	0	0.5	1	0.8	1	2	1	2	1	SL
1938	2	29	1	0	1.83	2	3.6	3	2	1	3	1	SL
1939	2	41	2	1	0.33	1	0	1	2	1	3	1	LL
1940	2	23	1	1	1.33	2	2	3	0	0	0	1	SL
1941	1	19	1	1	0.5	1	1.1	2	0	0	6	2	SL
1942	1	45	2	0	0	1	1.6	2	2	1	7	3	SL
1943	2	20	1	0	1.67	2	2.3	3	1	0	1	1	SS
1944	2	26	1	1	0.5	1	0.5	1	0	0	0	1	SL

1945	2	24	1	1	0.67	1	0.5	1	1	0	0	1	SL
1946	2	31	2	1	3	3	2.2	3	2	1	7	3	SS
1947	2	20	1	1	2.5	3	2.5	3	3	2	7	3	LL
1948	1	47	2	0	2.17	3	2.8	3	1	0	6	2	LL
1949	2	48	2	1	1.33	2	1.6	2	2	1	3	1	LL
1950	2	30	1	1	3.83	3	3.9	3	2	1	2	1	SL
1951	2	49	2	1	1.5	2	1.5	2	3	2	1	1	LL
1952	1	35	2	0	0.67	1	0.2	1	0	0	0	1	SL
1953	2	23	1	1	2.17	3	2.8	3	1	0	9	3	SS
1954	2	19	1	1	0.83	1	1.9	2	5	2	3	1	LL
1955	2	25	1	1	0.67	1	2.4	3	0	0	13	3	LL
1956	2	50	2	1	0.4	1	0.5	1	0	0	0	1	LL
1957	2	26	1	0	0	1	0.5	1	1	0	9	3	LL
1958	1	36	2	1	1.5	2	2.4	3	0	0	8	3	SL
1959	2	25	1	1	1.83	2	1.3	2	2	1	1	1	LL
1960	2	27	1	0	0.33	1	0.6	1	2	1	1	1	LL
1961	1	36	2	0	0.5	1	0.2	1	1	0	2	1	SL
1962	2	29	1	1	2.4	3	1.7	2	2	1	1	1	SL
1963	2	22	1	0	2	3	0.8	1	4	2	2	1	SL
1964	2	30	1	0	0	1	0	1	1	0	2	1	LL
1965	2	39	2	0	0	1	0.2	1	0	0	2	1	LL
1966	2	22	1	1	2.33	3	2.8	3	2	1	13	3	SS
1967	2	34	2	1	1.17	2	1.7	2	3	2	9	3	LL
1968	2	46	2	1	0.67	1	0	1	0	0	8	3	SS
1969	2	35	2	0	0.17	1	0	1	1	0	8	3	SL
1970	2	28	1	0	0.83	1	0.8	1	0	0	3	1	SL
1971	2	45	2	1	0.33	1	0.7	1	0	0	2	1	LL
1972	2	31	2	1	0.67	1	0.2	1	1	0	5	2	SL
1973	2	29	1	1	1.17	2	1.1	2	0	0	10	3	LL
1974	2	21	1	0	1.33	2	1	2	1	0	4	2	LL
1975	2	37	2	1	0.67	1	0.9	1	1	0	9	3	SL
1976	1	30	1	0	0.17	1	0	1	0	0	1	1	SL
1977	2	28	1	1	0.33	1	2.4	3	5	2	7	3	SL
1978	2	25	1	1	2.83	3	3	3	0	0	2	1	LL
1979	2	19	1	1	1	2	2.1	3	5	2	2	1	SL
1980	2	20	1	1	0.17	1	0	1	1	0	0	1	SL
1981	2	35	2	1	3	3	2.1	3	0	0	0	1	SL
1982	2	29	1	1	0.5	1	0.7	1	1	0	0	1	SS
1983	2	21	1	0	0.17	1	0.3	1	4	2	0	1	SS
1984	2	27	1	1	2.83	3	2.3	3	1	0	3	1	SS
1985	2	25	1	1	0.67	1	1.2	2	0	0	3	1	SL
1986	2	37	2	1	0	1	0.6	1	2	1	8	3	SS
1987	1	20	1	1	2.33	3	2.1	3	0	0	1	1	LL
1988	2	20	1	1	0.67	1	1.4	2	2	1	3	1	SL
1989	2	48	2	0	2	3	1.6	2	1	0	2	1	LL
1990	2	25	1	1	0.67	1	1	2	0	0	7	3	LL
1991	1	33	2	1	1	2	2.3	3	1	0	3	1	SL
1992	1	43	2	1	0.67	1	0.3	1	1	0	3	1	SL
1993	2	27	1	0	1.5	2	1.5	2	0	0	4	2	SS
1994	2	32	2	1	2.17	3	2.2	3	3	2	9	3	SL
1995	2	20	1	1	1.33	2	2.8	3	2	1	7	3	LL
1996	2	45	2	1	0.83	1	1	2	1	0	6	2	SL
1997	2	25	1	1	0.83	1	1.3	2	3	2	5	2	SL
1998	2	47	2	1	0.67	1	0.7	1	2	1	5	2	SL
1999	2	20	1	0	1.5	2	2.4	3	3	2	0	1	LL
2000	2	31	2	1	1.33	2	1.5	2	1	0	12	3	SS
2001	2	32	2	1	0.5	1	0.8	1	0	0	3	1	SS
2002	2	23	1	0	1.17	2	0.7	1	2	1	2	1	LL
2003	1	27	1	1	0.17	1	0.1	1	0	0	7	3	SS

2004	2	36	2	1	3.67	3	1.8	2	2	1	7	3	SS
2005	2	38	2	1	0.5	1	0.6	1	2	1	4	2	SS
2006	2	45	2	1	2	3	1.9	2	5	2	2	1	SL
2007	2	22	1	0	0.67	1	1.3	2	5	2	8	3	LL
2008	2	28	1	1	2.5	3	3.5	3	1	0	10	3	SL
2009	1	42	2	0	0	1	0.3	1	0	0	3	1	SS
2010	2	20	1	0	1.17	2	0.6	1	4	2	3	1	SL
2011	2	22	1	1	2	3	2	3	5	2	5	2	SL
2012	2	19	1	1	0.67	1	0.4	1	3	2	0	1	SL
2013	2	35	2	1	3	3	3	3	2	1	15	3	SL
2014	2	20	1	1	2.33	3	1.9	2	0	0	5	2	SL
2015	2	47	2	1	1.67	2	3	3	0	0	4	2	LL
2016	2	18	1	0	1.33	2	0.3	1	0	0	1	1	SL
2017	2	38	2	1	1.33	2	2.3	3	4	2	7	3	LL
2018	2	39	2	1	2.5	3	2.1	3	1	0	1	1	SS
2019	2	22	1	1	0.8	1	1.6	2	2	1	6	2	SL
2020	2	34	2	1	3	3	2.8	3	1	0	3	1	SS
2021	1	21	1	0	0	1	0.1	1	1	0	1	1	SS
2022	1	21	1	1	1	2	1.3	2	6	2	10	3	LL
2023	2	45	2	1	1.83	2	3.2	3	2	1	6	2	SS
2024	2	18	1	0	2.33	3	1.6	2	4	2	0	1	SL
2025	1	18	1	0	0.33	1	1.6	2	2	1	2	1	SL
2026	2	22	1	1	0.67	1	1.3	2	1	0	13	3	SL
2027	2	41	2	1	0.17	1	1.3	2	1	0	7	3	SL
2028	2	31	2	1	2	3	3.2	3	1	0	6	2	LL
2029	2	25	1	1	0.17	1	0.4	1	4	2	6	2	SS
2030	1	45	2	1	3	3	3.5	3	0	0	3	1	SL
2031	2	37	2	1	1.83	2	2.4	3	0	0	8	3	SL
2032	2	21	1	1	1.33	2	1	2	5	2	9	3	SL
2033	2	26	1	1	2	3	1.6	2	2	1	8	3	SL
2034	2	21	1	1	0	1	1	2	3	2	0	1	LL
2035	2	37	2	0	3.17	3	3	3	0	0	15	3	LL
2036	2	45	2	1	0	1	0.6	1	1	0	4	2	SL
2037	2	32	2	1	1	2	0.7	1	0	0	3	1	LL
2038	1	18	1	1	1.67	2	3.7	3	1	0	1	1	SL
2039	2	31	2	1	1	2	0.5	1	1	0	2	1	SL
2040	2	20	1	1	0.67	1	1.5	2	2	1	7	3	SL
2041	2	19	1	0	0.17	1	0.7	1	0	0	1	1	LL
2042	2	30	1	1	0	1	0.9	1	1	0	2	1	SL
2043	2	19	1	0	1.67	2	0.65	1	3	2	7	3	SL
2044	2	31	2	1	0.83	1	0.3	1	1	0	1	1	SL
2045	2	40	2	0	1	2	0.8	1	2	1	2	1	SL
2046	2	27	1	1	1	2	3.2	3	1	0	8	3	SS
2047	2	36	2	1	3.2	3	3.3	3	3	2	14	3	SS
2048	2	37	2	1	2.83	3	1.7	2	0	0	10	3	SS
2049	2	38	2	1	2.5	3	2.1	3	5	2	13	3	LL
2050	2	28	1	0	0.83	1	0.4	1	1	0	2	1	SL
2051	2	43	2	1	2.67	3	2.4	3	0	0	4	2	SL
2052	2	28	1	0	1	2	0.5	1	0	0	0	1	LL
2053	2	37	2	0	0.17	1	0.2	1	1	0	2	1	SL
2054	2	43	2	1	2.17	3	2.4	3	0	0	3	1	SL
2055	1	27	1	1	0.17	1	1.5	2	0	0	2	1	LL
2056	2	41	2	1	3.67	3	3.3	3	4	2	2	1	LL
2057	2	26	1	1	2.67	3	1.5	2	0	0	3	1	SL
2058	2	33	2	1	0.83	1	0.5	1	2	1	4	2	SL
2059	1	42	2	1	2.83	3	2.5	3	0	0	5	2	SL
2060	2	30	1	1	0.83	1	1.2	2	1	0	2	1	SL
2061	2	47	2	1	0.17	1	0.4	1	1	0	6	2	SL
2062	2	25	1	1	2.17	3	0.7	1	2	1	5	2	LL

2063	2	48	2	1	1.17	2	0.7	1	1	0	0	1	LL
2064	1	38	2	1	0.5	1	0.5	1	3	2	2	1	SL
2065	2	34	2	1	1.4	2	2.18	3	2	1	1	1	SL
2066	1	20	1	0	1.5	2	1.2	2	0	0	3	1	SL
2067	1	20	1	0	2.67	3	2.8	3	2	1	6	2	SL
2068	2	24	1	1	0	1	0.4	1	0	0	2	1	LL
2069	2	43	2	1	3.83	3	3.9	3	5	2	10	3	SS
2070	2	21	1	0	0.83	1	0.7	1	1	0	2	1	LL
2071	2	18	1	1	0.83	1	1.4	2	1	0	4	2	LL
2072	1	23	1	0	0.83	1	1	2	1	0	4	2	SL
2073	2	19	1	1	1	2	1.4	2	4	2	10	3	SL
2074	1	28	1	1	1.5	2	2.1	3	5	2	1	1	SS
2075	2	28	1	0	0.33	1	0	1	2	1	0	1	SL
2076	2	19	1	1	1.83	2	1.7	2	2	1	12	3	SL
2077	1	57	2	1	0	1	0.3	1	0	0	6	2	SL
2078	1	22	1	1	1.17	2	1.6	2	2	1	4	2	SL
2079	2	19	1	0	0.17	1	0.2	1	3	2	0	1	LL
2080	1	22	1	0	1	2	1.8	2	4	2	2	1	SL
2081	1	20	1	0	0	1	0.3	1	1	0	4	2	LL
2082	2	50	2	0	0.17	1	0.2	1	0	0	5	2	LL
2083	2	59	2	0	0	1	0	1	0	0	0	1	SL
2084	2	59	2	0	0.83	1	0.9	1	1	0	2	1	LL
2085	1	52	2	0	0	1	0	1	0	0	0	1	LL
2086	1	28	1	0	0	1	0.1	1	1	0	2	1	SS
2087	1	22	1	0	0.33	1	0.9	1	1	0	9	3	SS
2088	1	21	1	0	0.5	1	0.5	1	0	0	2	1	SS
2089	1	47	2	1	1	2	1.5	2	1	0	7	3	SS
2090	1	49	2	1	0.67	1	1.3	2	1	0	3	1	SL
2091	1	35	2	0	0.33	1	0.1	1	1	0	0	1	SL
2092	1	20	1	1	2.17	3	2.4	3	3	2	10	3	SL
2093	2	27	1	1	1.83	2	2	3	2	1	0	1	LL
2094	1	47	2	1	0.67	1	1.7	2	2	1	2	1	LL
2095	2	24	1	0	0.67	1	0.7	1	2	1	1	1	SL
2096	1	45	2	0	0	1	0	1	0	0	0	1	SS
2097	1	44	2	0	0.83	1	0.8	1	1	0	3	1	SS
2098	1	36	2	1	0	1	0.5	1	0	0	0	1	SS
2099	2	29	1	1	0.33	1	0.2	1	2	1	1	1	LL
2100	1	26	1	0	0	1	0	1	1	0	0	1	SL
2101	1	37	2	1	1.5	2	3.4	3	2	1	5	2	SS
2102	1	18	1	1	1.33	2	2.2	3	5	2	2	1	SS
2103	2	26	1	1	1	2	0.5	1	0	0	12	3	SS
2104	1	25	1	0	0.5	1	1.1	2	0	0	1	1	SS
2105	1	45	2	0	0.17	1	0.1	1	0	0	0	1	SL
2106	1	41	2	0	0.67	1	0.1	1	2	1	6	2	LL
2107	1	42	2	1	0.67	1	1	2	1	0	11	3	LL
2108	1	46	2	0	0.5	1	0	1	2	1	3	1	LL
2109	1	42	2	0	1	2	0.5	1	1	0	9	3	SS
2110	1	41	2	0	0.67	1	0.7	1	2	1	10	3	SL
2111	1	45	2	0	0.33	1	0.5	1	1	0	0	1	SL
2112	1	41	2	0	0.83	1	1.8	2	3	2	3	1	LL
2113	2	29	1	0	0	1	0	1	3	2	0	1	SL
2114	1	26	1	1	0.33	1	0.5	1	1	0	1	1	SS
2115	1	27	1	0	0	1	0.2	1	0	0	1	1	SS
2116	2	45	2	1	0.5	1	1	2	1	0	3	1	SL
2117	2	28	1	1	2.83	3	2.4	3	2	1	4	2	LL
2118	1	18	1	1	2.67	3	2.7	3	1	0	4	2	LL
2119	2	35	2	1	1.33	2	1	2	3	2	5	2	SS
2120	1	48	2	0	0.17	1	0.3	1	0	0	2	1	SS
2121	1	47	2	0	0.17	1	0.5	1	4	2	1	1	LL

2122	1	48	2	1	0	1	0	1	0	0	3	1	SL
2123	1	46	2	0	0.17	1	0	1	0	0	3	1	LL
2124	2	45	2	0	1.33	2	0.4	1	1	0	0	1	SL
2125	2	30	1	1	0.33	1	0.7	1	2	1	4	2	SL
2126	1	39	2	0	0.17	1	0	1	0	0	0	1	SL
2127	1	38	2	1	1.17	2	1.5	2	5	2	2	1	SS
2128	2	38	2	1	0.67	1	0.2	1	1	0	0	1	LL
2129	1	44	2	0	0	1	0.1	1	2	1	0	1	SS
2130	1	41	2	0	0.67	1	0.5	1	1	0	1	1	SL
2131	1	43	2	1	0	1	0.1	1	0	0	1	1	SL
2132	1	48	2	1	1	2	1.2	2	0	0	5	2	SL
2133	1	30	1	0	0.33	1	0.5	1	1	0	1	1	SS
2134	1	31	2	0	0	1	0	1	0	0	2	1	SL
2135	1	43	2	0	0.5	1	2.4	3	0	0	7	3	LL
2136	1	26	1	0	0.33	1	1.5	2	3	2	0	1	LL
2137	2	26	1	0	0.5	1	0.9	1	2	1	0	1	SL
2138	1	34	2	0	0	1	0.4	1	1	0	1	1	SL
2139	1	39	2	1	0.17	1	0.4	1	3	2	4	2	SL
2140	1	27	1	0	0	1	0.5	1	4	2	2	1	LL
2141	1	49	2	0	2.83	3	1	2	1	0	3	1	SL
2142	1	49	2	0	0	1	0	1	0	0	0	1	SL
2143	1	49	2	0	0	1	0.2	1	2	1	0	1	LL
2144	1	27	1	1	0.67	1	0	1	1	0	1	1	LL
2145	1	39	2	1	0	1	0.5	1	3	2	3	1	SL
2146	1	30	1	0	1	2	0.9	1	2	1	2	1	SL
2147	1	30	1	0	0.17	1	0	1	2	1	3	1	LL
2148	2	34	2	1	1.17	2	0.7	1	1	0	6	2	SL
2149	1	37	2	0	0.5	1	0.1	1	0	0	8	3	SL
2150	1	31	2	0	0.17	1	0.2	1	0	0	1	1	LL
2151	1	32	2	0	0	1	0	1	0	0	2	1	LL
2152	2	33	2	1	0.17	1	0.2	1	1	0	0	1	SL
2153	1	32	2	0	2.5	3	0.6	1	3	2	4	2	LL
2154	1	39	2	0	2	3	0.4	1	0	0	1	1	LL
2155	1	47	2	0	0	1	0.2	1	1	0	0	1	SS
2156	1	25	1	1	0.5	1	0.4	1	0	0	7	3	SL
2157	1	36	2	1	1	2	1.2	2	2	1	5	2	LL
2158	1	35	2	0	0.17	1	0.6	1	0	0	2	1	LL
2159	2	22	1	1	3.33	3	3.5	3	5	2	3	1	SL
2160	2	39	2	1	2	3	2.6	3	0	0	0	1	SL
2161	1	40	2	1	0.83	1	1.3	2	0	0	0	1	SS
2162	1	35	2	0	0	1	0.2	1	1	0	1	1	SL
2163	2	35	2	0	0.33	1	0.6	1	1	0	1	1	LL
2164	1	45	2	1	0	1	0.1	1	1	0	2	1	SL
2165	1	28	1	0	1	2	0	1	0	0	0	1	LL
2166	1	33	2	1	0	1	0.9	1	2	1	1	1	LL
2167	2	22	1	1	1.17	2	2.2	3	1	0	1	1	LL
2168	1	25	1	0	0.17	1	0	1	0	0	3	1	SL
2169	1	21	1	0	0.5	1	0.3	1	1	0	1	1	SS
2170	1	49	2	0	0.83	1	0	1	1	0	2	1	SL
2171	1	41	2	0	0.67	1	0.4	1	0	0	5	2	SL
2172	1	35	2	0	0	1	0.1	1	0	0	2	1	LL
2173	1	35	2	1	1	2	1.5	2	1	0	10	3	SL
2174	1	27	1	0	0.17	1	0.2	1	2	1	0	1	SS
2175	1	43	2	1	3.83	3	3.1	3	3	2	11	3	SL
2176	1	30	1	0	0.17	1	0.1	1	0	0	0	1	LL
2177	1	33	2	0	0	1	0.1	1	1	0	7	3	SL
2178	2	34	2	1	0.33	1	0.4	1	2	1	8	3	SL
2179	1	38	2	0	0.67	1	1.1	2	0	0	2	1	SL
2180	1	21	1	1	2.83	3	2.7	3	2	1	3	1	SL

2181	1	46	2	1	1	2	1	2	1	0	5	2	SL
2182	2	39	2	0	0.67	1	0.4	1	3	2	3	1	LL
2183	1	42	2	0	0.83	1	0.5	1	2	1	2	1	SL
2184	1	40	2	0	0	1	0.1	1	1	0	1	1	SL
2185	1	31	2	1	0	1	0.4	1	1	0	13	3	LL
2186	2	24	1	1	0.5	1	0.4	1	2	1	1	1	SL
2187	2	49	2	1	0.5	1	1.2	2	2	1	0	1	LL
2188	1	33	2	0	0.17	1	0	1	0	0	1	1	LL
2189	1	38	2	0	0.17	1	0.2	1	0	0	2	1	LL
2190	1	48	2	1	0	1	0.6	1	3	2	6	2	SL
2191	2	28	1	1	3.67	3	3.4	3	8	2	9	3	SL
2192	1	20	1	1	3	3	2.7	3	3	2	1	1	LL
2193	1	48	2	1	3.17	3	2.1	3	0	0	4	2	SL
2194	1	18	1	1	0.83	1	0.3	1	1	0	10	3	SS
2195	1	20	1	0	2.67	3	2.5	3	0	0	4	2	SL
2196	1	38	2	0	0	1	0.4	1	1	0	4	2	SL
2197	1	23	1	0	0.67	1	0.8	1	3	2	3	1	SL
2198	1	32	2	0	0	1	0.5	1	2	1	0	1	SL
2199	1	20	1	0	0.33	1	0.1	1	3	2	1	1	LL
2200	2	36	2	1	3	3	2	3	2	1	1	1	SS
2201	1	34	2	1	1.17	2	2	3	0	0	2	1	LL
2202	1	32	2	0	0	1	0.4	1	1	0	6	2	SL
2203	1	24	1	0	0	1	0.1	1	1	0	4	2	SL
2204	2	20	1	1	1.5	2	2.4	3	2	1	7	3	SL
2205	1	33	2	0	0.17	1	0.1	1	1	0	2	1	SS
2206	1	49	2	0	0.17	1	0.6	1	0	0	2	1	LL
2207	1	60	2	0	0.5	1	0.3	1	1	0	3	1	SL
2208	1	37	2	1	2.33	3	1.6	2	2	1	10	3	SL
2209	1	47	2	1	0.17	1	0	1	0	0	12	3	LL
2210	1	19	1	0	0.17	1	0.8	1	4	2	2	1	SL
2211	1	32	2	1	1	2	0.3	1	2	1	2	1	LL
2212	1	39	2	0	0.83	1	1.8	2	1	0	4	2	LL
2213	1	36	2	1	0.33	1	0.7	1	2	1	1	1	SL
2214	1	40	2	0	0.33	1	0.3	1	1	0	3	1	LL
2215	1	43	2	0	0	1	0.3	1	1	0	0	1	SL
2216	1	47	2	0	0.33	1	0.2	1	0	0	3	1	LL
2217	1	40	2	1	0	1	0.6	1	1	0	4	2	SL
2218	1	18	1	1	2.17	3	1.1	2	3	2	3	1	LL
2219	1	36	2	0	0.33	1	0.4	1	1	0	0	1	LL
2220	1	35	2	0	0.33	1	0.7	1	0	0	2	1	SL
2221	2	20	1	1	3.17	3	3.7	3	2	1	1	1	SL
2222	1	41	2	0	0.17	1	0.3	1	0	0	8	3	LL
2223	1	21	1	0	0.33	1	0.5	1	1	0	3	1	LL
2224	1	31	2	0	0.33	1	0.6	1	4	2	0	1	LL
2225	1	25	1	0	0.5	1	1.5	2	0	0	2	1	LL
2226	1	49	2	0	0	1	0	1	0	0	8	3	SL
2227	1	48	2	0	0	1	0.2	1	0	0	3	1	SS
2228	2	47	2	1	3.83	3	3.2	3	2	1	10	3	LL
2229	1	21	1	0	0.17	1	1.3	2	0	0	2	1	SL
2230	1	34	2	1	1.17	2	0.48	1	0	0	3	1	SS
2231	1	31	2	1	0.5	1	0.3	1	1	0	0	1	SL
2232	1	35	2	0	1.83	2	0.5	1	0	0	0	1	LL
2233	1	32	2	0	0.17	1	0.1	1	1	0	0	1	SL
2234	1	26	1	0	0	1	0.4	1	4	2	3	1	LL
2235	2	34	2	0	0.33	1	0	1	1	0	1	1	SS
2236	1	18	1	1	2.17	3	1.6	2	0	0	2	1	SL
2237	2	43	2	0	0.33	1	0.9	1	2	1	2	1	SL
2238	1	48	2	0	0	1	0	1	2	1	1	1	LL
2239	1	49	2	0	1	2	0.4	1	0	0	6	2	SS

2240	1	47	2	0	0.33	1	0	1	0	0	0	1	SL
2241	1	45	2	0	0	1	0	1	0	0	2	1	SL
2242	1	49	2	1	0.83	1	0.6	1	1	0	11	3	SS
2243	1	32	2	0	0.67	1	0.7	1	2	1	2	1	LL
2244	1	27	1	0	0	1	0	1	1	0	4	2	SL
2245	2	25	1	0	0	1	0	1	0	0	2	1	LL
2246	2	47	2	1	1.33	2	1.7	2	4	2	5	2	LL
2247	1	36	2	0	0	1	0	1	1	0	0	1	SS
2248	2	44	2	1	0	1	0	1	3	2	1	1	SL
2249	1	30	1	1	0.33	1	1.2	2	1	0	#NULL!	#NULL!	SL
2250	1	20	1	0	0	1	0	1	1	0	0	1	LL
2251	1	44	2	1	0.33	1	0.4	1	0	0	2	1	LL
2252	1	27	1	0	0	1	0.7	1	0	0	1	1	LL
2253	1	47	2	0	1.17	2	0	1	3	2	2	1	SL
2254	1	41	2	0	0.17	1	0.1	1	1	0	1	1	SL
2255	1	44	2	0	0	1	0.3	1	1	0	1	1	LL
2256	1	49	2	1	2.33	3	1.2	2	1	0	5	2	LL
2257	1	46	2	0	0	1	0	1	1	0	0	1	SL
2258	2	44	2	1	0.5	1	0.5	1	2	1	3	1	LL
2259	1	22	1	0	0.67	1	0	1	0	0	0	1	SL
2260	1	47	2	0	1.67	2	1.1	2	1	0	8	3	LL
2261	1	18	1	0	2.67	3	2.7	3	3	2	3	1	SL
2262	1	38	2	0	0.5	1	0.2	1	1	0	2	1	LL
2263	2	29	1	0	0	1	0.1	1	2	1	0	1	LL
2264	1	19	1	1	0.67	1	0.7	1	1	0	8	3	LL
2265	1	47	2	0	0	1	0	1	1	0	0	1	LL
2266	1	45	2	1	3.33	3	2.6	3	0	0	4	2	SL
2267	1	32	2	1	0.5	1	1.2	2	1	0	2	1	SL
2268	2	43	2	1	2	3	3.1	3	3	2	3	1	LL
2269	1	38	2	0	0.17	1	0	1	0	0	1	1	SL
2270	2	44	2	1	2.17	3	2.2	3	0	0	3	1	SS
2271	1	24	1	1	2.17	3	3.2	3	6	2	3	1	LL
2272	1	48	2	1	1.33	2	2	3	1	0	7	3	LL
2273	2	24	1	0	0.5	1	0.4	1	0	0	0	1	SL
2274	2	24	1	1	1.17	2	1.9	2	4	2	4	2	LL
2275	1	32	2	0	0	1	0.1	1	0	0	2	1	SL
2276	1	20	1	0	0	1	0.8	1	1	0	0	1	LL
2277	1	33	2	1	0.5	1	1.3	2	1	0	3	1	LL
2278	2	23	1	0	2.5	3	1.8	2	2	1	10	3	SL
2279	2	42	2	1	0.5	1	0.6	1	1	0	12	3	LL
2280	2	45	2	1	0.17	1	0.1	1	1	0	2	1	LL
2281	2	34	2	1	0.83	1	1	2	1	0	3	1	LL
2282	2	45	2	1	1.17	2	2	3	3	2	9	3	LL
2283	2	24	1	0	0	1	0.5	1	0	0	2	1	SS
2284	2	49	2	1	2.5	3	2.2	3	0	0	6	2	SL
2285	2	23	1	1	3.17	3	3.5	3	2	1	1	1	SS
2286	2	35	2	1	3.2	3	1.4	2	1	0	8	3	SS
2287	1	20	1	0	0.5	1	0.2	1	1	0	2	1	SL
2288	1	20	1	0	0.83	1	0.5	1	0	0	0	1	SS
2289	2	19	1	1	2.33	3	1.8	2	0	0	2	1	LL
2290	1	21	1	1	0.83	1	0.7	1	2	1	6	2	SL
2291	2	23	1	1	0.5	1	0.6	1	2	1	3	1	SL
2292	1	23	1	0	0	1	0	1	0	0	0	1	SL
2293	1	24	1	0	0	1	0.1	1	1	0	4	2	SS
2294	2	22	1	0	0	1	0.2	1	0	0	3	1	SL
2295	2	22	1	0	0.17	1	0.7	1	3	2	4	2	LL
2296	2	22	1	0	0.33	1	0.6	1	1	0	1	1	SS
2297	2	20	1	0	1.33	2	0.6	1	2	1	1	1	SS
2298	1	36	2	0	0	1	0	1	0	0	1	1	SL

2299	1	58	2	1	0.17	1	2.6	3	1	0	10	3	SL
2300	2	30	1	0	0.33	1	0.4	1	1	0	4	2	SS
2301	2	53	2	1	0.33	1	0	1	0	0	1	1	LL
2302	1	55	2	0	0.33	1	0.2	1	1	0	1	1	SL
2303	1	60	2	1	1	2	1.2	2	1	0	4	2	LL
2304	2	58	2	1	3.17	3	3.2	3	1	0	0	1	LL
2305	1	57	2	1	1.5	2	0.1	1	0	0	9	3	LL
2306	2	53	2	1	1.83	2	1.2	2	0	0	2	1	LL
2307	1	60	2	1	0.17	1	0.2	1	2	1	8	3	LL
2308	1	#NULL!	#NULL!	1	0	1	0	1	0	0	0	1	SL
2309	2	18	1	0	0.33	1	0.4	1	2	1	6	2	LL
2310	2	51	2	1	4	3	3.6	3	2	1	11	3	SS
2311	2	54	2	1	0	1	0.4	1	2	1	5	2	SL
2312	2	54	2	1	2.67	3	2	3	2	1	5	2	SS
2313	2	38	2	1	1.5	2	1.4	2	1	0	3	1	SS
2314	1	57	2	0	0.17	1	0.1	1	1	0	0	1	SL
2315	1	56	2	0	1.83	2	1.1	2	1	0	10	3	SL
2316	2	58	2	1	0	1	0.6	1	2	1	13	3	LL
2317	2	56	2	1	2.5	3	3.2	3	1	0	13	3	LL
2318	2	27	1	0	2	3	1.5	2	0	0	5	2	SL
2319	2	56	2	1	2.17	3	1.9	2	0	0	4	2	SL
2320	2	60	2	1	2	3	1.7	2	0	0	4	2	SL
2321	1	#NULL!	#NULL!	1	1	2	0.7	1	2	1	4	2	SL
2322	1	55	2	1	1.17	2	1.2	2	0	0	11	3	SL
2323	1	22	1	0	0.33	1	0.1	1	0	0	1	1	SS
2324	2	52	2	1	3.33	3	3.1	3	2	1	2	1	LL
2325	1	60	2	1	1	2	1.1	2	0	0	6	2	SL
2326	2	51	2	1	1.83	2	1.1	2	3	2	10	3	LL
2327	1	30	1	0	0.83	1	0.6	1	0	0	1	1	SL
2328	2	57	2	1	0.33	1	0.5	1	2	1	3	1	LL
2329	2	60	2	1	0.5	1	0.98	1	0	0	11	3	SL
2330	2	58	2	0	0.33	1	0.2	1	1	0	8	3	SS
2331	2	54	2	1	0.33	1	0	1	2	1	2	1	LL
2332	2	60	2	0	0.83	1	1.4	2	0	0	0	1	SL
2333	1	47	2	1	0.17	1	0.9	1	0	0	6	2	SS
2334	1	26	1	0	0.17	1	0	1	0	0	1	1	SL
2335	2	58	2	1	2.17	3	2.1	3	0	0	15	3	SL
2336	2	37	2	0	0.33	1	0.4	1	0	0	8	3	LL
2337	1	57	2	1	1	2	0.3	1	1	0	2	1	SL
2338	2	56	2	1	1.17	2	0.4	1	0	0	0	1	LL
2339	2	34	2	1	1.5	2	3.2	3	4	2	8	3	SS
2340	2	26	1	0	0.17	1	0.5	1	0	0	3	1	LL
2341	2	52	2	1	0	1	0.1	1	0	0	3	1	LL
2342	2	54	2	1	1.17	2	0.2	1	2	1	3	1	SS
2343	2	58	2	1	1	2	1	2	4	2	10	3	LL
2344	1	#NULL!	#NULL!	1	1	2	0.3	1	2	1	5	2	SL
2345	2	24	1	1	2.5	3	2.7	3	3	2	0	1	SS
2346	2	56	2	1	2.67	3	2.7	3	1	0	9	3	LL
2347	1	40	2	1	2	3	3.7	3	2	1	5	2	LL
2348	1	55	2	0	0	1	0.1	1	1	0	7	3	SS
2349	1	50	2	1	0.33	1	2.1	3	2	1	1	1	SL
2350	2	23	1	1	1	2	1.1	2	3	2	2	1	LL
2351	2	25	1	1	1.6	2	1.5	2	1	0	10	3	SL
2352	1	31	2	0	0	1	0.2	1	0	0	0	1	LL
2353	1	35	2	1	0.67	1	1.1	2	0	0	4	2	SL
2354	2	23	1	1	1.17	2	0.4	1	0	0	2	1	SL
2355	2	34	2	1	2.33	3	1.3	2	1	0	5	2	SL
2356	2	25	1	1	3	3	1.2	2	0	0	2	1	SL
2357	1	52	2	1	1.67	2	1.3	2	0	0	2	1	SL

2358	2	36	2	1	0.83	1	0.4	1	3	2	4	2	SL
